# Knockout Mice for Dyslexia Susceptibility Gene Homologs *KIAA0319* and *KIAA0319L* have Unaffected Neuronal Migration but Display Abnormal Auditory Processing

**DOI:** 10.1093/cercor/bhx269

**Published:** 2017-10-17

**Authors:** Luiz G Guidi, Jane Mattley, Isabel Martinez-Garay, Anthony P Monaco, Jennifer F Linden, Antonio Velayos-Baeza, Zoltán Molnár

**Affiliations:** 1 Department of Physiology, Anatomy, and Genetics, University of Oxford, Oxford OX1 3QX, UK; 2 Wellcome Trust Centre for Human Genetics, University of Oxford, Oxford OX3 7BN, UK; 3 Ear Institute, University College London, London WC1X 8EE, UK; 4 Department of Neuroscience, Physiology & Pharmacology, University College London, London WC1E 6BT, UK; 6 Current address: Office of the President, Ballou Hall, Tufts University, Medford, MA 02155, USA

**Keywords:** auditory function, cerebral cortex, dyslexia, neuronal migration, temporal processing

## Abstract

Developmental dyslexia is a neurodevelopmental disorder that affects reading ability caused by genetic and non-genetic factors. Amongst the susceptibility genes identified to date, *KIAA0319* is a prime candidate. RNA-interference experiments in rats suggested its involvement in cortical migration but we could not confirm these findings in *Kiaa0319*-mutant mice. Given its homologous gene *Kiaa0319L* (*AU040320*) has also been proposed to play a role in neuronal migration, we interrogated whether absence of AU040320 alone or together with KIAA0319 affects migration in the developing brain. Analyses of *AU040320* and double *Kiaa0319;AU040320* knockouts (dKO) revealed no evidence for impaired cortical lamination, neuronal migration, neurogenesis or other anatomical abnormalities. However, dKO mice displayed an auditory deficit in a behavioral gap-in-noise detection task. In addition, recordings of click-evoked auditory brainstem responses revealed suprathreshold deficits in wave III amplitude in *AU040320*-KO mice, and more general deficits in dKOs. These findings suggest that absence of AU040320 disrupts firing and/or synchrony of activity in the auditory brainstem, while loss of both proteins might affect both peripheral and central auditory function. Overall, these results stand against the proposed role of KIAA0319 and AU040320 in neuronal migration and outline their relationship with deficits in the auditory system.

## Introduction

The capacity for language is one of the key features underlying the complexity of human cognition and its evolution. However, little is known about the neurobiological mechanisms of linguistic ability. Developmental dyslexia refers to a specific impairment in reading ability despite adequate intelligence, educational opportunity and lack of obvious sensory abnormalities, and it is one of the most common neurodevelopmental disabilities, affecting 5–12% of school-aged children (Paracchini et al. 2007; Peterson and Pennington 2015).

The specific nature of the neuropsychological mechanisms underlying dyslexia remains highly controversial (Shaywitz and Shaywitz 2005; Ramus and Ahissar 2012; Goswami 2014). A specific impairment in the phonological system is widely held to be one of its primary causes (Goswami 2000; Ramus and Ahissar 2012; Boets et al. 2013), but this view is challenged by evidence that dyslexics suffer from subtle sensory dysfunction, particularly in the auditory domain (Ramus 2003; Tallal 2004; Shaywitz and Shaywitz 2005; Ahissar et al. 2006; Ziegler et al. 2009; Ramus and Ahissar 2012; Goswami 2014). It is generally considered that disorders of language, including dyslexia, result from impaired structure and/or function of the neocortex (Shaywitz and Shaywitz 2008; Norton et al. 2015), but other brain regions have also been implicated in dyslexia such as the cerebellum (Nicolson et al. 2001) and the auditory brainstem (Hornickel and Kraus 2013; White-Schwoch et al. 2016; Neef et al. 2017).

Dyslexia is considered a complex, multi-factorial disorder and behavioral genetics studies have revealed a significant genetic component in its etiology, with heritability estimates at 40–70% (Paracchini et al. 2007). Several dyslexia susceptibility loci and candidate genes have been identified, with *DYX1C1*, *DCDC2, KIAA0319*, and *ROBO1* established as the main candidates (Carrion-Castillo et al. 2013). Amongst these, *KIAA0319* emerges as a prime candidate based on consistently replicated associations in independent samples (Carrion-Castillo et al. 2013) and functional evidence linking dyslexia susceptibility to transcriptional regulation of *KIAA0319* (Paracchini et al. 2006; Dennis et al. 2009). Interestingly, the paralogous gene *KIAA0319L* (or *KIAA0319-Like*), the only other member of this gene family, has also been linked to dyslexia (Couto et al. 2008).

Both *KIAA0319* and *KIAA0319L* have been previously implicated in neuronal migration during the development of the neocortex. Experiments using in utero knockdown with shRNA against the homologous rat genes, *Kiaa0319* and *Kiaa0319-Like*, have found that altered levels of expression of either of these 2 genes can affect the migration of cortical neurons and lead to periventricular heterotopias (Paracchini et al. 2006; Peschansky et al. 2010; Szalkowski et al. 2012; Adler et al. 2013; Platt et al. 2013). Similar results have been found in experiments targeting homologs of the other main dyslexia susceptibility genes, *Dyx1c1*, *Dcdc2*, and *Robo1* (Meng et al. 2005; Burbridge et al. 2008; Adler et al. 2013; Gonda et al. 2013). These findings parallel early observations made from post-mortem histopathological examination of dyslexic brains reporting anatomical abnormalities such as cortical ectopias, heterotopias, and cortical dysplasia (Galaburda and Kemper 1979; Galaburda et al. 1985; Humphreys et al. 1990). The combination of these findings has led to the formulation of the hypothesis that dyslexia is a neuronal migration disorder (Galaburda et al. 1985, 2006; Paracchini et al. 2007; Gabel et al. 2010).

The *KIAA0319* and *KIAA0319L* genes encode highly homologous (61% similar) proteins that localize to the plasma membrane (Velayos-Baeza et al. 2007, 2008; Poon et al. 2011). KIAA0319 has been shown to undergo proteolytic processing (Velayos-Baeza et al. 2010), and both proteins follow the classic clathrin trafficking pathway (Levecque et al. 2009, and unpublished data) which likely mediates endocytosis of KIAA0319L in its role as a receptor for adeno-associated virus (Pillay et al. 2016).

In contrast to the results of the above mentioned shRNA studies, we have recently reported that mice carrying partial or total elimination of KIAA0319 did not exhibit abnormalities in neuronal migration or in the general development and organization of the neocortex (Martinez-Garay et al. 2017). Given that KIAA0319L has also been implicated in neuronal migration (Platt et al. 2013) and is homologous with KIAA0319, we hypothesized that compensation by KIAA0319L could be responsible for the normal cortical migration observed in *Kiaa0319* KO mice.

To test this hypothesis, we generated mice carrying a loss-of-function mutation in the mouse homolog of *KIAA0319L*, the *AU040320* gene, and performed a detailed examination of the developmental trajectory of the neocortex in mice lacking either AU040320 alone or in conjunction with KIAA0319. We also tested KO mice in a number of behavioral paradigms and performed recordings of auditory brainstem responses (ABRs) to test for spatial memory and auditory processing deficits similar to those reported in *Kiaa0319*-shRNA-treated rats (Szalkowski et al. 2012; Centanni et al. 2014a, 2014b). It is important to stress that these mouse KOs are not models of dyslexia, but valuable tools to investigate the function of the genes of interest. There is an obvious difficulty in linking the function of candidate genes with neural and behavioral function in mouse and human, but relevant insight can be gained that may contribute to a better understanding of the possible neurodevelopmental mechanisms involved in dyslexia.

Our results indicate that absence of KIAA0319 or AU040320 individually or in combination produces no obvious abnormalities in neuronal migration or cortical anatomy; however, absence of AU040320 disrupts auditory processing within the auditory brainstem and has more widespread effects on auditory function when combined with absence of KIAA0319.

## Materials and Methods

### Experimental Animals

Animals were housed with ad libitum access to food and water under a 12 h light/dark cycle with temperature and humidity kept constant. Animal generation, maintenance and procedures took place at the Biomedical Services buildings of the University of Oxford, with the exception of ABR recordings which were conducted at the Ear Institute, University College London (UCL). All animal experiments were approved by the local ethical boards and followed the regulations detailed in personal and project licenses approved in accordance with the Animals (Scientific Procedures) Act 1986.

**Figure 1.**
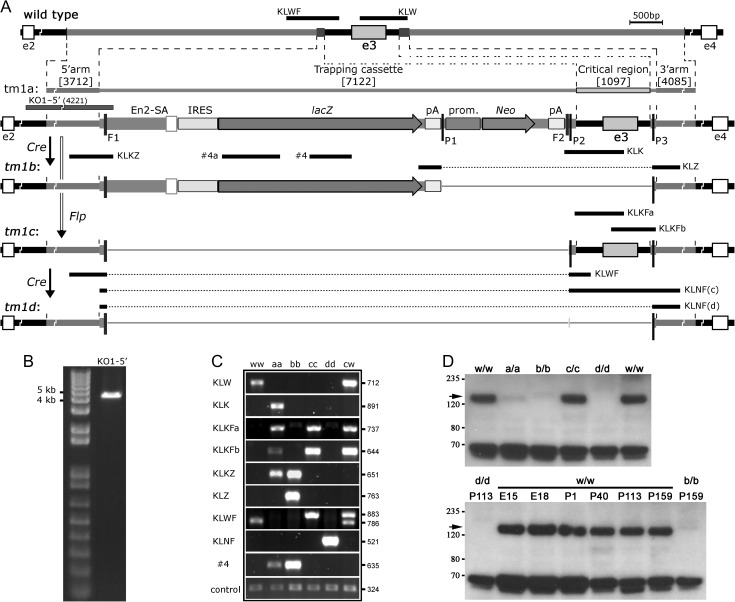
Generation of *AU040320*-targeted mice. (*A*) Schematic representation of *AU040320*-targeting strategy. A “knock-out first” (*tm1a* or KO1) allele was generated by EUCOMM in ES cells after integration of a targeting vector containing exon 3 and ~4 kb flanking fragments each side, with 2 small regions replaced by a trapping cassette (containing 2 loxP sites (P1, P2) and 2 FRT sites (F1, F2)) and a loxP-cassette (containing a loxP site (P3)), respectively. Live mice carrying this allele were obtained and used to generate other alleles (full details in Materials and Methods section). A “*del*” (*tm1b*) allele was obtained by deletion of the P1-to-P3 region after Cre recombination. After Flp recombination on the *tm1a* allele, a “floxed” (*tm1c* or Flx) allele was generated by deletion of the F1–F2 region. The Flx allele has conditional knockout potential, with the removal of the critical region containing exon 3 between sites P2 and P3 to obtain a “Null” (*tm1d* or Null) allele after Cre recombination. A long-range PCR fragment (KO1-5′) used for target confirmation in *tm1a* allele, and the genotyping PCRs used for identification of the different alleles are represented. Details about elements in the targeting cassette and the different PCRs are shown in Fig. S2 and Table S2. (*B*) Long-range PCR KO1-5′ from KO1 homozygous mouse confirming specific insertion of targeting construct in the *AU040320 locus*. DNA ladder and size of 2 bands are shown on the left. (*C*) Results of genotyping PCRs from mice homozygous for wild-type (ww), *KO1* (aa), *del* (bb), *Flx* (cc) or *Null* (dd) alleles; heterozygous *Flx* (cw) is included to show the expected double band with PCR KLWF. Size of fragments (bp) appears on the right. (*D*) Western blotting analysis from mouse brain lysates with specific antiserum KL-FCt-G1 (#78). Comparison of samples from wild-type and all 4 homozygous *AU040320*-targeted adult (16–24 week-old) male mice (top panel) shows that the AU040320 protein (arrow), with an apparent ~150 kDa size, is clearly detected in wild-type (w/w) and *Flx* (c/c), heavily reduced in *KO1* (a/a) and totally absent in *del* (b/b) and Null (d/d) samples. The ~65 kDa band detected at the bottom of the picture is the strongest of several unspecific bands detected by this antibody (see Fig. S1*A*). Same results were obtained with samples from female mice (not shown). The AU040320 protein is detected in brain samples from wild-type mice at different developmental stages (bottom panel), including embryonic days 15 (E15) and 18 (E18), and a wide range of postnatal days (P1-to-P159); adult Null (left) and del (right) samples are shown for comparison purposes. 30 μg total protein loaded per lane.

Mouse lines targeted at the *AU040320* gene were obtained as described in Supplementary Information. Briefly, ES cells targeted with a “knockout-first” (KO1, reporter-tagged insertion with conditional potential) allele (C57BL/6 N-*AU040320*^*tm1a(EUCOMM)Wtsi*^) (Skarnes et al. 2011) were purchased from the European Conditional Mouse Mutagenesis Program (EUCOMM, www.eucomm.org) and used for C57BL/6 J blastocyst injections. C57BL/6 J-*AU040320*^*tm1a(EUCOMM)Wtsi*^ (*AU040320-KO1*) mice were obtained after breeding of a male chimera with C57BL/6 J females. Other mouse lines carrying alleles *tm1b, tm1c*, and *tm1d* (Skarnes et al. 2011) were obtained: C57BL/6 J-*AU040320*^*tm1b(EUCOMM)Wtsi*^ (*lacZ*-tagged null allele; *-del*), C57BL/6 J-*AU040320*^*tm1c(EUCOMM)Wtsi*^ (floxed, conditional allele; *-Flx*), and C57BL/6 J-*AU040320*^*tm1d(EUCOMM)Wtsi*^ (null allele; *-Null*) (Fig. 1*A*). All lines were maintained on a C57BL/6J background, in which the C57BL/6N background from the original ES cells had been diluted during the successive backcrosses. Studies were conducted using the *AU040320-del* line (heterozygous and homozygous mice referred to as +/− and −/−, respectively), unless otherwise stated, with wild-type littermates used as controls. *Kiaa0319* and *AU040320* double KO (dKO) and double floxed (dFlx) lines, carrying the *tm1b/del* allele and the *tm1c/Flx* allele for both genes, respectively, were generated after crossing of the above mentioned *AU040320* lines with the previously described *Kiaa0319* lines, also kept on a C57BL/6J background (Martinez-Garay et al. 2017); only double homozygous animals were used for the different experiments. Given the extremely low probability of obtaining double KO and wild-type animals in the same litter from double heterozygous matings, double KOs were generated by mating [*Kiaa0319 *−/−*; AU040320 *+/−] mice and wild-type controls were obtained from intercrosses between 2 *AU040320* +/− parents.

### PCR, Genotyping, Sequencing and Western Blotting

Extraction of genomic DNA, amplification of genomic fragments and sequencing, preparation of protein lysates and analysis by Western blotting were performed as described previously (Martinez-Garay et al. 2017). The primers used for this study are listed in Table S1. A custom polyclonal antiserum (KL-FCt-G1 (or #78), Velayos-Baeza et al, in preparation), raised in guinea-pig after immunization with the cytosolic domain of the human KIAA0319L protein, was used for detection of the mouse AU040320 protein.

### Histology, Immunohistochemistry and In Utero Electroporations

Brain samples were collected and processed as described in Martinez-Garay et al. (2017), embedded in 4% agarose and sectioned at 50 or 100 μm using a vibrating microtome (VT1000S, Leica Systems). At least 3 matching sections per animal were selected and pre-incubated at room temperatures (RT) for 2 h in blocking solution (5% normal goat/donkey serum with 0.1% Triton X-100, with or without 4% BSA) before incubation with primary antibodies overnight at 4 °C (Table S3). Sections were then washed (3 × 10 min in PBS) and incubated with AlexaFluor-labeled antibodies (1:500 dilutions, Molecular Probes) for 2 h at RT, counterstained with DAPI and mounted with ProLong Gold Medium (Invitrogen). All antibodies were diluted in blocking solution and omission of primary antibody served as negative control. For Nissl staining, sections were mounted on gelatin-coated slides and, after defatting in a series of ethanol (95–100%) and chloroform immersion steps of 4 min each, stained with 1% cresyl violet for 5 min, washed in ethanol to dehydrate and cleared in histoclear in several 3 min steps. DePeX mounting medium (WRR) was used to mount with glass coverslips. Every third 50 μm section was analyzed for the presence of ectopias in 6 brains per genotype. In utero electroporation experiments were performed as described in Martinez-Garay et al. (2017) using the same expression constructs pCIG-GFP and pCIG-IRES-GFP.

### Image Acquisition and Cell Quantification

All fluorescent images were acquired with a laser-scanning confocal microscope (Leica TCS SP8). Image processing and analyses were conducted using GIMP Image Editor (GIMP Development Team) and ImageJ (NIH). For quantification, 100 μm-wide cortical columns were selected from the somatosensory cortex (Allen Brain Atlas used as reference; http://www.brain-map.org/), divided into 10 bins or in relevant subdivisions and cell counting performed with ImageJ's Cell Counter plugin. Statistical comparisons used 1-way ANOVAs performed in PSPP v0.6.1 for Linux (PSPP Development Team).

### Behavioral Testing

A cohort containing 18 dKO mice and 18 wild-type controls (9 males, 9 females) was generated for behavioral testing. Due to the extremely low probability of obtaining double KO and wild-type animals in the same litter using double heterozygous crossings, mutants were derived from [*Kiaa0319* −/−; *AU040320* +/−] matings, with controls derived from crossings between *AU040320* +/− animals, from which the double mutants were derived separated by 3 generations. To maximize similarity of life experiences, matings were plug-timed and matched so that mutant and wild-type mice were born and housed together in the same cage. All pups were genotyped and weaned into cages containing 6 animals, 3 mutants and 3 controls. From this point onwards genotype was kept blind to experimenter. For single KO cohorts (*Kiaa0319* and *AU040320*), littermate animals were generated from heterozygous mating pairs, with 9 animals per genotype for the *Kiaa0319* cohort and 10 for *AU040320*. All behavioral experiments were conducted during the light phase, between 08:00 and 17:00 h. Animals were 8 weeks of age at start of testing, which lasted 5 weeks. At least one day of rest between experiments was allowed. Males were always tested before females to avoid interference by perception of estrus odors, and equipment was thoroughly cleaned with 70% ethanol and water between subjects. Statistical analyses were conducted using Unistat 5.6 (Unistat Ltd) or PSPP v0.6.1 for Linux (PSPP Development Team) using paired *t*-tests, one or 2-way ANOVAs, with repeated-measures. Significance threshold was defined as α = 0.05. For all tests, 2-way ANOVAs were conducted with genotype and sex as between-subject factors but none of them revealed an effect of sex in the measures tested. Procedures are based on previously reported protocols (Ufartes et al. 2013; Martinez-Garay et al. 2017) and are briefly described in Supplementary Information.

### ABR Measurements

*Kiaa0319* KO, *AU040320* KO, dKO, and WT mice were imported from the University of Oxford to UCL for ABR measurement. At UCL, animals were housed for 2–8 weeks in individually ventilated cages following importation, then transferred to standard mouse housing at least 3 days before testing. The cohort included 11 *Kiaa0319* KO mice (83–117 days; 6 male), 12 *AU040320* KO mice (97–127 days; 4 male), and 13 dKO mice (69–72 days; 6 male), along with 14 WT mice age-matched to the single KO mice (83–117 days; 9 male; pool of littermates of *Kiaa0319* (11) and *AU040320* (3) KOs) labeled as “sWT”, and 11 WT animals age-matched to the dKO mice (65–74 days; 6 male; obtained using the same strategy as described for behavioral testing above) labeled as “dWT”. There were no significant differences in age between any of the KO animal groups and their corresponding WT comparison group (Wilcoxon rank-sum tests, all *P* > 0.1).

ABR recordings were performed as described in Anderson and Linden (2016) (see also Willott 2006). The procedure is described in more detail in the Supplementary Information. Briefly, all ABRs were recorded from the left ear, and obtained in response to monopolar square wave clicks of 50 μs duration which varied in sound intensity from 10 to 80 dB sound pressure level (SPL; increasing in 5 or 10 dB steps). The click-evoked ABR estimate for each sound level was calculated as the mean evoked response to 500 repetitions of the click stimulus presented at a rate of 10 clicks/s (Fig. 5*A*). Additional ABR recordings were also obtained in some animals using 50–80 dB SPL clicks (in 10 dB steps) presented at a slower repetition rate (2 clicks/s, 500 repetitions), and 60 dB SPL “probe” and “reference” clicks following the offset of a 200 ms 60 dB SPL broadband noise “masker” stimulus (500 repetitions). Probe clicks were presented 20 or 50 ms after masker offset, and reference clicks always occurred 500 ms after masker offset (Fig. 5*A*).

## Results

### Generation of AU040320-deficient Mice

To study the putative role of AU040320 in brain structure and function, mice carrying modified versions of the *AU040320* allele were generated, all derived from a single male chimeric mouse. As previously done for the *Kiaa0319* mice (Martinez-Garay et al. 2017), the KO1 allele in the new mouse line was validated. The correct integration of the KO1 cassette into the *AU040320 locus* targeting exon 3 (Fig. 1*A*) was confirmed by long-range PCR after amplification of a 4.2 kb fragment of the *AU040320-KO1* allele (*tm1a, AU040320*^*KO1*^) using a primer in intron 2 upstream the 5′-homology arm present in the targeting construct, and a primer specific to the KO1 cassette to cover the entire 5′-region of recombination (Fig. 1*A*,*B*). The sequence of this allele (and all derived ones) was verified by PCR amplification and sequencing of a number of overlapping fragments (data not shown) and confirmed to contain all the expected elements.

A single *AU040320*^*KO1/+*^ male was used to generate mice carrying a global KO allele (*tm1b*; *AU040320-del*), or the *AU040320-Flx* allele (*tm1c*) with conditional KO potential. All homozygous mutants (for either *AU040320*-KO1 or *AU040320*-del alleles) were viable and with no obvious differences when compared to heterozygous or wild-type littermates. However, male mice homozygous for either of these 2 alleles (*AU040320*-KO1 or *AU040320*-del) were found to be infertile (Guidi et al., in preparation). Western blotting analyses from adult brain lysates were used to verify the effects of the different alleles on protein product. The specific band of around 150 kDa corresponding to the full-length AU040320 protein was clearly reduced in *AU040320*^*KO1/KO1*^ samples and total absence was only detected in *AU040320*^*del/del*^ lysates (Fig. 1*D*). Samples from *AU040320*^*flox/flox*^ mice displayed normal protein levels as expected (Fig. 1*D*). Western blots also revealed widespread presence of the AU040320 protein in other tissues such as lung, heart, kidney, liver, and spleen (Fig. S3*A*). The cross-reactivity with other smaller proteins in most tissues makes this antibody not suitable for equivalent immuno(histo/cyto)chemistry experiments. Given the residual presence of AU040320 protein in AU040320^KO1/KO1^ samples, mice carrying the *AU040320-del* allele were used for analyses of protein function; these are referred to here as *AU040320* +*/−*, or −/− (or mutant) mice. *AU040320*^*flox/flox*^ mice were reserved for conditional KO experiments. Allele distribution in litters from *AU040320 *+/− intercrosses revealed ratios similar to the expected 1:2:1 Mendelian rates of inheritance (from a total of 127 mice: 31% were +/+, 38% +/− and 31% −/−). Overall weight of animals used for analyses showed no differences across genotype (Table S4) and no major abnormalities were detected in the overall morphology of AU040320-deficient brains in Nissl-stained sections (Fig. S3*B*,*D*).

### Absence of AU040320 Alone or in Conjunction with KIAA0319 Does Not Alter Cortical Neurogenesis

**Figure 2.**
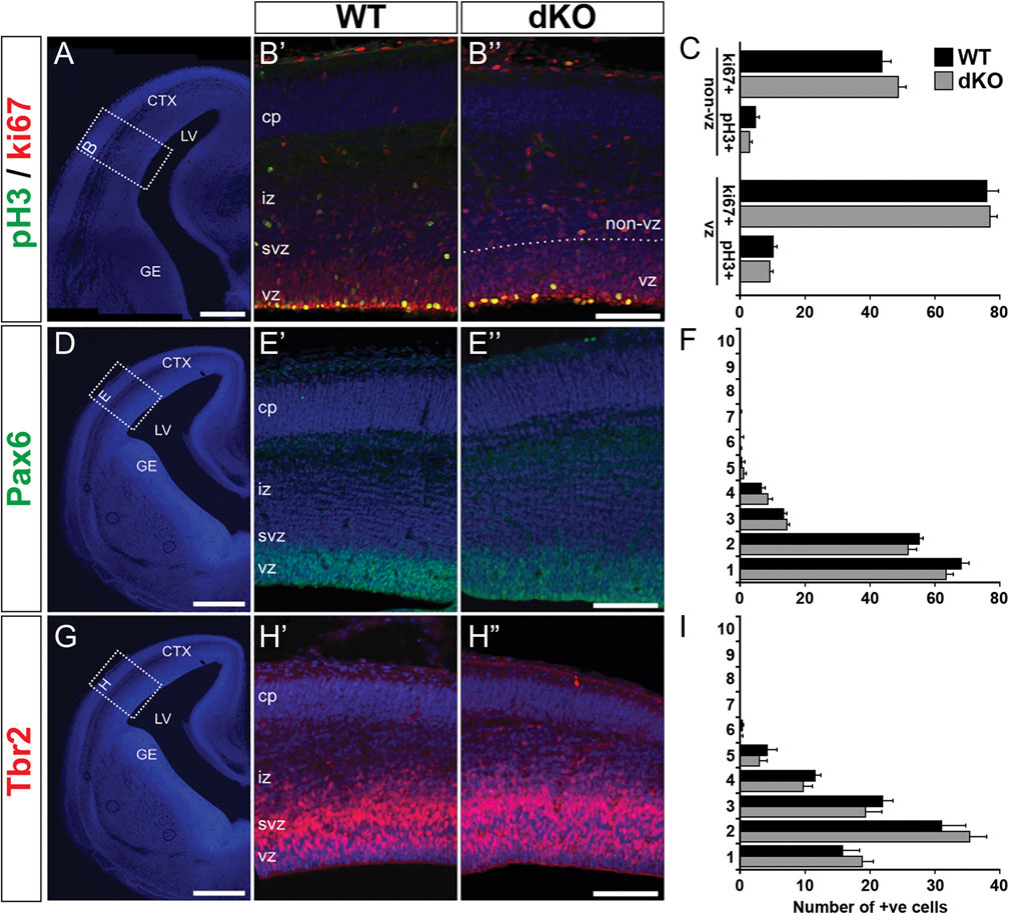
Absence of AU040320 and KIAA0319 does not alter cortical neurogenesis. (*A*, *D*, *G*) Representative DAPI images outlining regions of E15 cortices selected for analysis. (*B*) Immunolabeling of cycling cells with ki67 (red) and cells in M-phase with pH3 (green) to examine cell division profile in WT (*B*’) and dKO mice (*B*”). (*C*) Quantification of number of ph3+ and ki67+ cells in the VZ region and in the rest of the cortical wall (non-VZ); no differences were observed between the 2 genotypes (*n* = 3, *P* > 0.05). (*E*, *H*) Pools of neuronal progenitors were examined by labeling radial glial (Pax6+, green; *E*) and intermediate progenitors (Tbr2+, red; *H*). (*F*, *I*) Quantification of number of cells in each of the 10 equally sized bins dividing the cortex (in *E*, *H*) revealed no differences between dKO and WT sections for any of the conditions (*n* = 3, *P* > 0.05). All image panels show nuclear staining with DAPI. All data shown as mean ± SEM. CTX, cortex; LV, lateral ventricle; GE, ganglionic eminence; cp, cortical plate; iz, intermediate zone; svz, subventricular zone; vz, ventricular zone. Scale bars: 400 μm (*A*, *D*, *G*); 100 μm (*B*, *E*, *H*).

KIAA0319 and KIAA0319L proteins have a number of features which closely resemble those of Notch receptors (Levecque et al. 2009; Velayos-Baeza et al. 2010; Velayos-Baeza et al., unpublished), suggesting a potential role in signaling pathways. Given Notch receptors are key regulators of neurogenesis (Ables et al. 2011) and that both *AU040320* and *Kiaa0319* are expressed in the germinative area, the ventricular zone (VZ), of the developing neocortex (Paracchini et al. 2006; Diez-Roux et al. 2011), we investigated whether absence of AU040320, or in conjunction with KIAA0319, may affect the neurogenic profile in the embryonic neocortex of our mutants. Double *Kiaa0319;AU040320* KO (dKO) mice were generated and animals were viable, displaying no gross abnormalities when compared to wild-types. We assessed cell proliferation by labeling cycling and mitotic cells with antibodies against ki67 and pH3, respectively, in sections of *AU040320* mutant, dKO and wild-type cortices. Quantification of positive cells in the VZ or in the rest of the cortical wall (non-VZ) revealed no differences with controls at E15 and E18 for *AU040320* KOs (Fig. S4*A*,*B*) nor for dKOs (Fig. 2*A*–*C*, S4*C*).

We next examined whether the loss of functional copies of *AU040320* alone or together with *Kiaa0319* specifically affected the pools of neuronal progenitors in the developing neocortex by labeling radial glial progenitors with an antibody against Pax6 and intermediate progenitors with Tbr2. Pax6+ and Tbr2+ cells showed the expected clustering near the ventricular wall in both *AU040320* (Fig. S5*A*–*D*) and dKO brains (Fig. 2*D*–*I*, S5*E*,*F*), with quantification of cell number along the cortical wall revealing no changes in the distribution of progenitor populations. These results indicate that absence of AU040320 alone or in conjunction with KIAA0319 does not affect cell proliferation and neurogenesis in the developing neocortex.

### Normal Lamination in the Neocortex in *AU040320* KOs and in *Kiaa0319*;*AU040320* KOs

**Figure 3.**
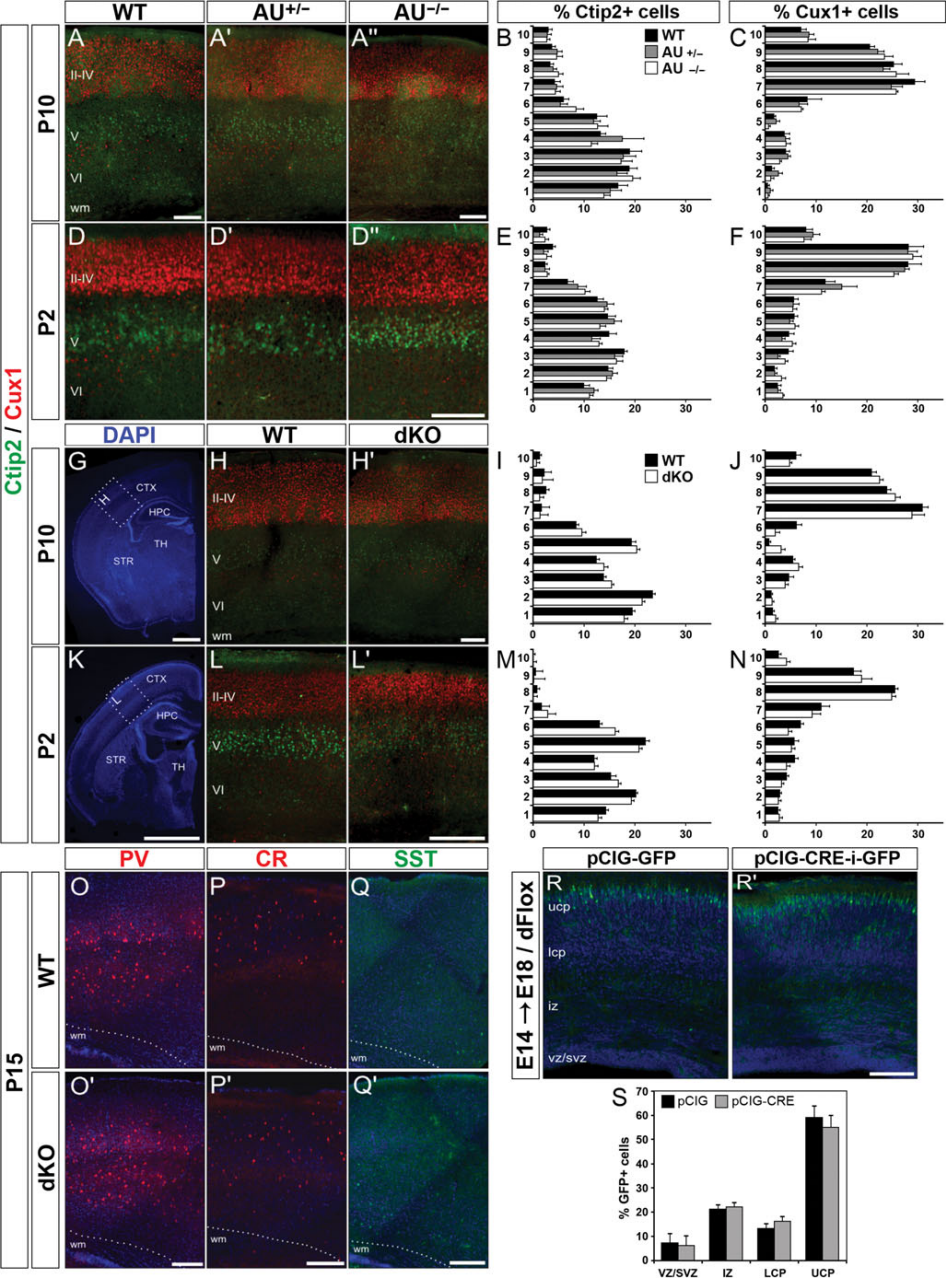
Normal cortical lamination in *AU040320* and *AU040320;Kiaa0319* KO brains. (*A*–*F*) Immunohistochemistry labeling lower layer pyramidal cells (V–VI, Ctip2; green) and upper layer ones (II–IV, Cux1+; red) in somatosensory cortex (S1) of *AU040320* mutants at P10 (*A*) and P2 (*D*). Quantification graphs for percentage of Ctip2+ and Cux1+ cells per bin at each age (*B*, *C* for P10; *E*, *F* for P2) show no differences across each condition (*n* = 3, *P* > 0.05). (*G*–*N*) Similar analyses were conducted for dKO brains. DAPI-stained images (*G*, *K*) show the cortical region (S1; dotted lines, insets) selected for quantification, for both *AU040320* and double KOs. Graphs with quantification of cell distribution per bin (*I*, *J* for P10; *M*, *N* for P2) indicate that absence of AU040320 and KIAA0319 leaves cortical lamination unaffected for all conditions (*n* = 3, *P* > 0.05). (*O*–*Q*) Three subpopulations of interneurons were examined for their overall distribution in the S1 cortex of dKO brains at P15: parvalbumin+ (PV, red; *O*) cells, calretinin+ (CR, red, *P*) and somatostatin (SST, green, *Q*). No differences were detected in any of the conditions (*n* = 3). (*R*, *S*) In utero electroporation of plasmids expressing GFP only (pCIG-GFP, *R*) and Cre recombinase with GFP (pCIG-CRE-i-GFP, *R*’) into the brains of E14 double *Kiaa0319;AU040320* floxed mice (dFlox) which were harvested at E18. Quantification of distribution (%) of cells in each subdivision of the cortical wall reveals no differences following the acute knockout of *Kiaa0319* and *AU040320* combined (*S*; *n* = 3, *P* > 0.05). Image panels *O*–*R* show nuclear staining with DAPI. All data shown as means ± SEM. AU, *AU040320*; wm, white matter; ucp, upper cortical plate; lcp, lower cortical plate; iz, intermediate zone; svz, subventricular zone; vz, ventricular zone; CTX, cortex; HPC, hippocampus; STR, striatum; TH, thalamus. Scale bars: 75 μm (*A*, *D*, *H*, *L*); 1000 μm (*G*, *K*); 150 μm (*O*–*R*).

To investigate the putative role of AU040320 in neuronal migration in more detail, we examined the laminar organization of pyramidal neurons in the cortices of *AU040320*-deficient mice by labeling cells destined to cortical layers V–VI with an antibody against Ctip2, and those destined to upper layers (II–IV) with Cux1. If AU040320 plays a role in neuronal migration, the spatial distribution of these cells would be affected in the absence of this protein. Images revealed these neuronal populations were similarly distributed in *AU040320* mutant and control brains at P2 and P10 (Fig. 3*A*–*F*), and percentage of cells per bin indicated no abnormalities (Fig. 3*B*,*C*,*E*,*F*). We also investigated whether absence of AU040320 may cause a delay in migration of these cells despite their normal final position and found no differences in their distribution between genotypes at E15 and E18 (Fig. S6*A*).

These findings are in contrast with a previous report linking AU040320 with cortical neuronal migration in rats, and it parallels our previous work showing a similar discrepancy with respect to KIAA0319 (Martinez-Garay et al. 2017). Thus, we tested for potential compensatory interactions between AU040320 and KIAA0319 by examining the laminar organization of the neocortex of dKO using the same methods as above. The distribution of Ctip2+ and Cux1+ cells in brains of dKOs did not differ from those of control samples in early postnatal stages (P10 and P2; Fig. 3*G*–*N*), nor during embryonic development (at E18; Fig S6*B*). Subplate neurons, one of the earliest neuronal populations to occupy the cerebral cortex (Hoerder-Suabedissen and Molnár 2015), also appeared unaffected as Ctgf+ cells formed a uniform band along subplate in both mutants and controls at P10 (Fig. S6*C*). Examination of Nissl-stained sections along the rostro-caudal length of the neocortex of these dKOs was also performed, but no heterotopias, molecular ectopias or other cortical dysgenesis were detected (Fig. S7).

Given cortical migration is not restricted to the radial displacement of pyramidal neurons, we also investigated potential alterations to the distribution of tangentially migrating subpopulations of GABAergic interneurons (parvalbumin, somatostatin, calretinin) at P15 and Reelin+ cells at P10, encompassing both interneurons and Cajal-Retzius cells (Bielle et al. 2005; García-Moreno et al. 2008; Marín 2013). We found that these cell groups occupied the cortices of dKO brains in a similar fashion to control samples as they appeared equally distributed along the cortical wall (Fig. 3*O*–*Q*, Fig. S6*D*).

We also examined potential deficits in the laminar organization of cerebellum and hippocampus. Labeling subpopulations of hippocampal neurons with CB and Smi32 (Neurofilament-H), we found that hippocampi of *AU040320* and *Kiaa0319;AU040320* mutants displayed an overall normal distribution of cells (Fig. S8). In the cerebellum, CB+ Purkinje cells and granule neurons (NeuN+) appeared at their expected positions in both mutants (Fig. S9). Altogether, these results indicate that loss-of-function mutations in both *Kiaa0319* and *AU040320* do not lead to abnormalities in neuronal lamination in the mouse brain.

### Acute Knockout of *Kiaa0319* and *AU040320* During Development Does Not Alter Radial Migration in the Neocortex

The findings reported above are in disagreement with the previously reported association between KIAA0319 and KIAA0319L and cortical neuronal migration after shRNA knockdown in rats (Paracchini et al. 2006; Peschansky et al. 2010; Szalkowski et al. 2012; Adler et al. 2013; Platt et al. 2013). This approach disrupts protein function at the point when migration is taking place, not from developmental onset, therefore limiting the potential for regulatory compensation (Rossi et al. 2015). In order to mimic the developmental time-course of the original shRNA experiments, we performed in utero electroporation to deliver Cre recombinase to the developing cortex of double *Kiaa0319;AU040320* floxed embryos (dFlox) and acutely eliminate the proteins when neuronal migration is taking place. Experiments were performed at E14.5 to target upper layer neurons using plasmids to simultaneously express Cre and EGFP (pCIG-CRE-IRES-GFP; Fig. S6*E*) or simply EGFP as a control condition (pCIG-GFP). Brains were harvested at E18.5 to allow 4 days for cells to migrate. As expected, electroporated cells appeared predominantly in the upper cortical plate in both test and control brains (Fig. 3*R*). Quantification of GFP+ cells across different sectors of the developing cortex showed no significant differences (Fig. 3*S*). Combined with the results above, this finding indicates that KIAA0319 and AU040320 do not play an essential role in neuronal migration in the developing mouse neocortex.

### Impaired Silent Gap Detection in the Absence of Both KIAA0319 and AU040320

Altered levels of KIAA0319 protein in shRNA-treated rats have been reported to affect spatial memory and auditory processing (Szalkowski et al. 2012; Centanni et al. 2014a, 2014b), while *Kiaa0319* KO mice have only very mild deficits in anxiety-like behavior and sensorimotor gating (Martinez-Garay et al. 2017). To gain a better understanding of the contributions of KIAA0319 and AU040320 to normal behavioral functions, we conducted a series of behavioral tests in dKO mice.

**Figure 4.**
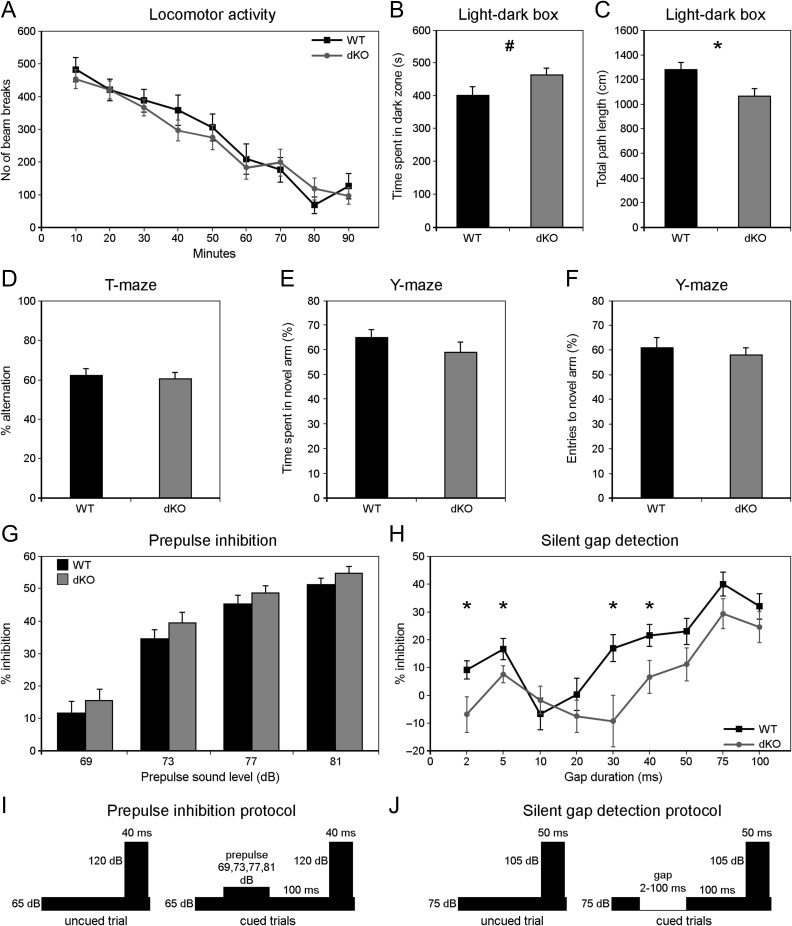
Double KO mice are impaired in a gap-in-noise detection task. (*A*) Locomotor activity test in a novel environment shows a similar number of beam breaks per 10 min for WT and double KO mice over the course of 90 min (*P* > 0.05). (*B*, *C*) Double KO mice display a suggestive increase in anxiety-like behavior by spending more time in the dark area in the light–dark box (*B*, *P* = 0.06) but that was accompanied also by a decrease in overall locomotor activity in the chamber (*C*, *P* = 0.01). (*D*–*F*) Spatial memory in dKO was unaffected as indicated by similar percentage of arm alternations in the T-maze when compared to WT controls (*D*), and similarly for exploration in the Y-maze indicated by time spent (*E*) and number of entries (*F*) in a novel arm. (*G*) No differences are detected in the percentage of startle inhibition displayed by WT and dKO mice when presented with stimuli as shown in *I*. (*H*) Double KO mice show lower inhibition of startle reflex in a silent gap detection task (see *J*), with significant differences at certain gap durations (2 ms, *P* = 0.03, *d *= 0.76; 5 ms, *P* = 0.04, *d* = 0.57; 30 ms, *P* = 0.01, *d* = 0.84; 40 ms, *P* = 0.04; *d *= 0.77). All data shown as means ± SEM (*n* = 18 per genotype). (*I*) Diagram of stimuli used in prepulse inhibition protocol; a startle-eliciting stimulus of 120 dB SPL and 40 ms duration was presented alone (uncued trial) or 100 ms after offset of a 20 ms prepulse of varying sound intensity levels above background noise (65 dB SPL) (cued trials) (full protocol diagram shown in Fig. S10*G*). (*J*) Diagram of stimuli used in silent gap detection protocol; a startle-eliciting stimulus of 105 dB SPL and 50 ms duration was presented alone (uncued trial) or 100 ms after offset of a silent gap of varying duration in the background noise (75 dB SPL) (cued trials) (full protocol diagram shown in Fig. S10*H*).

First, we assessed baseline locomotor activity following exposure to a novel environment and found normal levels of activity in mutants when compared to controls (Fig. 4*A*; *F* = 0.20, *P* = 0.65). In the light–dark box test, dKOs displayed a reduction in overall activity as shown by their decrease in distance traveled in the apparatus (Fig. 4*C*; *F* = 7, *P* = 0.01, *d* = 1.26). This was accompanied by a tendency to spend more time in the anxiolytic dark zone, although this difference did not reach statistically significant levels (Fig. 4*B*; *F* = 3.76, *P* = 0.06). Assessing motor coordination and learning in the accelerating rotarod test, the performance of dKO mice was comparable to that of wild-types (Fig. S10*A*). Similarly, we detected no alterations in dKO's spatial memory abilities when tested in the spontaneous alternation test in T-maze (Fig. 4*D*; *F* = 0.13, *P* = 0.72), with both groups alternating arms above chance levels (WT, *t*(17) = −3.72, *P* = 0.009; dKO, *t*(17) = −3.16, *P* = 0.01), and also in the Y-maze as mutants explored the novel arm similarly to controls (Fig. 4*E*,*F*; time of exploration, *F* = 1.85, *P* = 0.18; and number of entries, *F* = 0.55, *P* = 0.46). Acoustic startle reflex and prepulse inhibition (PPI) (protocol diagrams shown in Fig. 4*I* and Fig. S10*G*) were also similar for dKO animals and controls (Fig. S10*B* and Fig. 4*G*, respectively; for PPI, repeated-measures ANOVA with stimulus as a within-subject factor, *F* = 1.89, *P* = 0.17; or with genotype × stimulus as between-subject factors, *F* = 0.15, *P* = 0.93).

We then assessed dKOs in a gap-in-noise detection task (gap-PPI) (Fig. 4*J* and Fig. S10*H*) to test auditory temporal processing. For gap durations of 2–100 ms (with 0 ms gaps on the uncued trials), both dKO and WT groups displayed higher inhibition of startle with increasing duration of the gap cue, as expected given that longer gaps are more salient (Fig. 4*H*). However, at most gap durations, gap-inhibition of acoustic startle (% inhibition on gap-cued relative to uncued trials) was weaker for dKOs than WTs; that is, gap detection was poorer in dKOs. A repeated-measures ANOVA with genotype as a between-subject factor and gap duration as a within-subject factor indicated an effect of genotype on performance (*F* = 2.12, *P* = 0.03), and post hoc Tukey tests revealed significant differences in gap-PPI at several gap durations (Fig. 4*H*). Notably, since gap-PPI was abnormal in dKO animals at long as well as short gap durations, the results suggest a general deficit in gap-in-noise processing rather than a specific deficit in temporal acuity.

In order to dissociate the relative contributions of KIAA0319 and AU040320 to this phenotype, we subjected single KO cohorts with their respective wild-type littermates to a similar series of startle-based experiments. None of the mutant groups displayed any differences in startle reflex (data not shown), PPI (Fig. S10*C*,*D*), or gap detection (Fig. S10*E*,*F*). These results indicate that combined, but not individual, deletions of KIAA0319 and AU040320 can lead to impairment in the ability to discriminate subtle differences in auditory stimuli in the presence of noise.

### Impaired ABRs in dKO Mice and Specific ABR Wave III Deficit in *AU040320* KOs

A behavioral deficit in gap-PPI could arise from abnormalities in many different brain structures. Therefore, we decided to use ABR measurements to determine whether the absence of KIAA0319, AU040320, or both proteins affects the early stages of auditory processing.

**Figure 5.**
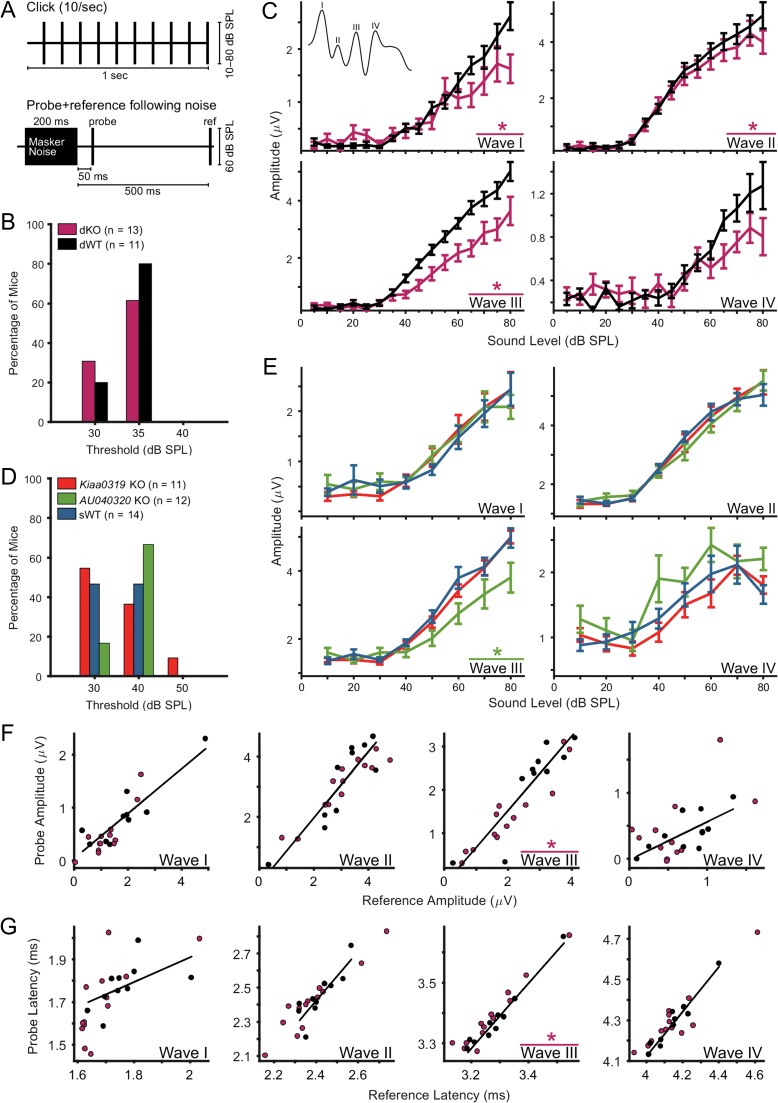
*AU040320* single and double knockout mice show suprathreshold alterations in ABRs. (*A*) Diagrams of stimuli for ABR recordings. Top, clicks presented at 10 clicks/s, used for analysis of click-evoked ABR thresholds, amplitudes and latencies (see *B*–*E*); bottom, masker noise followed by probe and reference clicks, used for analysis of effects of preceding noise on click-evoked ABRs (see *F*,*G*). (*B*, *D*) No differences in click-evoked ABR thresholds between KO and WT animals for any of the groups. (*C*) ABR wave amplitudes for dKO (*n* = 13) and dWT (*n* = 11) mice. Wave I and II amplitudes were significantly reduced (*) in dKO compared to dWT mice especially at higher sound levels (RM-ANOVA (group × sound level) wave I: *P* = 0.002; wave II *P* = 0.044). Wave III amplitude in dKO mice was significantly reduced regardless of sound level (RM-ANOVA (group) *P* = 0.014) (see Table S5). Color key as in (*B*). Error bars, mean ± SEM across animals. (*E*) ABR wave amplitudes for *Kiaa0319* (*n* = 11) or *AU040320* (*n* = 12) single KO mice and WT controls (sWT; *n* = 14). Wave III ABR wave amplitude was significantly reduced in *AU040320* KO compared to WT animals (RM-ANOVA (group) *P* = 0.029); no significant differences in ABR amplitude between *Kiaa0319* KO and WT mice for any of the 4 waves (see Table S5). Error bars, mean ± SEM across animals. Color key as in (*D*). (*F*, *G*) ABR wave amplitudes and latencies for dKO and dWT mice in response to a “probe” click 50 ms following the end of a 200 ms, 60 dB SPL noise versus a “reference” click 500 ms after the noise offset (see bottom diagram in *A*). Scatterplots compare amplitude (*F*) or latency (*G*) of ABR waves evoked by probe and reference clicks; solid lines indicate 2D least-mean-squares fits to the WT data. There was a significant difference between dKO and dWT mice in ABR wave III amplitude and latency for probe click relative to reference click (binomial test on number of points above and below the dWT best-fit line; wave III amplitude *P* = 0.003; wave III latency *P* = 0.0002). There were no other significant differences between dKO and dWT mice.

Experiments were conducted in 2 different cohorts of animals: 1) a group of 13 dKOs and 11 age-matched wild-type mice (dWTs), and 2) 2 groups of single KOs, 11 for *Kiaa0319* and 12 for *AU040320*, with 14 age-matched wild-type littermates pooled together as wild-type controls for both single KOs (sWTs). There were only minor effects of either gender or age on the ABR measures used for analysis of genotype effects (Figs S13 and S14). Moreover, there were no significant differences in click ABR thresholds between mutants and controls (Fig. 5*B*,*D*; Wilcoxon rank-sum test, all *P* > 0.1), suggesting that hearing sensitivity is not impaired by absence of these proteins.

To test for suprathreshold deficits in auditory processing, we examined ABR waveforms evoked by clicks at 50–80 dB SPL sound levels, well above the click ABR threshold for all individual animals. In dKO mice, amplitudes of click ABR waves I–III were significantly smaller than in dWT mice (Fig. 5*C*; RM-ANOVA: wave I (group × sound level) *P* = 0.002, *F* = 5.536; wave II (group × sound level) *P* = 0.044, *F* = 2.855; wave III (group) *P* = 0.014, *F* = 7.132; Table S5). The wave III amplitude difference was the most reliable of these results as it was evident in post hoc Tukey tests at each sound level tested (Table S5). These results indicate that absence of both AU040320 and KIAA0319 disrupts suprathreshold auditory processing in the brainstem and/or the periphery.

In single KO animals, suprathreshold ABR abnormalities were apparent only in *AU040320* KO mice and primarily for ABR wave III, where the amplitude was significantly reduced in *AU040320* KOs compared to sWTs (Fig. 5*E*; RM-ANOVA (group) *P* = 0.029, *F* = 5.405; Table S5, post hoc Tukey tests *P* < 0.05 at all but one sound level). In contrast, in *Kiaa0319* KO mice no abnormalities were detected (Fig. 5*E* and Table S5). These findings suggest that absence of AU040320 selectively impairs auditory processing within the brainstem.

Mutations affecting action potential timing can prolong ABR wave latencies (Kopp-Scheinpflug and Tempel 2015). Therefore, we also analyzed ABR wave latencies. However, we found no significant differences between dKO or *Kiaa0319* KO mutants and their respective controls (Fig. S11). In *AU040320* KO mice, an RM-ANOVA (group × sound level) revealed a significant difference from sWT mice in click ABR wave II latency; however this effect was not significant at individual sound levels in post hoc Tukey tests (Table S6). Thus, click ABR wave latencies appeared to be largely unaffected by the absence of AU040320, KIAA0319, or both proteins. We also tested for possible changes in ABR wave “jitter” by calculating the standard deviation in wave latency across repeated trials, and found no significant differences between any of the mutant and control mice (data not shown). We conclude that abnormalities in click ABR waves I–III in dKO mice, and wave III in *AU040320* KO mice, are specific to wave amplitudes and wave latencies are largely unaffected.

Previous studies in humans have suggested that dyslexic subjects may have deficits in short-term memory mechanisms underlying auditory adaptation to repeated or prolonged stimuli (Ahissar et al. 2006; Perrachione et al. 2016). To investigate whether the selective deficit in click ABR wave III amplitude in *AU040320* KO mice might reflect an abnormality in the time-course for adaptation induced by repeated clicks, we repeated our measurements of click ABR waves in single KO animals using a slower click rate (2 instead of 10 clicks/s). Wave III amplitude was still significantly reduced in *AU040320* mutants relative to controls (Fig. S12*A*; RM-ANOVA (group) *P* = 0.005, *F* = 9.347; result confirmed by post hoc Tukey tests at all sound levels, Table S7). At this slower click rate, we also found significant differences between mutant and control animals for click ABR wave II amplitude in *Kiaa0319* KO and for click ABR wave II and III latencies in *AU040320* KO mice (Fig. S12 and Table S7); however, these effects were not significant at individual sound levels in post hoc Tukey tests. Thus, click ABR wave III amplitude is still significantly reduced in *AU040320* KO relative to control mice even when the clicks are separated by 500 ms, indicating that the abnormality is unlikely to arise from changes in adaptation to repeated clicks.

### Subtle Abnormalities in Auditory Brainstem Recovery from Adaptation to Noise in dKO Mice

Repeated clicks, even at the faster rate of 10 clicks/s, would not be expected to elicit very strong auditory adaptation. To explore the possibility of deficits in auditory adaptation using a stimulus that drives more robust brainstem adaptation, we asked whether additional abnormalities in click ABR measures might be revealed in mutant mice if clicks were presented following a masking noise. We used a 60 dB SPL “masker-probe-reference” stimulus: a 200 ms broadband noise masker, followed by a probe click 20 or 50 ms after masker offset and a reference click 500 ms after masker offset (Fig. 5*A*). We examined the relationship between probe and reference click ABR measures to determine how brainstem responses to clicks were affected by preceding noise in mutant versus control animals.

In dKO mice (Fig. 5*F*,*G*), these analyses revealed additional, context-dependent, abnormalities in ABR wave III. For probe clicks presented 50 ms after the end of the masker noise, the relationship between probe click and reference click ABR amplitudes and latencies differed significantly between dKO and dWT mice; wave III amplitudes were more reduced and latencies more prolonged when preceded by noise in mutant than in control animals (Fig. 5*F*,*G*; binomial test for asymmetry relative to WT best-fit line, wave III amplitude *P* = 0.003, latency *P* < 0.001; Wilcoxon rank-sum test on probe/reference ratios, wave III amplitude *P* = 0.002, latency *P* = 0.013). When the probe delay was shortened to 20 ms, similar abnormalities also restricted to later ABR waves were detected in dKO mice (Fig. S15*A*,*B*). Additional experiments using 20 ms probe delays revealed potential abnormalities in *AU040320* KOs as the relationship between probe and reference click in the amplitude of ABR wave II differed from controls (Fig. S15*C*,*D*). These results suggest that absence of both AU040320 and KIAA0319, and possibly absence of AU040320 alone, affect the rate of auditory brainstem recovery from adaptation to noise.

## Discussion

Several studies using shRNA knockdown in rats have implicated the rodent homologs of *KIAA0319* and *KIAA0319L* in neuronal migration (Paracchini et al. 2006; Peschansky et al. 2010; Szalkowski et al. 2012; Adler et al. 2013; Platt et al. 2013). However, manipulating protein levels genetically in mice did not affect the lamination or migration of cortical, hippocampal or cerebellar neurons (Martinez-Garay et al. 2017). To determine whether functional overlap of KIAA0319 and KIAA0319L could explain this apparent discrepancy, we examined the development of the neocortex in mice lacking AU040320 alone or in conjunction with KIAA0319. We found no evidence in support of a role of these 2 proteins in neuronal migration as dKOs (and *AU040320* KOs) displayed normal distribution of projection neurons and tangentially migrating neuronal populations in the mouse neocortex. Moreover, acute knockout with in utero electroporation of CRE in double floxed mice did not lead to an arrest in radial migration in the embryonic brain. In addition, cortical neurogenesis and the morphology of other laminated brain regions, hippocampus and cerebellum, were also unaffected. However, we identified an impairment in a silent gap detection task in dKOs indicative of a deficit in the auditory system, which was confirmed with ABR recordings. Absence of AU040320 alone or together with KIAA0319 led to reduced ABR wave III amplitudes, with waves I and II also affected in the dKOs. Further investigation of ABRs to clicks following noise in dKO animals confirmed that abnormalities were particularly pronounced for ABR wave III. It is possible that subtle genetic and/or epigenetic differences could have a contribution to the results from the dKO experiments given the dWT controls were not actual littermates although, as described in Materials and Methods section for the generation of the dWT cohort, strong precautions were taken to minimize potential external factors. Nevertheless, these results indicate a role for KIAA0319 and especially AU040320 in the normal development and/or function of auditory brainstem structures, and support the hypothesis that dyslexia susceptibility genes might produce specific abnormalities in central auditory processing.

### Discrepancy with shRNA Experiments

Our results are in stark contrast to reports linking KIAA0319 and KIAA0319L with neuronal migration mentioned above. Whilst rat-mouse species differences could potentially account for the discrepancies, mismatches between RNA interference (RNAi) and genetic deletions are well-known in the literature, including in the context of dyslexia susceptibility genes. Of the 4 main dylexia susceptibility candidate genes, only *Robo1* has been linked to neuronal migration in studies using both RNAi and genetic approaches (Gonda et al. 2013). For both *Dcdc2* and *Dyx1c1*, loss-of-function mutations in mice produced no neuronal migration abnormalities (Wang et al. 2011; Rendall et al. 2015) in contrast to results of knockdown experiments in rats (Meng et al. 2005; Wang et al. 2006; Burbridge et al. 2008; Adler et al. 2013).

Robustness against perturbations such as null mutations is a key property of biological systems (Kitano 2004). Accordingly, a recent study has shown compensatory gene circuits are more likely to be activated in genetic knockouts than in knockdown approaches where protein function is disrupted acutely (Rossi et al. 2015), possibly explaining the discrepancy observed for dyslexia susceptibility genes. The strategy of expressing Cre recombinase in floxed mice after in utero electroporation aims to circumvent these potential compensatory mechanisms and basically recapitulates the same developmental conditions of the shRNA knockdown experiments performed in rats for *Kiaa0319* and *Kiaa0319-Like*/*AU040320*. Nonetheless, our electroporations simultaneously targeting *Kiaa0319* and *AU040320* at the genetic level in floxed mice did not lead to observable defects in neuronal migration. Similar results have been obtained with *Dcdc2* floxed mice (Wang et al. 2011).

The differences observed in the literature are likely to derive from shRNA off-target effects, a constant source of concern and investigation over the years (see e.g., Jackson and Linsley 2010; Bofil-De Ros and Gu 2016). For example, Baek et al. (2014) has found that knockdown of *Dcx* led to migration problems that were indistinguishable when performed in wild-types and *Dcx* KO mice, where no functional *Dcx* mRNA is present. Similar results have been obtained for *Disc1* where knockdown-knockout discrepancies have been noted (Kvajo et al. 2012; Tsuboi et al. 2015). Several other reports have been published with similar effects following the use of shRNA (Alvarez et al. 2006; McBride et al. 2008).

What does this mean for our understanding of the functional genetics and neurobiology of dyslexia? Considering the shortcomings of shRNA methods and the lack of replication of knockdown-induced neuronal migration abnormalities in knockouts for multiple dyslexia candidate genes, the view that dyslexia susceptibility genes play a role in neuronal migration should be carefully re-evaluated, and the existing literature considered with the caution the situation requires. This is particularly important in the current climate of growing concern over reproducibility in scientific research (Bustin 2014; Open Science Collaboration 2015; Munafò et al. 2017).

### Behavioral Impairments in Double KO Mice

The observed impairment in gap detection in dKO mice is in line with the fact that deficits in auditory processing have been repeatedly, albeit controversially, linked with dyslexia (Tallal 1980; Ramus 2003; Rosen 2003; Giraud and Ramus 2013; Goswami 2014; Peterson and Pennington 2015). The gap-inhibition of acoustic startle paradigm is commonly used to probe temporal acuity in auditory processing (Fitch et al. 2008, 2013), and it has been argued that a deficit in temporal processing is one of the key mechanisms underlying auditory-based deficits in dyslexia (Tallal 1980; Temple et al. 2000; Choudhury et al. 2007; Raschle et al. 2014; de Groot et al. 2015; but see also Breier et al. 2003; Georgiou et al. 2010; Protopapas 2014). Accordingly, other genes associated with language disorders such as *DCDC2* and *CNTNAP2* have been linked to impaired temporal auditory processing in mouse models of gene function using this paradigm (Truong et al. 2014, 2015), with similar findings reported for rats treated with shRNA against *Dyx1c1* (Threlkeld et al. 2007; Szalkowski et al. 2013), although this has not been confirmed in KO mice (Rendall et al. 2015).

With specific reference to *Kiaa0319*, in utero knockdown in the developing rat neocortex was reported to lead to subtle deficits in detection of silent gaps of very brief duration (up to 10 ms) as well as in responses to more complex stimuli such as frequency modulated sweeps (Szalkowski et al. 2012). The experiments reported here identified a gap detection deficit extending across a wider range of gap durations following manipulation of both *Kiaa0319* and *AU040320* genes, but not following deletions of either gene alone. These results are consistent with our finding that auditory brainstem recovery from adaptation to noise is abnormal in dKO mice.

The *Kiaa0319-*shRNA-treated animals were also found to suffer from deficits in spatial memory when tested in the Morris water maze (Szalkowski et al. 2012). Whilst this specific test was not conducted with dKOs, testing in the T- and Y-maze did not provide indications of impairment in spatial memory, consistent with our previous observations in *Kiaa0319* KO mice (Martinez-Garay et al. 2017). However, it is important to evaluate these distinctions with care given the potential issues with specificity of shRNA approaches outlined above.

### Implications of Observed ABR Abnormalities

Waves I–IV of the ABR in mice are thought to arise from volleys of synchronous neural activity in the ascending auditory pathway, and are typically attributed to the auditory nerve (wave I), cochlear nucleus (wave II), trapezoid body and/or superior olivary nuclei (wave III), and lateral lemniscus and/or inferior colliculus (wave IV) (Henry 1979; see also Willott 2001; Jalabi et al. 2013). In both dKO and *AU040320* KO mice, ABR abnormalities were most pronounced for the late ABR wave III suggesting that absence of AU040320 may disrupt auditory processing primarily in central rather than peripheral auditory structures, and particularly the trapezoid body and superior olivary nuclei (Jalabi et al. 2013). The ABR data also suggest that absence of both AU040320 and KIAA0319, and possibly absence of AU040320 alone, alters auditory brainstem recovery from adaptation to noise.

There are intriguing parallels between these results and previous findings from studies of adaptive sensory processing in humans with dyslexia. In both the visual and the auditory domains, dyslexics have been reported to perform more poorly than controls in tasks involving perception in noise (Sperling et al. 2005; Ziegler et al. 2009), suggesting that difficulty in excluding perceptual noise may be a core deficit in dyslexia (but see Calcus et al. 2017). Other studies in individuals with dyslexia have demonstrated dysfunction in rapid repetition-induced adaptation in sensory cortical areas (Perrachione et al. 2016), and deficits in auditory perceptual adaptation to longer-term stimulus regularities (Ahissar et al. 2006; Jaffe-Dax et al. 2017). Taken together, these studies suggest that adaptive mechanisms underlying estimation of statistical regularities in sensory stimuli may be impaired in dyslexic subjects. Abnormal ABRs to clicks following noise in dKO mice (and *AU040320* KO mice) could be interpreted as evidence for a more basic form of this deficit in the mutant mice, affecting the rate of neural adaptation to recent changes in noise level. Many different cellular and synaptic changes in brainstem neurons could alter the rate of neural adaptation to noise; thus, intracellular recordings in the auditory brainstem generators of ABR wave III in *AU040320* KOs may be required to determine the mechanism underlying the observed abnormality.

The mechanisms underlying abnormal adaptation to stimulus changes might also involve higher auditory areas including the cortex. In the auditory system, there are extensive descending connections from cortical to subcortical structures (Winer 2006; Luo et al. 2008), as well as olivocochlear projections from the superior olivary nuclei into the sensory transduction machinery of the cochlea (Guinan 2006). These efferent pathways are thought to contribute to optimization of auditory processing in noisy environments (Guinan 2006; Luo et al. 2008). Previous reports of speech-evoked auditory brainstem abnormalities in individuals with dyslexia (Chandrasekaran et al. 2009; Hornickel and Kraus 2013; White-Schwoch et al. 2015), including a recent study demonstrating an association between ABR abnormalities and dyslexia risk loading for *KIAA0319* alleles (Neef et al. 2017), have often emphasized the potential for brainstem abnormalities to arise from abnormalities in top-down modulation. Further experiments will be necessary to determine exactly how the absence of AU040320 and/or KIAA0319 proteins affects cellular properties, synaptic transmission, neural circuitry, and both afferent and efferent connectivity in the auditory brainstem. For example, we have recently reported that KIAA0319 plays a role in regulating axon growth (Franquinho et al. 2017) which can directly impact on projection patterns in the brain. In addition, since previous experiments on *Kiaa0319*-shRNA-treated rats have reported abnormal firing patterns in auditory cortical neurons (Centanni et al. 2014a), further investigations in *Kiaa0319* KO mice are needed to determine whether auditory abnormalities exist higher in the auditory pathway, to address the possibility that the more pronounced auditory brainstem deficits in dKO than in *AU040320* KO mice arise from a specific impairment in efferent input to the brainstem in the absence of KIAA0319.

## Supplementary Material

Supplementary Data
