# Supplementary material for: Knockout Mice for Dyslexia Susceptibility Gene Homologs KIAA0319 and KIAA0319L have Unaffected Neuronal Migration but Display Abnormal Auditory Processing
Source: Cereb Cortex. 2017 Oct 17;27(12):5831–45. doi: 10.1093/cercor/bhx269 (PMC5939205; doi:10.1093/cercor/bhx269)
Supplement: Supplementary Data [file bhx269_supplement.zip › Guidi_SuppInfo.pdf]

Knockout mice for dyslexia susceptibility gene homologs *KIAA0319* and *KIAA0319L* have unaffected neuronal migration but display abnormal auditory processing

Luiz G. Guidi[1,2], Jane Mattley[3], Isabel Martinez-Garay[1,5], Anthony P. Monaco[2,6], Jennifer F. Linden[3,4], Antonio Velayos-Baeza*[2], Zoltán Molnár*[1]

SUPPLEMENTARY INFORMATION

Supplementary Materials and Methods

Generation of *AU040320*-targeted mice

Mouse JM8A3.N1 embryonic stem cells (Pettitt et al. 2009) targeted at the *KIAA0319L*-homologous mouse gene *AU040320* with a "knockout-first" (KO1, reporter-tagged insertion with conditional potential) allele (C57BL/6N-*AU040320*$^{tm1a(EUCOMM)Wtsi}$) (Skarnes et al. 2011) had been generated under the International Knockout Mouse Consortium project ID 27003. Information about gene organisation and details of the targeting strategy around exon 3 are provided in Fig. S1. Two clones (EPD0726_2_H05 and EPD0726_2_F06) passing the relevant PCR and chromosome counting quality control tests were obtained from the European Conditional Mouse Mutagenesis Program (EUCOMM, www.eucomm.org) and used for C57BL/6J blastocyst injections at the Transgenics Core of the Wellcome Trust Centre for Human Genetics in Oxford (UK). Three male chimeras were obtained from *AU040320-KO1* ES cell clone H05 (~ 75%, 30% and 10% contribution from the injected ES cells) and two with clone F06 (~ 95% and 80% contribution). Germline transmission, checked for all these mice (except the 10% chimera) after breeding with C57BL/6J

females by the presence of F1 pups with agouti coat colour and/or positive for KO1-specific genotyping PCR, was successful for the F06-derived chimeras, although only mice derived from the 95% chimera were positive for all specific genotyping PCRs. All *AU040320*-targeted mice used in this work were derived from the F06-95% chimera in a similar way as described for the *Kiaa0319*-targeted mice (Martinez-Garay et al. 2017) to obtain *AU040320-KO1* (C57BL/6J-*AU040320*$^{tm1a(EUCOMM)Wtsi}$), *-del* (or *NZ*) (C57BL/6J-*AU040320*$^{tm1b(EUCOMM)Wtsi}$), *-Flx* (C57BL/6J-*AU040320*$^{tm1c(EUCOMM)Wtsi}$) and *-Null* (C57BL/6J-*AU040320*$^{tm1d(EUCOMM)Wtsi}$) colonies (Fig. 1A). Identification of *AU040320* alleles was done using a number of genotyping PCRs (Fig. 1A, C; Table S2). Integrity of the *KO1* allele was confirmed by overlapping PCR and sequencing expanding ~16.6 kb between the flanking regions of the homology arms used in the targeting cassette (Table S2, Fig. S2). Similarly, *del* and *Flx* alleles were checked by sequencing of small fragments expanding the *loxP* and *FRT* recombination points, respectively (Table S2). The predicted result at the protein level in *KO1* and *del* alleles is a very short chimeric protein (p.D48GfsX102) due to disruption by the trapping cassette of normal splicing after exon 2, while the *Null* allele would lead to p.D48GfsX14 (or no protein if the resulting transcript undergoes degradation by nonsense-mediated mRNA decay (NMD)). Putative alternative splicing by skipping of the splicing acceptor site in the trapping cassette in the *KO1* allele would lead to normal protein while in the *del* allele it would have a *Null*-allele effect.

Behavioural testing

<u>Locomotor activity</u>: to measure locomotor activity in a novel environment, mice were placed in Plexiglass cages (20 × 35 cm) equipped with infrared photobeams (San Diego Instruments) over a total of 90 min. Testing always took place in the light phase and started at 10:00 h for all groups. Activity was measured as number of beam breaks and divided into 10 min bins for analysis.

Light/dark box: testing was performed using Med Associates activity chambers (ENV-510) with a Perspex dark box insert covering half the area of the chamber (27 x 13.9 x 21.5 cm) and a 4 x 4 cm hole placed in the middle at floor level to divide it into light (300 lux) and dark (2 lux) compartments. Animals were started in the light compartment and each session lasted 10 min. Performance was automatically scored by Med Associates activity software and measured time spent in each zone, distance travelled, latency to enter dark zone and the number of transitions between zones.

Accelerating rotarod: motor coordination and learning was assessed using an accelerating rotarod (Model 7650, UgoBasile). Mice were placed on a rod moving at 4 rpm that began to accelerate after 10 s at a rate of 20 rpm/min. Latency of animal to fall from the rod was recorded. Mice were given three trials per day over the course of three consecutive days, with a minimum of 60 min inter-trial interval. A habituation phase took place for 30 s one day before testing. Failure to grip in adaptation phase or in initial 10 s of experiment counted as disqualification but all animals passed the test.

Olfactory preference task: in order to assess responses to distinct olfactory stimuli, perception of aversive and attractive smells was assessed based on protocol described in (Witt et al. 2009).

Y-maze (spatial novelty): spatial novelty was assessed using a Perspex Y-maze (30 × 8 × 20 cm arm dimensions). The base arm of the maze was defined as the “start” arm. In the sampling phase, animals were assigned one of the other two arms (the “other” arm) counterbalanced for genotype and allowed to explore both start and other arms for 5 min. The animal was then returned to its home cage for 1 min before being placed back in the start arm for a 2 min exploration with access to all three arms. Time spent in each arm, distance travelled and number of arm entries were recorded automatically using a video camera fixed to the ceiling coupled to tracking software AnyMaze (Stoelting).

Spontaneous alternations (T-maze): spatial working memory was assessed using a black

wooden T-maze (30 x 10 cm, walls 29 cm high). Mice were placed in the start arm and allowed to choose a goal arm. A central partition wall was placed at the end of the start arm which extended the decision-making point 7 cm into the start arm and blocked any experience of the non-visited arms. Once the animal entered the goal arm, a guillotine door was used to confine the animal to it for 30 s for exploration. Arm chosen and latency to enter goal arm were recorded. The animal was then removed from the apparatus for 15 s, guillotine and central partition were removed and the animal was placed back in the start arm for free exploration in a "choice" phase. The arm chosen on the second run and latency to enter it were recorded before returning the mouse to its home cage. Animals were tested twice a day (morning and afternoon) for 5 consecutive days. Spatial memory was assessed using percentage of arm alterations – i.e. arm chosen in choice phase was different from the one chosen at exploration.

Startle reflex and prepulse inhibition of acoustic startle: an SR-Lab System (San Diego Instruments) was used to measure startle responses and pre-pulse inhibition of acoustic startle. A diagram of this protocol is shown in Fig. S10G, with a simplified representation of stimuli also in Fig. 4I. Mice were placed in a Plexiglass cylinder for 5 min exposed to broadband white noise background at 65 dB SPL followed by a habituation phase of 5 x 40 ms 120 dB SPL stimuli separated by 15 s. Test sessions contained a randomised set of the following stimuli: a stimulus consisting of only background noise (65 dB SPL) for baseline movement measurements; a 40 ms, 120 dB startle-eliciting noise "pulse" stimulus; 4 x 20 ms "prepulse" broadband noise stimuli presented at 4, 8, 12 or 16 dB SPL above background noise; and, finally, 4 combinations of prepulse-plus-pulse stimuli with a 100 ms silent delay between offset of the prepulse sequence and onset of the startle-eliciting pulse. Six blocks of trials were presented, with each stimulus once in each block. Intervals between each trial type varied between 10, 15 and 20 s. Five 40 ms, 120 dB SPL stimuli were presented at the end of the session. Responses to stimuli were recorded using

average startle amplitude for every millisecond during a 65 ms sampling window starting at the onset of each startle-eliciting pulse. Prepulse inhibition was calculated from the startle ratio (average startle amplitude on cued trials normalised by average startle amplitude on uncued trials), and is presented as % inhibition = 100 * (1 – startle ratio).

Silent gap detection: to assess the effect of genetic mutations on rapid auditory processing, a silent gap startle paradigm was used as previously described (Fitch et al. 2008; Truong et al. 2014). The protocol is identical to the prepulse inhibition design described above with exception of the stimuli, which consist of continuous background noise at 75 dB SPL interspersed with silent gaps instead of prepulses. On cued trials, silent gaps varying in duration between 2-100 ms (2, 5, 10, 20, 30, 40, 50, 75, 100 ms) interrupted the background white noise. The offset of each silent gap was followed by 100 ms of background white noise before presentation of the startle pulse (105 dB SPL, 50 ms). Stimuli with silent gaps of 0 ms duration were used as uncued, control trials. Presentations of cued or uncued trials were randomised, with inter-trial intervals of 10, 15 or 20 s between startle pulses on successive trials. A diagram of this protocol is shown in Fig. S10H, with a simplified representation of stimuli also in Fig. 4J. Like prepulse inhibition of acoustic startle, gap-inhibition of acoustic startle was quantified using the startle ratio (average startle amplitude on cued relative to uncued trials), and data is shown as % inhibition = 100 * (1 – startle ratio).

Auditory brainstem response (ABR) measurements

Briefly, subdermal needle electrodes (Rochester Medical) were placed at the vertex of the skull (+), on the tragus of the ear oriented toward the speaker (-), and on the tragus of the contralateral ear (ground). The speaker was positioned 17-18 cm from the ipsilateral ear; the contralateral ear was shielded by the head but not otherwise blocked. Mice were anaesthetised using a mixture of ketamine (100 mg/kg) and medetomidine (0.83 mg/kg)

administered intraperitoneally, followed by supplemental doses of ketamine as required. Anaesthesia level was evaluated at least every 30-60 minutes by monitoring changes in breathing rate or temperature and testing for a response to toe pinch. Atropine (up to 0.2 mg/kg) was given subcutaneously as required to reduce bronchial secretions, and Ringer's solution (0.1 ml) was administered subcutaneously approximately every hour to maintain hydration. A homeothermic blanket (Harvard Apparatus) was used to maintain body temperature at 37.5 ±0.5°C during the procedure.

An isolated sound booth was used for testing (Industrial Acoustics Company, Inc.). Auditory stimuli were presented from a free-field speaker (Tucker-Davis Technologies FF1) and calibrated before each experiment using a G.R.A.S. 40BF 1/4” microphone placed at the location of the ear to be tested, to ensure a flat speaker frequency response within ±2 dB spanning 2-80kHz. Stimuli were generated and data recorded using Tucker-Davis Technologies RX6 and RX5 signal processors, PA5 attenuator, SA1 speaker amplifier, RA4LI low-impedance headstage (20x gain) and RA16SD signal amplifier with bandpass filtering from 2.2 Hz to 7.5 kHz (and 20-sample, 800 μs group delay). Recordings were acquired at 24.414 kHz sample rate and then bandpass filtered 100-3000 Hz in software (5th order Butterworth filter).

All ABR data were analysed using custom Matlab software to determine ABR thresholds, wave amplitudes and wave latencies. ABR threshold was defined as the lowest sound intensity at which at least two clear wave deflections exceeding background variability (±SEM across repeated trials) could be identified by an observer blind to animal genotype. Wave amplitude was defined as the height of the wave peak relative to its following trough, and wave latency was defined as the time point of the wave peak relative to click onset. All wave peak and trough determinations were performed blind to the genotype of the animal, and estimated on three independent occasions to assess reliability of the measurements. The final wave amplitudes and latencies were calculated using the mean estimates of

wave peak and trough locations across these three repeated measurements. Only waves I-IV were analysed, as wave V was not found to be reliably distinguishable in all mice.

ABR wave amplitudes and latencies were analysed using Repeated Measures Analysis of Variance (RM-ANOVA) with either genotype as a between-subjects measure and sound level as a repeated, within-subject measure, or genotype alone as a between-subjects measure. RM-ANOVA tests were performed on ABR data obtained using 50-80 dB SPL clicks, to ensure the analysis was applied to suprathreshold responses only. Significant results in RM-ANOVA were followed by *post-hoc* Tukey comparisons. To analyse effects of genotype on the relationship between ABRs evoked by probe and reference clicks (following a noise masker), probe click ABR wave amplitude (or latency) was plotted against reference click ABR wave amplitude (or latency), the two-dimensional least-mean-squares best-fit line was determined for data from WT animals, and any significant deviations from this relationship in KO mice were quantified by comparing the number of KO data points above and below the WT best-fit line using binomial tests. In addition, ratios of probe to reference click ABR wave amplitude (or latency) were compared between WT and KO mice using the non-parametric Wilcoxon rank-sum test. For all analyses, significance threshold was $\alpha=0.05$.

Supplementary References

Pettitt SJ, Liang Q, Rairdan XY, Moran JL, Prosser HM, Beier DR, Lloyd KC, Bradley A, Skarnes WC. 2009. Agouti C57BL/6N embryonic stem cells for mouse genetic resources. Nat Methods. 6:493-495.

Witt RM, Galligan MM, Despinoy JR, Segal R. 2009. Olfactory behavioral testing in the adult mouse. J Vis Exp. 949.

Supplementary Figure Legends

Figure S1. Structure of mouse *AU040320* gene and generation of "knock-out first" targeted allele. Details of gene organisation and its main transcript, obtained from Ensembl (www.ensembl.org). Exon 3 is targeted with a "knock-out first" (KO1) cassette (EUCOMM project ID: 27003) as detailed on the right side and in Fig. S2.

Figure S2. *AU040320* knockout-first allele. The DNA sequence of the "knockout first" (KO1) allele for *AU040320* gene, with the targeted exon 3 plus 5 kb of each intronic flanking region, is included. The different components and elements of the targeting cassette (*boxed names*) and primers used for genotyping and PCR amplification (see Fig. 1 and Table S2) are shown. Forward primers are underlined and named on the left side; reverse primers are in bold and named on the right side. Only 1 difference was found with the expected sequence after sequencing: an extra A (*yellow highlighted*) at the IRES element.

Figure S3. Multiple tissue protein expression and histology analysis in wild-type and *AU040320* KO samples. (A) Western blot of multiple mouse tissues to detect AU040320 protein in wild-type (+/+) and *AU040320* -/- animals shows the protein is present throughout the mouse body at ~150 kDa (indicated by arrow). KO animals display the expected absence of the target protein. The custom-made antibody used here also detects a number of unspecific bands, both in +/+ and in -/- lysates. (B-D) Nissl-stained coronal and sagital sections of *AU040320* +/+ and -/- brains at P10 shows absence of AU040320 does not lead to gross morphological abnormalities in the brain. Lamination of the neocortex appears unaffected with barrel fields also clearly visible (C). Scale bars: 1000 µm (B), 50 µm (C), 2000 µm (D).

Figure S4. Normal cell division profiles in the developing cortex of *AU040320* KO and dKO embryos. (A, B) DAPI panel shows region selected for analysis in E15 (A) and E18 (B) cortices of WT and *AU040320* mutants by labelling cycling cells with an antibody against ki67 (red) and cells in M-phase with pH3 (green), with graphs of quantification of cell numbers indicating no differences

within the VZ or the rest of the cortical wall (non-VZ) in all conditions ($p>0.05$, $n=3$). (C) Panels show images used for a similar analysis of cell division programme in E18 dKO brains and controls, which displayed comparable number of pH3+ and ki67+ cells in each compartment ($p>0.05$, $n=3$). Data shown as mean ±SEM. CTX, cortex; LV, lateral ventricle; GE, ganglionic eminences; STR, striatum; cp, cortical plate; iz, intermediate zone; svz, subventricular zone; vz, ventricular zone. Scale bars: 400 µm (leftmost panels), 50 µm (insets).

Figure S5. Radial glial and intermediate progenitors are unaffected in the absence of AU040320 alone or in double KOs. DAPI images (leftmost panels) show regions selected for analysis at E15 and E18 and graphs show quantification of cell number (rightmost panels). Middle panels display representative images of the cortices of wild-type (WT), heterozygous (+/-), and homozygous (-/-) *AU040320* KO animals at E15 (A, B) and E18 (C, D), or in double KOs at E18 (E, F). In all mutants, Pax6+ radial glial cells cluster within the VZ (A, C, E) whilst Tbr2+ intermediate progenitors occupy a band immediately above it (B, D, F). Quantification of cell number dividing cortical wall into ten equal bins spanning 100 µm width is shown for all ages and cell populations and demonstrate no differences in any of the conditions examined ($n=3$, $p>0.05$). Data shown as means ±SEM. CTX, cortex; LV, lateral ventricle; GE, ganglionic eminences; STR, striatum; cp, cortical plate; iz, intermediate zone; svz, subventricular zone; vz, ventricular zone. Scale bars: 400 µm (leftmost panels); 100 µm (middle panels).

Figure S6. Normal distribution of neurons in the developing and postnatal cortex of single *AU040320* and in double KO animals. (A, B) Leftmost DAPI images show region of somatosensory cortex selected from E15 and E18 brains for analyses of cortical lamination of *AU040320* single and double KOs and magnified panels display distribution of Ctip2+ and Cux1+ cells in the brains of *AU040320*-KO at E15 and E18 (A) and in dKOs at E18 (B). Cux1+ cells (red) equally occupy the upper cortical plate (ucp) whilst Ctip2+ cells (green) are seen in the lower cortical plate (lcp) in E18 brains in wild-type and *AU040320* mutants (A, top panel). At E15, Ctip2+ cells (green) are seen forming the lower cortical layers of the emerging cortical plate (cp) (A, bottom panel). For dKOs (B), Cux1+ and Ctip2+ cells at E18 display the same pattern of cell

distribution as the wild-type control brains. Graphs represent percentage of positive cells in each of the ten bins spanning 100 µm along the cortical wall, with no differences detected in any of the conditions studied ($p>0.05$, $n=30$). Data shown as means ±SEM. (C) Ctgf immunohistochemistry (red) shows that neurons (NeuN+ cells, green) in the subplate layer in P10 dKO mice persist with a similar organisation when compared to wild-types. Images also reveal the presence of barrel fields in layer IV as expected. (D) Reelin immunostaining in P10 sections of wild-type and dKO mice shows similar distribution of Cajal-Retzius cells (green) in both conditions. (E) Cre immunostaining confirms expression of Cre recombinase by *in utero* electroporation in double floxed embryonic brains (see Fig. 3R). Scale bars: 1000 µm (leftmost DAPI panels in A, B), 75 µm (insets in A, B); 150 µm (C, D); 25 µm (E).

Figure S7. Double KOs exhibit normal overall brain anatomy and no cortical ectopias. Coronal sections of P10 brains stained with Cresyl violet for wild-type (WT) and *Kiaa0319;AU040320* double KO (dKO) brains. Representative images of serial sections (every third section at 50 µm thickness) used for examination of presence of cortical ectopias ($n=3$ per genotype). Scale bar: 1000 µm.

Figure S8. Normal hippocampal morphology in *AU040320* and double KO mice. Staining of Neurofilament H protein (Smi32, green) and calbindin+ cells (CB, red) shows overall organisation of developing and mature hippocampus in all mutants. For *AU040320* KOs (A-F), at P2, Smi32+ cells are seen migrating through CA3 (B) and subiculum (C). These cells later settle in CA3 at P10 to project across CB+ neurons (E) and show sparse distribution in CA1 (F). CB+ cells are seen to distribute across hippocampal layers in CA1 at P10 (F) in wild-types and *AU040320* mutants. In P10 dKO mice (G-I), Neurofilament H+ cells (Smi32, green) occupy CA3 to project across CB+ cells (CB, red; H), which are seen to delineate the layers around CA1 (I): stratum oriens (so), stratum pyramidale (sp) and stratum lucidum (sl). DG, dentate gyrus; SB, subiculum; so, stratum oriens; sp, stratum pyramidale; sl, stratum lucidum. Scale bars: 200 µm (A, D, G); 75 µm (inset panels).

Figure S9. Normal cerebellar anatomy in *AU040320* and double KOs. Developing mutant cerebella show normal foliation pattern at P2 (A-D) and P10 (E-H). Thickness of external granule layer (EGL), Purkinje cell (CB+, red) layer (PCL) and internal granule layer (predominantly NeuN+ cells) are similar across genotypes at P2 and P10. Note that at P10, some Purkinje cells appear misaligned in mutants but this is also observed in wild-types, likely due to section thickness. Scale bar: 1000 µm (H); 200 µm (D); 50 µm (D', H').

Figure S10. Behavioural tests in dKO mice. (A) Motor coordination and learning in accelerating rotarod test is unaffected in dKOs as shown by latency to fall (s) from rotating rod during the 5 minute period of acceleration of the apparatus for each of the three consecutive days. There were no differences in the first day of testing (F=2.05; p=0.16) or in their performance after learning (Day 3, F=1.1; p=0.30). (B) Amplitude of startle reflex (measured in V) in response to noises of different sound intensity levels (dB SPL) for double KOs. Repeated-measures ANOVA indicated there was no effect of genotype on these responses, both across stimuli (stimulus as a within-subject factor; F=0.13; p=0.71), or with genotype x stimulus as factors (F=0.22; p=0.95). (C, D) Normal prepulse inhibition in *Kiaa0319* and *AU040320* single KOs. Data shows percent inhibition of startle amplitude when the startle-eliciting stimulus is preceded by prepulse cues of different sound intensity levels (dB SPL) above background noise (65 dB SPL; see protocol diagram in G), for *Kiaa0319* (C; n=9 per genotype; F=0.25; p=0.88) and *AU040320* KOs (D; n=10 per genotype; F=1.8; p=0.18). (E, F) Gap detection in *Kiaa0319* and *AU040320* single KO mice, quantified as percent inhibition of startle amplitude when the startle-eliciting stimulus is preceded by silent gaps in the background noise, for different gap durations (see protocol diagram in H). In both cohorts, % inhibition of acoustic startle increases with gap duration as expected, but no significant differences were observed between control and mutant groups (E, *Kiaa0319*, n=9, F=1.99, p=0.16; F, *AU040320*, n=10, F=1.2, p=0.28). All data shown as means ±SEM. (G) Schematic representation of prepulse inhibition protocol (see Supplementary Materials and Methods for details): top, description of protocol phases with components included in each of them; middle, diagram of protocol; bottom, detailed representation of the ten different stimuli included in each of the six blocks of the test sessions, presented in a randomised order; bottom lines indicate the recording

sampling windows. A simplified representation of the stimuli is shown in Fig. 4I. (H) Schematic representation of silent gap detection protocol (see Supplementary Materials and Methods for details): top, description of protocol phases with components included in each of them; bottom, detailed representation of the two types of trials (ten different stimuli in total) included in each of the six blocks of the test sessions, presented in a randomised order. A simplified representation of the stimuli is shown in Fig. 4J.

Figure S11. Click-evoked ABR wave latencies. (A, B) Click-evoked (10 clicks/sec) ABR wave latencies for waves I-IV in dKO and dWT mice (A), and in *Kiaa0319* KO, *AU040320* KO and age-matched sWT mice (B). Significant difference between *AU040320* KO and sWT mice for wave II latencies using RM-ANOVA test (p=0.027) but this was not confirmed by *post-hoc* Tukey tests for any suprathreshold sound level (all p>0.1). No other significant differences in wave latency between mutant and sWT mice for any of the waves (RM-ANOVA tests on data from 50-80 dB SPL clicks, all p>0.05; see Table S6). Data shown as mean ±SEM.

Figure S12. Click-evoked ABR wave amplitudes and latencies for slower click rate. Amplitudes (A) and latencies (B) of click-evoked (2 clicks/sec) ABR waves for *Kiaa0319* KO, *AU040320* KO, and sWT mice. *AU040320* KO but not *Kiaa0319* KO mice wave III amplitudes were significantly reduced compared to sWT (RM-ANOVA (group) p=0.005). Significant difference between *AU040320* KO and sWT mice for wave II and III latencies (RM-ANOVA wave II p=0.048; wave III p=0.048) not confirmed by *post-hoc* Tukey tests at any suprathreshold sound level (all p>0.2). Significant difference between *Kiaa0319* KO and sWT mice for wave II amplitude (RM-ANOVA p=0.009) not confirmed by *post-hoc* Tukey tests (all p>0.2). There were no other significant differences between single KOs and sWT (RM-ANOVA all p>0.05) (see Table S7). Data shown as mean ±SEM.

Figure S13. Click-evoked ABR wave amplitudes and latencies by gender. ABR wave amplitudes and latencies for an 80 dB SPL click (10 clicks/sec), for males and females of each group. (A, B) Percentage of males and females in each group. (C, D) Click-evoked ABR wave

amplitudes and latencies for double KO and dWT mice (C) and for single KO and sWT mice (D) for waves I-IV. For display purposes only 80dB SPL condition is shown; however, analysis was performed across sound levels (RM-ANOVA). Significant difference between dKO and dWT for wave III latency (RM-ANOVA (group x sound level x gender) p=0.0001; F=7.971). There were no other significant differences in wave amplitude or latency between mutants and wild-types due to gender for any wave (RM-ANOVA all p>0.05). Data shown as mean ±SEM.

Figure S14. Click-evoked ABR wave amplitudes and latencies by age. Scatter plots for ABR wave amplitudes and latencies evoked by an 80 dB SPL click (10 clicks/sec) as a function of age for the two cohorts. Lines show linear regression and data points indicate individual mice. (A, B) Click ABR amplitudes (A) and latencies (B) by age for ABR waves for dKO and dWT mice. (C, D) Click ABR amplitudes (C) and latencies (D) by age for ABR waves I-IV for single KO and sWT mice. Significant correlation was only found between age and wave I amplitude for *AU040320* KO mice (C, p=0.004).

Figure S15. ABR wave amplitudes and latencies to click following noise. ABR wave amplitudes and latencies for a probe click 20 ms after the end of a 200 ms noise versus a reference click 500 ms after the end of the noise for dKO (A, B) and single KO cohorts (C, D). For dKO cohort, click amplitudes (A) and latencies (B), there was a significant difference between dKO and dWT mice for wave II and III latencies with no outliers removed (binomial test, both p=0.003). Significant difference between dKO and dWT mice for wave II and IV latencies with one dWT outlier removed (binomial test, wave II p=0.022, wave IV p=0.003). An alternative analysis of probe click measures normalised by reference click measures for each animal revealed significant difference between dKO and dWT mice for wave III latency (Wilcoxon rank-sum test, p=0.018). For single KO cohorts, there was a significant difference for *AU040320* KO wave II amplitude (C) compared to sWT (binomial test, p=0.006). No other significant differences between groups were detected. Alternative analysis of probe click measures normalised by reference click measures for each animal confirmed significant difference between *AU040320* KO and sWT mice for wave II amplitude (data not shown; Wilcoxon rank-sum test, p=0.029).

Supplementary Tables

Table S1. Primers used in this work

				Position in *AU040320* alleles [b]				
Primer [a]				Wild type	*tm1a*	*tm1b*	*tm1c*	*tm1d*
**Name**	**Sequence**	**Reverse complementary to**	**Location**	***wt***	***KO1***	***del***	***Flox***	***Null***
mKLi2F2	CCACAGGTTAGTGACCATCT		intron 2	30318	30318	30318	30318	30318
mKLi2F7	TGGCAGACTGGCTTCCATAC		intron 2	32149	32149	32149	32149	32149
mKLi2F11	AGTTCACCGTTGCATGTCTC		intron 2	33888	33888	33888	33888	33888
SA-F1	AAGGCGCATAACGATACCAC		5'FRT(1)		34335	34335	34335	34335
IRES-F2	CGAAGCCGCTTGGAATAAGG		IRES		35548	35548		
lacZ-F3	AATCGCCTTGCAGCACATCC		lacZ		36160	36160		
lacZ-F5	GTTCAGATGTGCGGCGAGTT		lacZ		36817	36817		
lacZ-F8	AATCAGGCCACGGCGCTAAT		lacZ		37469	37469		
lacZ-F1	TGGCGAGCGATACACCGC		lacZ		38702	38702		
lacZ-F2	GCCCGTCAGTATCGGCGG		lacZ		39086	39086		
Neo-F1	CGCCACCATGATTGAACAAG		Neo		40044			
PNF	ATCCGGGGGTACCGCGTCGAG		5'FRT(2)		41283			
R2R	TCTATAGTCGCAGTAGGCGG		3'loxP(2)		41437		34533	
mKL3F1	TCTGCCTGCCACGCTCTATG		target, exon 3	34983	41984		35080	
mKL3F2	AGTCACCACCAGAGCTTACT		target, exon 3	35238	42239		35335	
mKLi3F4	GCAGCAGCTCTGGCATCGAA		intron 3	36207	43125	39994	36221	35001
mKLi3F11	TGCTGGCCTGCATCTTCTTC		intron 3	38837	45755	42624	38851	37631
mKLi2R11	GCAAGCAGGTGACAGATACT	AGTATCTGTCACCTGCTTGC	intron 2	34265	34265	34265	34265	34265
LAR3	CACAACGGGTTCTTCTGTTAGTCC	GGACTAACAGAAGAACCCGTTGTG	En-2 intron		34538	34538		
IRES-R3	CTGCTTCCTTCACGACATTC	GAATGTCGTGAAGGAAGCAG	IRES		35728	35728		
lacZ-R3	GTTCAACCACCGCACGATAG	CTATCGTGCGGTGGTTGAAC	lacZ		37030	37030		
lacZ-R5	GACATCCAGAGGCACTTCAC	GTGAAGTGCCTCTGGATGTC	lacZ		38103	38103		
lacZ-R7	AGGAGTCGTCGCCACCAATC	GATTGGTGGCGACGACTCCT	lacZ		39082	39082		
Neo-R2	GCAGGAGCAAGGTGAGATGA	TCATCTCACCTTGCTCCTGC	Neo		40379			
mKLi2R13	CCTCCAGAGTGCTGTGATTA	TAATCACAGCACTCTGGAGG	target, intron 2	34673	41674		34770	
mKL3R3	AGCCTAGCCTCAGAAGATGT	ACATCTTCTGAGGCTAGGCT	target, exon 3	35172	42173		35269	
mKLi3R2	AACTTGACGGCAGCTACACC	GGTGTAGCTGCCGTCAAGTT	intron 3 (wt)	35694				
loxP3R	GATGGCGAGCTCAGACCATA	TATGGTCTGAGCTCGCCATC	3'loxP(3)		42627	39496	35723	34503
mKLi3R4	AGTGCCTGGCAAGTAGTAAC	GTTACTACTTGCCAGGCACT	intron 3	36061	42979	39848	36075	34855
mKLi3R6	TCGACCTGAGTGAGAATGTG	CACATTCTCACTCAGGTCGA	intron 3	36790	43708	40577	36804	35584
mKLi3R11	CCAGGTAGTGGTAGTGCATA	TATGCACTACCACTACCTGG	intron 3	39171	46089	42958	39185	37965
mKLi3R14	CCAGCAGTGGAGGAGTGTTC	GAACACTCCTCCACTGCTGG	intron 3	40064	46982	43851	40078	38858

a: All primers used in sequencing and genotyping PCRs are described; for "reverse" primers, the reverse complementary sequence is also provided. Other primers required for some genotyping PCRs have been described before (Martinez-Garay et al. 2016)

b: Position of first nt of primer, taking A in ATG codon as position +1, in the different *AU040320* alleles (the two notations used in the text are shown)

Table S2. Genotyping and sequencing PCRs for *AU040320* -targeted mice

Genotyping / sequencing PCRs [a]			Expected band sizes (bp) [b]					Expected PCR Result (top) / mice used (bottom) [c]								
**PCR**	**Primers**		***wt***	***tm1 a***	***tm1 b***	***tm1 c***	***tm1 d***	**w/w**	**a/w**	**a/a**	**b/w**	**b/b**	**c/w**	**c/c**	**d/w**	**d/d**
**name**	**Forward**	**Reverse**	***wt***	***KO1***	***del***	***Flx***	***Null***	**w/w**	**K/w**	**K/K**	**del/w**	**del/del**	**F/w**	**F/F**	**N/w**	**N/N**
**KLW**	mKL3F1	mKLi3R2	**712**					+	+	–	+	–	+	–	+	–
**KLK**	PNF	mKL3R3		**891**				–	+	+	–	–	–	–	–	–
**KLKFa**	R2R	mKL3R3		**737**		**737**		–	+	+	–	–	+	+	–	–
**KLKFb**	mKL3F1	loxP3R		**644**		**644**		–	+	+	–	–	+	+	–	–
**KLKZ**	mKLi2F11	LAR3		**651**	**651**			–	+	+	+	+	–	–	–	–
**KLZ**	lacZ-F2	mKLi3R4		3894	**763**			–	–	–	+	+	–	–	–	–
**KLWF**	mKLi2F11	mKLi2R13	**786**	7787		**[883]**		+	+	–	+	–	[+]+	[+]	+	–
**KLNF**	SA-F1	mKLi3R4		8645	5514	**[1741]**	**521**	–	–	–	–	–	([+])	([+])	+	+
**4**	lacZ-F8	lacZ-R5		**635**	**635**			–	+	+	+	+	–	–	–	–
**4a**	lacZ-F3	lacZ-R3		**871**	**871**			–	+	+	+	+	–	–	–	–
**KO1-5'**	mKLi2F2	LAR3		**4221**	4221					X						
**5'a-4**	mKLi2F2	mKLi2R11	3948	**3948**	3948	3948	3948			X						
**5'a-7**	mKLi2F7	LAR3		**2390**	2390				X							
**C-1**	mKLi2F11	IRES-R3		**1841**	1841				X							
**C-2**	IRES-F2	lacZ-R3		**1483**	1483				X							
**C-3**	lacZ-F5	lacZ-R5		**1287**	1287				X							
**C-4**	lacZ-F8	lacZ-R7		**1614**	1614				X							
**C-5**	lacZ-F1	Neo-R2		**1678**					X							
**TE-1**	Neo-F1	loxP3R		**2584**					X							
**3'a-14**	mKL3F2	mKLi3R6	1553	**1470**		1470				X						
**3'a-4**	mKLi3F4	mKLi3R11	2965	**2965**	2965	2965	2965			X						
**3'a-19**	mKLi3F11	mKLi3R14	1228	**1228**	1228	1228	1228			X						
**KLZ**	lacZ-F2	mKLi3R4		3894	**763**						X					
**Flx-check**	mKLi2F11	loxP3R		8740	5609	**1836**	616						X			

a: Genotyping and KO1-5' PCRs are depicted in Fig. 1; primer sequence and location details are shown in Fig. S2 and Table S1

b: Size of bands relevant for each PCR are shown in bold and bigger font. Alleles on top of the table are shown using the two notations mentioned in the text

c: Results on top of the table are shown as presence (+) or absence (–) of genotyping PCR band; [+] indicates the fragment in square brackets on the left. Genotypes on top of the table are shown using an abbreviated notation of alleles

Table S3. Primary antibodies used in Immunostaining experiments.

Antibody (anti-)	Species	Dilution	Company	Cat.#
Actin [C4]	mouse monoclonal	1:1000	BD Transduction Laboratories	612656
Calbindin D-28k	rabbit polyclonal	1:2000	Swant	CB38
Calretinin	rabbit polyclonal	1:2500	Swant	7697
Cre [7-23]	mouse monoclonal	1:200	Sigma-Aldrich	C7988
Ctgf	goat polyclonal	1:2000	Santa Cruz Biotechnology	sc-14939
Ctip2 [25B6]	rat monoclonal	1:500	Abcam	ab18465
Cux1 [CDP (M-222)]	rabbit polyclonal	1:200	Santa Cruz Biotechnology	sc-13024
Ki67	rabbit polyclonal	1:1000	Abcam	ab15580
NeuN [A60]	mouse monoclonal	1:100	Chemicon	MAB377
NF-H (Neurofilament H) [SMI32]	mouse monoclonal	1:2500	Covance	SMI-32P
Pax6	rabbit polyclonal	1:200	Covance	PRB-278P
Parvalbumin	rabbit polyclonal	1:5000	Swant	PV28
pH3 (Histone H3 (phospho S10))	mouse monoclonal	1:2000	Abcam	ab14955
Reelin [G10]	mouse monoclonal	1:500	Chemicon	MAB5364
Somatostatin [SOM-018]	mouse monoclonal	1:250	GeneTex	GTX71935
TBR2	rabbit polyclonal	1:500	Abcam	ab23345

Table S4. Body weight (g) of *AU040320* and double KO mutants.

	Genotype [a]		Age		
**Group**	***Kiaa***	***AU***	**P2**	**P10**	**Adult (P60)**
Wild-type	**+/+**	**+/+**	1.84 (±0.10); n= 8	5.41 (±0.35); n=10	20.77 (±0.81); n=18
AU het.	**+/+**	**+/–**	1.76 (±0.08); n=10	5.18 (±0.32); n=14	-
AU KO	**+/+**	**–/–**	1.61 (±0.05); n=11	5.58 (±0.22); n= 8	-
Double KO	**–/–**	**–/–**	-	-	22.04 (±0.93); n=18

a: +, wild-type allele; -, *del* (*tm1b*) allele. *Kiaa*, *Kiaa0319*; *AU*, *AU040320*

Table S5. Results of RM-ANOVA tests for click (10 clicks/sec) ABR amplitudes in dKO versus dWT mice, and *AU040320*-KO or *Kiaa0319*-KO vs sWT.

**Amplitudes, 10 clicks/sec**	RM-ANOVA (sound level x group)	RM-ANOVA (group)	*Post-hoc* Tukey test (where applicable) [a]			
			50dB SPL	60dB SPL	70dB SPL	80dB SPL
**dKO vs dWT**						
Wave I	**p=0.002; F=5.536**	p=0.079; F=3.401	0.132	0.251	0.208	**0.016**
Wave II	**p=0.044; F=2.855**	p=0.336; F=0.968	0.632	0.530	0.394	0.140
Wave III	p=0.317; F=1.200	**p=0.014; F=7.132**	**0.010**	**0.009**	**0.019**	**0.036**
Wave IV	p=0.258; F=1.375	p=0.053; F=4.190	0.562	0.210	0.055	0.101
***AU040320*-KO vs sWT**						
Wave I	p=0.209; F=1.550	p=0.896; F=0.018	0.149	0.794	0.745	0.408
Wave II	p=0.061; F=2.563	p=0.679; F=0.176	0.114	0.334	0.771	0.343
Wave III	p=0.339; F=1.141	**p=0.029; F=5.405**	**0.036**	**0.024**	0.109	**0.031**
Wave IV	p=0.426; F=0.940	p=0.240; F=1.451	0.481	0.261	0.895	0.020
***Kiaa0319*-KO vs sWT**						
Wave I	p=0.853; F=0.261	p=0.695; F=0.158	0.244	0.657	0.742	0.984
Wave II	p=0.366; F=1.075	p=0.923; F=0.010	0.522	0.742	0.839	0.448
Wave III	p=0.517; F=0.766	p=0.651; F=0.210	0.534	0.347	0.927	0.938
Wave IV	p=0.633; F=0.576	p=0.717; F=0.135	0.574	0.423	0.985	0.474

a**:** *post-hoc* Tukey test results shown where RM-ANOVA result was significant. RM-ANOVA tests were conducted only on suprathreshold data (50-80 dB SPL clicks). P-values deemed significant (using an alpha value of 0.05) are in bold.

Table S6. Results of RM-ANOVA tests for click (10 clicks/sec) ABR latencies in dKO versus dWT mice, and *AU040320*-KO or *Kiaa0319*-KO vs sWT.

**Latencies, 10 clicks/sec**	RM-ANOVA (sound level x group)	RM-ANOVA (group)	*Post-hoc* Tukey test (where applicable)			
			50dB SPL	60dB SPL	70dB SPL	80dB SPL
**dKO vs dWT**						
Wave I	p=0.594<br>F=0.637	p=0227<br>F=1.546	0.481	0.134	0.218	0.213
Wave II	p=0.229<br>F=1.477	p=0.318<br>F=1.046	0.589	0.259	0.204	0.287
Wave III	p=0.211<br>F=1.546	p=0.447<br>F=0.599	0.630	0.467	0.322	0.398
Wave IV	p=0.213<br>F=1.539	p=0.483<br>F=0.509	0.700	0.531	0.408	0.341
***AU040320*-KO vs sWT**						
Wave I	p=0.537<br>F=0.732	p=0.288<br>F=1.183	0.458	0.262	0.116	0.979
Wave II	**p=0.027<br>F=3.241**	p=0.804<br>F=0.063	0.143	0.702	0.926	0.622
Wave III	p=0.239<br>F=1.438	p=0.216<br>F=1.613	0.159	0.159	0.300	0.350
Wave IV	p=0.251<br>F=1.398	p=0.681<br>F=0.173	0.434	0.766	0.636	0.949
***Kiaa0319*-KO vs sWT**						
Wave I	p=0.804<br>F=0.330	p=0.264<br>F=1.309	0.625	0.154	0.163	0.715
Wave II	p=0.410<br>F=0.975	p=0.400<br>F=0.736	0.689	0.642	0.397	0.218
Wave III	p=0.552<br>F=0.706	p=0.241<br>F=1.446	0.202	0.263	0.287	0.360
Wave IV	p=0.936<br>F=0.140	p=0.589<br>F=0.300	0.729	0.526	0.656	0.496

Conventions as in Table S5.

Table S7. Results of RM-ANOVA tests for slow-click rate (2 clicks/sec) ABR amplitudes and latencies in *AU040320*-KO or *Kiaa0319*-KO vs sWT.

**2 clicks/sec**	RM-ANOVA (sound level x group)	RM-ANOVA (group)	*Post-hoc* Tukey test (where applicable)			
			50dB SPL	60dB SPL	70dB SPL	80dB SPL
***AU040320*-KO vs sWT; Amplitudes**						
Wave I	p=0.704; F=0.470	p=0.849; F=0.037	0.746	0.884	0.770	0.696
Wave II	p=0.301; F=1.242	p=0.297; F=1.137	0.051	0.335	0.175	0.956
Wave III	p=0.056; F=2.640	**p=0.005; F=9.347**	**0.024**	**0.002**	**0.010**	**0.012**
Wave IV	p=0.204; F=1.569	p=0.233; F=1.496	0.234	0.165	0.200	0.932
***AU040320*-KO vs sWT; Latencies**						
Wave I	p=0.335 F=1.149	p=0.578 F=0.317	0.708	0.217	0.636	0.815
Wave II	**p=0.048 F=2.759**	p=0.951 F=0.004	0.403	0.921	0.955	0.479
Wave III	**p=0.048 F=2.759**	p=0.336 F=0.965	0.250	0.220	0.439	0.600
Wave IV	p=0.818 F=0.310	p=0.898 F=0.017	0.861	0.980	0.987	0.720
***Kiaa0319*-KO vs sWT; Amplitudes**						
Wave I	p=0.677 F=0.510	p=0.879 F=0.024	0.936	0.892	0.784	0.718
Wave II	**p=0.009 F=4.186**	p=0.918 F=0.011	0.401	0.303	0.754	0.247
Wave III	p=0.394 F=1.009	p=0.692 F=0.161	0.752	0.558	0.316	0.668
Wave IV	p=0.127 F=1.968	p=0.824 F=0.051	0.927	0.487	0.768	0.031
***Kiaa0319*-KO vs sWT; Latencies**						
Wave I	p=0.767 F=0.381	p=0.101 F=2.920	0.089	0.130	0.175	0.220
Wave II	p=0.877 F=0.228	p=0.382 F=7.94	0.429	0.464	0.511	0.284
Wave III	p=0.913 F=0.176	p=0.262 F=1.322	0.329	0.243	0.222	0.349
Wave IV	p=0.428 F=0.936	p=0.519 F=0.428	0.888	0.611	0.313	0.384

Conventions as in Table S5.

Genome assembly: **GRCm38.p5**
MGI transcript name: **AU040320-003**
Gene **AU040320**
Ensembl Gene ENSMUSG00000028830
Location Chromosome 4: 126,753,544-126,870,070
Ensembl Transcript ENSMUST00000047431
Ensembl Protein ENSMUSP00000037802
Strand Forward
Base pairs 4,384 126,756,927 ATG codon start position (exon 2)
Amino acids 1,048

				Phase						
**No.**	**Exon / Intron**	**Start**	**End**	**Start**	**End**	**Length**				
	5' upstream sequence									
1	ENSMUSE00000446321	126,753,770	126,753,995	-	-	226				
	Intron 1-2	126,753,996	126,756,898			2,903				
2	ENSMUSE00001229254	126,756,899	126,757,068	-	1	170	**Length**	**region**	**Size in allele**	
	Intron 2-3	126,757,069	126,787,548			34,706	30,480		WT	KO1
		126,787,549	126,791,260				3,712	5'arm	3,712	3,712
		126,791,261	126,791,381				121		121	7,122
		126,791,382	126,791,774				393	target	1,097	1,097
3	ENSMUSE00000331250	126,791,775	126,792,295	1	0	521	521			
	Intron 3-4	126,792,296	126,792,478			21,960	183			
		126,792,479	126,792,641				163		163	80
		126,792,642	126,796,726				4,085	3'arm	4,085	4,085
		126,796,727	126,814,255				17,529			
4	ENSMUSE00000233523	126,814,256	126,814,502	0	1	247				
	Intron 4-5	126,814,503	126,816,372			1,870				
5	ENSMUSE00000233515	126,816,373	126,816,474	1	1	102				
	Intron 5-6	126,816,475	126,823,643			7,169				
6	ENSMUSE00001301214	126,823,644	126,823,741	1	0	98				
	Intron 6-7	126,823,742	126,828,800			5,059				
7	ENSMUSE00001306363	126,828,801	126,828,888	0	1	88				
	Intron 7-8	126,828,889	126,830,329			1,441				
8	ENSMUSE00001260521	126,830,330	126,830,422	1	1	93				
	Intron 8-9	126,830,423	126,832,089			1,667				
9	ENSMUSE00000233484	126,832,090	126,832,222	1	2	133				
	Intron 9-10	126,832,223	126,835,470			3,248				
10	ENSMUSE00000382161	126,835,471	126,835,699	2	0	229				
	Intron 10-11	126,835,700	126,836,634			935				
11	ENSMUSE00001233590	126,836,635	126,836,758	0	1	124				
	Intron 11-12	126,836,759	126,837,286			528				
12	ENSMUSE00001244605	126,837,287	126,837,419	1	2	133				
	Intron 12-13	126,837,420	126,839,598			2,179				
13	ENSMUSE00001224440	126,839,599	126,839,747	2	1	149				
	Intron 13-14	126,839,748	126,840,645			898				
14	ENSMUSE00000233342	126,840,646	126,840,797	1	0	152				
	Intron 14-15	126,840,798	126,841,105			308				
15	ENSMUSE00000182520	126,841,106	126,841,244	0	1	139				
	Intron 15-16	126,841,245	126,842,475			1,231				
16	ENSMUSE00000182524	126,842,476	126,842,635	1	2	160				
	Intron 16-17	126,842,636	126,846,357			3,722				
17	ENSMUSE00000182522	126,846,358	126,846,500	2	1	143				
	Intron 17-18	126,846,501	126,847,140			640				
18	ENSMUSE00000182525	126,847,141	126,847,263	1	1	123				
	Intron 18-19	126,847,264	126,847,776			513				
19	ENSMUSE00000182523	126,847,777	126,847,867	1	2	91				
	Intron 19-20	126,847,868	126,848,810			943				
20	ENSMUSE00000524916	126,848,811	126,848,902	2	1	92				
	Intron 20-21	126,848,903	126,853,165			4,263				
21	ENSMUSE00000233304	126,853,166	126,854,336	1	-	1,171				
	3' downstream sequence									

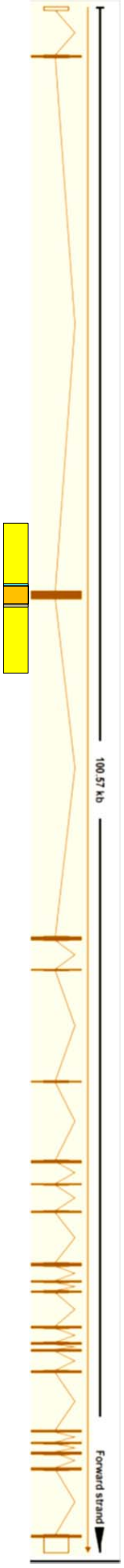

126,853,353 Stop codon end position (exon 21)

Figure S1

Figure S2. *AU040320* knockout-first allele

TAAAAAGGACTCCCTCTCAGACTGGCAAGATGGTTCAGTATGTAAAGCTGCTTGCTGCCAATCCTGAAAACTTGAGTTCC
ATTCCTATGAACCATATAATGGAAGAAGAGAACCGACTTCAGCAAGTTGTCTTCTGATCTCCTCTGTGCACACACAAATC
AAAAACACACTGGCCACGCCCCAGGGATTATTTTTTTAATTTATTTTTAATGTGTGTCTGTCTGTCTGTCTGTCTATCT
GTCTGTCTGTCTGTCTGAGTATGGGTTGGTTCACTTGAGTGCAGTCCTGAGGGGGTCAGAAAGGGGCATTTGGTCTCATG
GAACTGGAGTTATAAGCAGTTGTCAACTGCCTTGTGGGTACTGGGCATTGAACTCTCATCCTTTGCAAGAGCAGCACATG
ATCATAATCGCTGAGCCATCTCACCAGCCCCAGAGACTTCTTTACATTGGTGAATCACTGGGGTAGTGGCCACAGGTTAG
mKLi2F2 TGACCATCTGTCATGCACACAAGAAGAAAGGCCCTCTCAGATTCAAGCAGTACAGTAGTATTTCTTATCCAAGAATCCTC
ACAGACCCCTAGATTCTAGTCCCTACACCTGCCAACAGCCTGAGTTTGTGATTGGTCACTTCATAATTGAAGGAACTTTC
TTCTTGGTAATGGGTGTACTCTGTCTGGCAGGCCAGTGATGGGTCTCACGGAAAACTGTCTTTATAGGTTTCAGCTTGTG
CTTTTGGAAGGACCAGTTCTACGTCAACAGTCTTCCTACGATTCTCTGAGCTCCTCATAGATCTCCTTTAGCTCTTTTGT
ATTTTGAACCATTAAACTTCAGTTCTCTTGAGTGGCTGTGTGTGCTAGATTTTCTCTCATGGTCCCTCCAAATGTTGGTG
TTTTCTCTTTTGAAAGCTGGATGCAGCTACTCTACTTAATCCTAACCATCTTTTTCTGTTGACATAAGTACCATGTCGGT
ACAATGGTTACTCTGATCACCTCATTCAGATTCCTCTTGTTGTCTTAACTCTTTTCCTATACAGACGTTACCCATGGTAG
CTTCTCCCTCTGTGCCTCAGTAGCTCCTTCACAGGACACTGTCTGTCAGAGCCTCGTGTTTCTAAGAGTTGGGCAGGTAG
ACAGAGAATGTACTCATCATTTTCACAGAAGACTCTTGCTTTCCTCATGTGTCACTCATACATATTTTTCTGAGCTTAAC
AGTTGCCTGAGGCTGATTTGTCTGGCAGTGGATACCGGATTCTTTACATGAATGCCAGTTATATATCATATATATCTGTG
CTGGGATGATTCCCTGGACTGAGGGTAACAGACAGGACTTGAACCTGATCCCTGAGATGATTGCTGTCAAAGTGATACCC
AATGACTTTAGGAATCAGAACGTCACAGTGCAGAACTGGTGACTTCAAAGTCTCCAAGGCCAGGTGAGTTTAGAATTCAT
ACCTCTGTTGTGGAGATATGCTAAAGGCTTTATTACATATATGTCCATTGCCTCTCATCTCTACAGCTGCTCGGCTGTGA
ATCCTAGAGAGTGATTTAGAGATGACAGTGGGATGGCTTGTTCATGGAAATCACGACACGGTCATTACCTTTCATGAGTA
TTGTCGTGATGATGTCTCTGGTGAGTAATAAAGGAGATCTGAAGACTTGCTACAAGGTTGCTGTCTTGGAATATTTCGGA
GTTGTTTTGATGATACAAAGGTAGCTAGCAGTATTTACTGACTTATTTACTTATTAATACAGCACCTTACTGTGTAGCTC
TGGATATGTGTGTGTGTGTGTGTGTGTGTGTGTGTGTGTGTGTGTGTGTGTGTGCACGCACACATGCAGCACTCACTTTTAA
TCCCAGCACTTGGGAGGCAGAGGCAGGAAGATCTCTGATTTCGAGGCCAGCCTGGTCTATAACATGAGTTCCAGACAGCC
AGGGCTACACAGAGAAGACCTGTCTCAAAAACAATCAAACAAACAAAACCAACAAAAAATAAATAAATAAATAAATAAAA
TAAAAAATAGAGGTGTGTGCCACCATGCCTGGCTTTTACCATTGTTTATAAATCACCAAGCCACAGTTTATTGCTCATAG
CAATGACTGTAGACACAGCTCTTACCAAGCCTCATTCATGCTGTGAATGGCTGTGGAGAGGTAGATAAAGACACAGAGGG
TATTTCCATGGCTTCTTTTTTATTTTGGCTTTGTTATTTCCTTGTCTTTTCTTAGGGAAGTTCTGTTTACTCTGATTATC
mKLi2F7 TAAATTGCCTTGTCTGGTGTTAGGTTGGTGCCAATGTAGCTCTCATTTTTCAGAGAATAATGGCAGACTGGCTTCCATAC
ATAGAACTTCTGACTCAGTAGTCTAGAAGTCTGCCTTTGTCTTCTTTTTATTTCCCAGTTGATTAGTGTCCACATTTAGT
CTGTTGGATATTTAGTGAATATTTCGTGGGAGTCAGATTGGTTCTGGCCAATTAACAGGTTGTTGCTTTAGTTGGAGTGT
GTTTTCAGTTATGTGTATATGCAAATGTACTCATTCATTTCCTCAATGCTCTGATTCTATTGCATGTTTTCTATCTGTAA
TTTAAATCTACACACATGCATGTGCATGCACACATCTACATTTTCTTTTTCCTGTTGTCATATGTGTGTGTGCAGCATGC
ATGTATGTAGTATGCTTGCATGTGTTTGAGCAGAAGTATATGTGGAGGCCTGAAGTTGATATCAGGAATCGCCCTCCATC
ACTCTTCCATGCACTCAGTGTGAGGAGGCAGGTCTCCGTCAGCCTGAGAGAGAGCTCATAGATATGGCTTGCTGGCTTGT
TCTGGGCATCTCTTGTCTCTGCCTTCTGAGGCAGATCTATCTTCCTGGCATTTACTTGAGTTCTTAAGATCCAAAATCCG
GTCCTCCCTTTGTGTGGCCCATGCTAACTACCTAGCTATCTAATATACCAGCTCTCAAATATATTTCCTGTGTTATAATT
TCAGGGACAGGACTAAGCCTATAAAAGAGAGTGCTCTTTTGTGAGTCAATAGAAGGTTTAAAGAGGTGTTCTCAGTTGGG
TATTGTAGTGCAACCCTGGCACTTCAGAGGTAGGGACAGGAGGATCAGGAGCTCAAGGCTATCCTTGGCTACATTGTAAA
TTTGAGGAAAAACAGCAAGGTCTGTAATCCAGCTGTTAGGAGAGACTGAGAGTAGGATTCAAGTTCAGTGTCTGCCTGGT
GCACAGTGAGTTCAGAGCCACCCTGAAACCCTTAGTAAAGATTAAAAGTGGATGAAGAGATAGCCAGTGGTAGAGTACTT
ACACACATGCCTGATGCCCTGTTTACTCTCCAGTACTGTGGGGTGGGTTGGAGGGAGGACCTAGCTAAGTTTTATATCAG 5’ arm
GCTAGGATCTGGTGGAGGGTGCTGACTAGTTTTATGTCAACTTGACACATTCTAGAGGAGGGAAGCCTAATTGAGAAAAT
TCATTTTGAGATTGGGCTAGGACAGGCTGGAGAGATGGCTCAGAGGTTAGGAGCACTGACTTCTCTTCCGAAGGTTCTGG
GTTCAATTCCCAGCAACCACATGGTGGCTCACAGCCATCTGTAATGGGATCTGGTGCCCTCTTCTGGTACTCATATACAT
AAAATAAATAATATTTTTTTAAAAATGGGTTGGGGCAGGGAAGCCTATAGGACATTTTCTTGTTGAATAATTCATGAGGG
AGGGCTTGGGCCATTATGGATGGAGATACTCCTGGGCTGGTGGTCCTGGGCTCTATAAGAAAGCAGTCTGAGGAAACCAG
CTGCATCAGTTCCTGCCTCCAGGTCCCTGTGTGCTTGAGCTCCTGCCCTCCCTGCTTTGGTAATGGACTGTCACATGGGA
CTGTGAGTGAAATAAACCCTTCCCTGCCCAAGCTGCTTTTTGTCAGTGTTCCATCCCAATGGAAACCCTAAGACGGACTC
AAAACAACCTCCATTTACAGCTGGTTTTGTCAGAGGTAATGATGAAGCGCATCAATCCCATGCCTGACATAGTTCAACTG
mKLi2F11 AGAATCTGGGATCATGGTATTATCCAGATTACCTATAGTAGTTCACCGTTGCATGTCTCCTTGTATTTGTGTGGTTTGGT
ATGGTTGTCATTCAGAAAATCCAGTTAGGATGATTGCTCTCATTATTACAATTTTTGGTAATTTTCACTTGTACTTTGGC
TAATGACACAATATGCAAAATCATGTGACTGGCAAACAACTCATGAGAAAATACAATATCGCCGGGCGTGGTGGCGCACA
CCTTTAATCCCAGCACTCGGGAGGCAGAGGCAGGCGGATTTCTGAGTTCGAGGCCAGCCTGGTCTACAAAGTGAGTTCCA
GGACAACCAGGGCTATACAGAGAAACCCTGTCTTGAAAAACAAAAACAAAAAAAACAAAAAACAAAAAAGAAAATAC**AGT**
ATCTGTCACCTGCTTGCGGTCAGGAGAGCTCAGCCTGTGTTTCTGTTAGTTCAGTGGGAAAATGTTGCAGCTTGTTCATC **mKLi2R11**
TTCCTA

CACTTCTGGTCACTTTGTCCACTCCCCCTCATGTACACCTTGGAAGCAAGTTAGAAGGTCA
AGTTTGGGGACTCTGCACTTCCGTCAGAAGAGTCCGTCTTAAATTTTATTTTATATTTTG

Segment of intron 2 replaced by trapping cassette in KO1 allele

Trapping cassette

SA-F1 AAGGCGCATAACGATACCACGATATCAACAAGTTTGTACAAAAAAGCAGGCTGGCGCCGGAACCGAAGTTCCTATTCCGA
AGTTCCTATTCTCTAGAAAGTATAGGAACTTCGAACCCTTTCCCACACCACCCTCCACACTTGCCCCAAACACTGCCAAC FRT
TATGTAGGAGGAAGGGGTTG**GGACTAACAGAAGAACCCGTTGTG**GGGAAGCTGTTGGGAGGGTCACTTTATGTTCTTGCC **LAR3**
CAAGGTCAGTTGGGTGGCCTGCTTCTGATGAGGTGGTCCCAAGGTCTGGGGTAGAAGGTGAGAGGGACAGGCCACCAAGG
TCAGCCCCCCCCCCCTATCCCATAGGAGCCAGGTCCCTCTCCTGGACAGGAAGACTGAAGGGGAGATGCCAGAGACTCAG
TGAAGCCTGGGGTACCCTATTGGAGTCCTTCAAGGAAACAAACTTGGCCTCACCAGGCCTCAGCCTTGGCTCCTCCTGGG
AACTCTACTGCCCTTGGGATCCCCTTGTAGTTGTGGGTTACATAGGAAGGGGGACGGGATTCCCCTTGACTGGCTAGCCT
ACTCTTTTCTTCAGTCTTCTCCATCTCCTCTCACCTGTCTCTCGACCCTTTCCCTAGGATAGACTTGGAAAAAGATAAGG En2
GGAGAAAACAAATGCAAACGAGGCCAGAAAGATTTTGGCTGGGCATTCCTTCCGCTAGCTTTTATTGGGATCCCCTAGTT ‘intron_1/
TGTGATAGGCCTTTTAGCTACATCTGCCAATCCATCTCATTTTCACACACACACACACCACTTTCCTTCTGGTCAGTGGG exon_2’
CACATGTCCAGCCTCAAGTTTATATCACCACCCCCAATGCCCAACACTTGTATGGCCTTGGGCGGGTCATCCCCCCCCCC
ACCCCCAGTATCTGCAACCTCAAGCTAGCTTGGGTGCGTTGGTTGTGGATAAGTAGCTAGACTCCAGCAACCAGTAACCT
CTGCCCTTTCTCCTCCATGACAACCAGGTCCCAGGTCCCGAAAACCAAAGAAGAAGAACCCTAACAAAGAGGACAAGCGG
CCTCGCACAGCCTTCACTGCTGAGCAGCTCCAGAGGCTCAAGGCTGAGTTTCAGACCAACAGGTACCTGACAGAGCAGCG
GCGCCAGAGTCTGGCACAGGAGCTCGGTACCCGGAAGATCTGGACTCTAGAGAATTCCGCCCCTCTCCCTCCCCCCCCCC
IRES-F2 TAACGTTACTGGCCGAAGCCGCTTGGAATAAGGCCGGTGTGCGTTTGTCTATATGTTATTTTCCACCATATTGCCGTCTT
TTGGCAATGTGAGGGCCCGGAAACCTGGCCCTGTCTTCTTGACGAGCATTCCTAGGGGTCTTTCCCCTCTCGCCAAAGGA
ATGCAAGGTCTGTT**GAATGTCGTGAAGGAAGCAG**TTCCTCTGGAAGCTTCTTGAAGACAAACAACGTCTGTAGCGACCCT **IRES-R3**
TTGCAGGCAGCGGAACCCCCCACCTGGCGACAGGTGCCTCTGCGGCCAAAAGCCACGTGTATAAGATACACCTGCAAAGG
CGGCACAACCCCAGTGCCACGTTGTGAGTTGGATAGTTGTGGAAAGAGTCAAATGGCTCTCCTCAAGCGTATTCAACAAG
GGGCTGAAGGATGCCCAGAAGGTACCCCATTGTATGGGATCTGATCTGGGGCCTCGGTGCACATGCTTTACATGTGTTTA IRES
GTCGAGGTTAAAAAAaCGTCTAGGCCCCCCGAACCACGGGGACGTGGTTTTCCTTTGAAAAACACGATGATAAGCTTGCC
ACAACCATGGAAGATCCCGTCGTTTTACAACGTCGTGACTGGGAAAACCCTGGCGTTACCCAACTTAATCGCCTTGCAGC
lacZ-F3 ACATCCCCCTTTCGCCAGCTGGCGTAATAGCGAAGAGGCCCGCACCGATCGCCCTTCCCAACAGTTGCGCAGCCTGAATG
GCGAATGGCGCTTTGCCTGGTTTCCGGCACCAGAAGCGGTGCCGGAAAGCTGGCTGGAGTGCGATCTTCCTGAGGCCGAT
ACTGTCGTCGTCCCCTCAAACTGGCAGATGCACGGTTACGATGCGCCCATCTACACCAACGTGACCTATCCCATTACGGT *lacZ*
CAATCCGCCGTTTGTTCCCACGGAGAATCCGACGGGTTGTTACTCGCTCACATTTAATGTTGATGAAAGCTGGCTACAGG
AAGGCCAGACGCGAATTATTTTTGATGGCGTTAACTCGGCGTTTCATCTGTGGTGCAACGGGCGCTGGGTCGGTTACGGC
CAGGACAGTCGTTTGCCGTCTGAATTTGACCTGAGCGCATTTTTACGCGCCGGAGAAAACCGCCTCGCGGTGATGGTGCT
GCGCTGGAGTGACGGCAGTTATCTGGAAGATCAGGATATGTGGCGGATGAGCGGCATTTTCCGTGACGTCTCGTTGCTGC
ATAAACCGACTACACAAATCAGCGATTTCCATGTTGCCACTCGCTTTAATGATGATTTCAGCCGCGCTGTACTGGAGGCT
lacZ-F5 GAAGTTCAGATGTGCGGCGAGTTGCGTGACTACCTACGGGTAACAGTTTCTTTATGGCAGGGTGAAACGCAGGTCGCCAG
CGGCACCGCGCCTTTCGGCGGTGAAATTATCGATGAGCGTGGTGGTTATGCCGATCGCGTCACACTACGTCTGAACGTCG
AAAACCCGAAACTGTGGAGCGCCGAAATCCCGAATCT**CTATCGTGCGGTGGTTGAAC**TGCACACCGCCGACGGCACGCTG **lacZ-R3**
ATTGAAGCAGAAGCCTGCGATGTCGGTTTCCGCGAGGTGCGGATTGAAAATGGTCTGCTGCTGCTGAACGGCAAGCCGTT
GCTGATTCGAGGCGTTAACCGTCACGAGCATCATCCTCTGCATGGTCAGGTCATGGATGAGCAGACGATGGTGCAGGATA
TCCTGCTGATGAAGCAGAACAACTTTAACGCCGTGCGCTGTTCGCATTATCCGAACCATCCGCTGTGGTACACGCTGTGC
GACCGCTACGGCCTGTATGTGGTGGATGAAGCCAATATTGAAACCCACGGCATGGTGCCAATGAATCGTCTGACCGATGA
TCCGCGCTGGCTACCGGCGATGAGCGAACGCGTAACGCGAATGGTGCAGCGCGATCGTAATCACCCGAGTGTGATCATCT
lacZ-F8 GGTCGCTGGGGAATGAATCAGGCCACGGCGCTAATCACGACGCGCTGTATCGCTGGATCAAATCTGTCGATCCTTCCCGC
CCGGTGCAGTATGAAGGCGGCGGAGCCGACACCACGGCCACCGATATTATTTGCCCGATGTACGCGCGCGTGGATGAAGA
CCAGCCCTTCCCGGCTGTGCCGAAATGGTCCATCAAAAAATGGCTTTCGCTACCTGGAGAGACGCGCCCGCTGATCCTTT
GCGAATACGCCCACGCGATGGGTAACAGTCTTGGCGGTTTCGCTAAATACTGGCAGGCGTTTCGTCAGTATCCCCGTTTA
CAGGGCGGCTTCGTCTGGGACTGGGTGGATCAGTCGCTGATTAAATATGATGAAAACGGCAACCCGTGGTCGGCTTACGG
CGGTGATTTTGGCGATACGCCGAACGATCGCCAGTTCTGTATGAACGGTCTGGTCTTTGCCGACCGCACGCCGCATCCAG
CGCTGACGGAAGCAAAACACCAGCAGCAGTTTTTCCAGTTCCGTTTATCCGGGCAAACCATCGAAGTGACCAGCGAATAC
CTGTTCCGTCATAGCGATAACGAGCTCCTGCACTGGATGGTGGCGCTGGATGGTAAGCCGCTGGCAAGCG**GTGAAGTGCC** **lacZ-R5**
TCTGGATGTCGCTCCACAAGGTAAACAGTTGATTGAACTGCCTGAACTACCGCAGCCGGAGAGCGCCGGGCAACTCTGGC
TCACAGTACGCGTAGTGCAACCGAACGCGACCGCATGGTCAGAAGCCGGGCACATCAGCGCCTGGCAGCAGTGGCGTCTG
GCGGAAAACCTCAGTGTGACGCTCCCCGCCGCGTCCCACGCCATCCCGCATCTGACCACCAGCGAAATGGATTTTTGCAT
CGAGCTGGGTAATAAGCGTTGGCAATTTAACCGCCAGTCAGGCTTTCTTTCACAGATGTGGATTGGCGATAAAAAACAAC
TGCTGACGCCGCTGCGCGATCAGTTCACCCGTGCACCGCTGGATAACGACATTGGCGTAAGTGAAGCGACCCGCATTGAC
CCTAACGCCTGGGTCGAACGCTGGAAGGCGGCGGGCCATTACCAGGCCGAAGCAGCGTTGTTGCAGTGCACGGCAGATAC
ACTTGCTGATGCGGTGCTGATTACGACCGCTCACGCGTGGCAGCATCAGGGGAAAACCTTATTTATCAGCCGGAAAACCT
lacZ-F1 ACCGGATTGATGGTAGTGGTCAAATGGCGATTACCGTTGATGTTGAAGTGGCGAGCGATACACCGCATCCGGCGCGGATT
GGCCTGAACTGCCAGCTGGCGCAGGTAGCAGAGCGGGTAAACTGGCTCGGATTAGGGCCGCAAGAAAACTATCCCGACCG
CCTTACTGCCGCCTGTTTTGACCGCTGGGATCTGCCATTGTCAGACATGTATACCCCGTACGTCTTCCCGAGCGAAAACG
GTCTGCGCTGCGGGACGCGCGAATTGAATTATGGCCCACACCAGTGGCGCGGCGACTTCCAGTTCAACATCAGCCGCTAC
AGTCAACAGCAACTGATGGAAACCAGCCATCGCCATCTGCTGCACGCGGAAGAAGGCACATGGCTGAATATCGACGGTTT
lacZ-F2 CCATATGGG**GATTGGTGGCGACGACTCCT**GGAGCCCGTCAGTATCGGCGGAATTCCAGCTGAGCGCCGGTCGCTACCATT **lacZ-R7**
ACCAGTTGGTCTGGTGTCAAAAATAATAATAACCGGGCAGGGGGGATCTAAGCTCTAGATAAGTAATGATCATAATCAGC
CATATCACATCTGTAGAGGTTTTACTTGCTTTAAAAAACCTCCCACACCTCCCCCTGAACCTGAAACATAAAATGAATGC SV40 pA
AATTGTTGTTGTTAACTTGTTTATTGCAGCTTATAATGGTTACAAATAAAGCAATAGCATCACAAATTTCACAAATAAAG

CATTTTTTTCACTGCATTCTAGTTGTGGTTTGTCCAAACTCATCAATGTATCTTATCATGTCTGGATCCGGAATAACTTC loxP
GTATAGCATACATTATACGAAGTTATGTTTAAACGGCGCGCCCCGGAATTCGCCTTCTGCAGGAGCGTACAGAACCCAGG
GCCCTGGCACCCGTGCAGACCCTGGCCCACCCCACCTGGGCGCTCAGTGCCCAAGAGATGTCCACACCTAGGATGTCCCG
CGGTGGGTGGGGGGCCCGAGAGACGGGCAGGCCGGGGGCAGGCCTGGCCATGCGGGGCCGAACCGGGCACTGCCCAGCGT
GGGCGCGGGGGCCACGGCGCGCGCCCCCAGCCCCCGGGCCCAGCACCCCAAGGCGGCCAACGCCAAAACTCTCCCTCCTC Beta actin promoter
CTCTTCCTCAATCTCGCTCTCGCTCTTTTTTTTTTTCGCAAAAGGAGGGGAGAGGGGGTAAAAAAATGCTGCACTGTGCG
GCGAAGCCGGTGAGTGAGCGGCGCGGGGCCAATCAGCGTGCGCCGTTCCGAAAGTTGCCTTTTATGGCTCGAGCGGCCGC
GGCGGCGCCCTATAAAACCCAGCGGCGCGACGCGCCACCACCGCCGAGACCGCGTCCGCCCCGCGAGCACAGAGCCTCGC
Neo-F1 CTTTGCCGATCCTCTAGAGTCGAGATCCGCCGCCACCATGATTGAACAAGATGGATTGCACGCAGGTTCTCCGGCCGCTT
GGGTGGAGAGGCTATTCGGCTATGACTGGGCACAACAGACAATCGGCTGCTCTGATGCCGCCGTGTTCCGGCTGTCAGCG
CAGGGGCGCCCGGTTCTTTTTGTCAAGACCGACCTGTCCGGTGCCCTGAATGAACTGCAGGACGAGGCAGCGCGGCTATC *Neo*
GTGGCTGGCCACGACGGGCGTTCCTTGCGCAGCTGTGCTCGACGTTGTCACTGAAGCGGGAAGGGACTGGCTGCTATTGG
GCGAAGTGCCGGGGCAGGATCTCCTG**TCATCTCACCTTGCTCCTGC**CGAGAAAGTATCCATCATGGCTGATGCAATGCGG **Neo-R2**
CGGCTGCATACGCTTGATCCGGCTACCTGCCCATTCGACCACCAAGCGAAACATCGCATCGAGCGAGCACGTACTCGGAT
GGAAGCCGGTCTTGTCGATCAGGATGATCTGGACGAAGAGCATCAGGGGCTCGCGCCAGCCGAACTGTTCGCCAGGCTCA
AGGCGCGCATGCCCGACGGCGAGGATCTCGTCGTGACCCATGGCGATGCCTGCTTGCCGAATATCATGGTGGAAAATGGC
CGCTTTTCTGGATTCATCGACTGTGGCCGGCTGGGTGTGGCGGACCGCTATCAGGACATAGCGTTGGCTACCCGTGATAT
TGCTGAAGAGCTTGGCGGCGAATGGGCTGACCGCTTCCTCGTGCTTTACGGTATCGCCGCTCCCGATTCGCAGCGCATCG
CCTTCTATCGCCTTCTTGACGAGTTCTTCTGAGCGGGACTCTGGGGTTCGAAATGACCGACCAAGCGACGCCCAACCTGC
CATCACGAGATTTCGATTCCACCGCCGCCTTCTATGAAAGGTTGGGCTTCGGAATCGTTTTCCGGGACGCCGGCTGGATG
ATCCTCCAGCGCGGGGATCTCATGCTGGAGTTCTTCGCCCACCCCCCGGATCTAAGCTCTAGATAAGTAATGATCATAAT SV40 pA
CAGCCATATCACATCTGTAGAGGTTTTACTTGCTTTAAAAAACCTCCCACACCTCCCCCTGAACCTGAAACATAAAATGA
ATGCAATTGTTGTTGTTAACTTGTTTATTGCAGCTTATAATGGTTACAAATAAAGCAATAGCATCACAAATTTCACAAAT
AAAGCATTTTTTTCACTGCATTCTAGTTGTGGTTTGTCCAAACTCATCAATGTATCTTATCATGTCTGGATCCGGGGGTA
PNF CCGCGTCGAGAAGTTCCTATTCCGAAGTTCCTATTCTCTAGAAAGTATAGGAACTTCGTCGAGATAACTTCGTATAGCAT FRT
ACATTATACGAAGTTATGTCGAGATATCTAGACCCAGCTTTCTTGTACAAAGTGGTTGATATCTCTATAGTCGCAGTAGG loxP
R2R CGG

TTTTATTATTTGGGACTCGTTGTAAAGGCTTTACATGGTAGAAACTCATTGGAAAGGGAACCTCCTCCACGAATGTGAAG Critical region
CAATTCTCTTAGAAGTTAATATCCTGAAAACAAAATGCGCTCTTCCCCTCTGCTCTACTCAATACTGTTTTCTTACCCTC
CTAAACCTGCCTTCAGCCAAGCATGGCAGTTCACACTT**TAATCACAGCACTCTGGAGG**CAGATCTCTGTGAGTTCAAGGC **mKLi2R13**
CAGCCTGGTGTACATAGTGAGTTCTAGACCAGCCAGGTTATGTAGTGAAAACAACAAACCTGTCTTCGATTCTGCCTCAG
ATGCTGGTGGTGTTTGGACTCACAGTAATCTCGTGCTGGGTTCTTCCTAAGTGTCTTCATTCTTTGTTTTTAGATGCTGA Exon 3
TGAGAGCAGATGCCAACAGGGGAAGACACTTTATGGAGCTGGCTTGAGAACTGAGGGAGAAAATCACCTCCGGCTTCTTG
mKL3F1 CAGGAAGCCTGCCTTTCCACGCCTGTCGGGCTGCCTGCTGCCGGGACTCTGCCTGCCACGCTCTATGGTGGCTGGAAGGG
ATGTGCTTTCAGGCTGACTGCAGTAAGCCCCAGAGCTGCCAGCCTTTTAGGACAGACTCTTCCAATTCCATGCTGATCAT
TTTTCAAAAATCCCAAACTACAGATGATTTGGGCCTTCTGCCTGAAGATGATGAACC**ACATCTTCTGAGGCTAGGCT**GGG **mKL3R3**
mKL3F2 GCAGGACATCGTGGAGGAGGCAGAGCCTTCTTGGGGCTCCCCTCACCCTTTCTGTACCCTCTAGTCACCACCAGAGCTTA
CTCAGGGATCGGCAGAAGAGAGATCTCAGTGTGGTACCTACACATGGAGCGATGCAGCATTCTAAAGTGAATCACTCGGA
GGAAGCAGGTGCTCTGAGTCCCACCTCTGCAGAGGTAAGCGAGCAGGTTCCTAGGGGTAGAACTTAAATCTATTCCTCCG
CATCTATGCATGAGCAACTGAGCTGATGCCCGTGGCCAGTGCTCACAGGCTGGCGTATGCGTAAAGCTGGCTGGCATTTC
TTCAGAGGTCCTAGGAATGTTCATTTTATATAGGTCTGACAACTGTTTTCGTTAGAG

CGGAAAGCTGCTCATTATCTGTTCTAAGCTGGTTCTTGGCTACAGGTAAGTGCTGCTGTGT
GGGTGTGAGCCTCTACCAATGGCTTTGCAGTGTTTTCTGCCAGATTGCAAAAACAGTCATT
GGTGTAGCTGCCGTCAAGTTTTCCTCCTCCATCTCCTGCCT

Segment of intron 3 replaced by loxP cassette in KO1 allele

mKLi3R2

loxP cassette

GAGATGGCGCAACGCAATTAATGATAACTTCGTATAGCATACATTATACGAAGT**TATGGTCTGAGCTCGCCATC**AGTTCA loxP
loxP3R

ATAAACACATTCCTCATGAGCTGGTTTCTATTCCAGTCTGAATGAACCATGCTGGTATTGTCCACCACGATCTGACCTGC 3’ arm
TGGGCTGGGATGCATGAAGTCTGTCAACCAAGAGGTGTCTGGGGACATTCTCACCAAGCTTATATGCTAAGGGACATGCC
ACCAATAGAGTTTATTCTGCTTATTATGTTTCTTCACTGTTGCTCTTCCTGCACAATGATTAGAAGGAGTGACTCATTCT
GTGCTCAGTCTGCCTTGTTACTGTGACTTATTTTTGTATTTGTTTTGTCCTAGTTGTCTCTGTCAAGTCCAAACCAGAGT
GGGGAG**GTTACTACTTGCCAGGCACT**GTGTGTGCTAATCCCTGTACTATGTCATTTTGTTTAATTTTCATGGCAATATTT **mKLi3R4**
CCAAGTAGGTATTATTAGTTTTGTTTTACTAGTGAGGAAACTGAGGCCCAAAAGAGGTGAAGCAGCTTGCCTGAAATCAT
mKLi3F4 GCAGCTACTGAGCAGCAGCTCTGGCATCGAAAGCCAGCCTGACTATTTAGGAAGAACTCTGAGTGAAGCATTAAGAGGAT
CCCACTCTGACTCAGTATGCCCACGACCACTTAAGGAAACCCACCATCAATGTGAGATACTTCTCTAAGCACTAGAAGAG
TGGAAGCATGTAAGACATGGCCTGTGTCCTCAGGGGACACTGACACAAGAAGCTAAGCCTTCCCAGTGGTGTGTGATGAT
GGCGTTAGCAGAACCTAGCCCTGGGCATGTTACACTGCTCTCCTACTGAGCAGTGCTAAGTGCTTAAGGATGACGCCAGC
TTTCCCTCATCAAAACAGGATGTTCTGAGCCCTCAGTGGACCCCTGAAAATGGTACTGGTTCTCTGTATAGACTGCTGCA
TGACGTTCCTGTATCTGTGCTTGGTGCCGTACAGAGGTCAGAATGAGGCATTACATCTCCTGGAATGGGAATTCTGGACA
GTTGTGAGCTGCCCTGTGCGTTCTGGGAATCAAACCCTGGTCCTCGGCAAGAACTTATCTGCTGAGCCATCTCGTCAGCT
CCTGGAGAGAACTTT**CACATTCTCACTCAGGTCGA**GGCATTTTGGCTTTTTTTTTTTTCCTTATCAGGAATTCTAGTATT **mKLi3R6**

ACTATTCTTTTGCTTTGGGACATGTATTAAGTAAACTATGTGCTACTTTAATATAAGCCTACACTATGGGAGAGTTGATA
CCTCATTATGTAACTGGATGCACGGGAAAAAGATTTATGTCCCAGTTCAGATGAAGTAGAATGGTAAGAGACCTTCGTGA
TGCTTACCATAAGGGTAATGCTACTTAAAACTTACGAATTGTTCATTTCTGGAATTTACCATTTTTTAACTTCTTATTAG
TTCCTTGGGGATTTCGTCCGATTTGTTTTGATCATATTCTCCCTCCCCCACTTTTCTACTCACCCAATTGTTTGTCTGTT
TATTTATTTATTTATTTATTTACATCCCAATTGCAGTCCCTCCCCCAACCCCCGTCTACTCCTCCTCTTCAGAAAGGGGC
AGGCCTCCCATGGATATTGACCAGCCAAGCAATAGCAAGTTGCAGTAAGACTAGGCAGTCCTCTCCTATTAAGGCTGGAT
GAGGCAACCCAGTAAGAGGAAAGGGTCTGAAAAACAGGCAACAGAGTCAGAGACATCCCTGCTTCACCTGTTAGGAGTCT
CACATGAAGACCAAGCTACCCAGCTGTGACATATGTGCAGAGACCTTATGATATAGGTCAGACCTATACAGGCTCCCTGG
TTGGTGGTTCAGCCTCTGTGAGCCCCTATGAGCCCAGGTTAGTTGATTCTGTGGGTCTTCTTGTGGTGTCCTTGACCCCT
CTTGCTCCTATGATCCTTCCTCCCCATCTGTTTCCATCACTTGCTGGCTGAAGCCTCTCTGATGTCAACTGTCACCCATC
TATGAGCATAGCAGGATATCACTAGGAATCGTCTCATTCACTTTTTTTCTTGCTAGTTGTATTTGTTTCTATCCTAGGAC
TCTGGGCTGTCCGCCCTCTGGGTCTGGTCCTTCAGGCAGTGTCAGGCATGGGCTTCCTCTCATAGCCAGACTCTCTAGCT
GGATCAGACATTGGTTGGCCACTTCCACAATTTCTGTGCTCCTTTTACCCTGGTGCTTCCCGTAAGATGGACAAATTGTA
GGTTGAAGGTTTTGTAGCTGGAATGTCTCCACTGGAAGTCTTGCCTGGTTACAAGAGATGGGCCAGGTCAGGCTCTGTAT
CCCCCATTTCTACAAGTCTCAGCTAGGGTCACCATCATAGATTCCTGGGAGGTTATTTTGTCTGTTCTTGTTTTGTTTTT
GCACTGGGTTTCTAGCTTGTTCCAGAGATGACCCCAATTCCAGGTGTCTCTCCCAGTACTCTCTCCCTCCATCCTCCTTG
CATGTGATCCCTCCTGTTCCCTCCCCACCTGCCCCCAGCCCACGGTGACCTCTACCTACTCTTCCGTTCAGACCCTTCTC
CCATCCACCCATCATGTCTATTTTATTTCCCCTTCACAGTGAGAATCCTGTGTTTCCCTTTGGTCCCTCCTTGTTACTTA
ACTTCTCTGGGTCTGTGGATTGTAGCTTGGCTATCCTTTACTTTACAGCTAGTGAGAACACACCATGTTTGCCTTTCTGG
GTCTGGGTCACCTCCCTCAGGATGATGTTTTTCTAGTTCTATCCATTTGCCTACAGATCTCATGATGTTGGGGTTTTTTT
TTTTAATGCATCTTGTCTCAAATTTATTTGTAAACACTGCTCATCTGCAAATAGTGTATAGGTAGGTGTGTCACAGCAGT
CCATCTGTCTTCCCATTGGCAGAAGACTTTTCTATGGGGTCTTTACAGGGGCCTGGACATGTTTGGGAGCCTGAGATGTA
GCTGAAGCCTAGACCTTAGCTGGAGCTGCAGCCTCTGCCTTGGTTTGAACCTTGGGCTTTGGTTGGCAGAGTCCTCGACC
CTTGGCCATGTAGCTTCAAATCTACTTCCCAAGCTTGGGGTGATCGATGAAAGCCAGACGGCTGAGTCTGTGGCTGGGGC
CCCTTGGCATCTTGGGCTTGATGGCCTGAGGCTTCACCAAGTCCTTGGTGGCCTCTGCTCGTGCACTCACTGCCTTTGCA
mKLi3F11 TTGCTGGCCTGCATCTTCTTCAGGCCTTTCTTGTGTTACTCAGGAACTTGGTTGGGATCAACTCCCTTAAGAGATTCGTA
TCTTTGTGACTAGGGTTTCTTTTATTTAAAATTTTTATTTATTTATTTATTTTATTTTTGGTTGTTCGAGACAGGGTGTC
TCTGTATAGCCCTGGCTGTCCTGGAACTCACTTTGTAGACCAGGCTGGCCTCGAACTCAGAAATCTGCCTGCCTCTGCCT
CCCAAGTGCTGGGATTAAAGGCGTGTGCCACCACGCCGGGCCAATACTCCATTTTCTTAGTGCTGGGGATTAAAGG**TATG**
CACTACCACTACCTGGCTTTTGTTTTGTTTTTGTAACATTTTAAAAATTCTTAGTGATTCATTTATTTTATTTTATTTTA **mKLi3R11**
TTTTATTTTTAAGATTTATTATTACACATAAGTACACTGTAGCTGTCTTCAGACACACCAGAAGAGGATGTCAGATCTCA
TTACGGGTGGTTGTGAGCCACCATGTGGTTGCTGGGATTTGAACTCAGGACCTTTGGAAGAGCAGTCAGTTCTCTTACCT
GCTGTGCTATCTCACCAGCCTGTGACTAGGGTTTCTTGATGCCATTTCTGTGCCGTTTGTGGGACTGGTTGTGTGTGGTG
TGGTTCTTGAACTTGGACTTGAACTTGGCATGTCTGCATGGTAACCCAAGGATCCCTCAGTGCTTGGGACCAATAGAGAA
AGAGTTCATGATGTCATTTTAAAAAAATAACTAAATAATACTCCATTGTATGACCTCATAAAACAAATTTGGCAATGTTC
CTTCTGTTTATATTTTGTGGGATAATTTGAAGAGTATTGGTATTAGCTCTTCTTTGAAAGTCTCTTAGCACTGTCAATAA
GACAAAAAGGCCACCAACAGATTGGGAAAGGATTTTTACCAATCTTAAATCTGATAGGGGACTAATATCCAATAAATACA
AAGAGCTCAAGAAGCTGGACTCCAGAAATTCAAATAACCCCATTAAGAAATGGGGTACAGAGCTAAACAAAGAATTCTCA
ACTGAGGAATACCAAATGGCTGAGAAGCACCTGAAAAAATGTTCAACATCCTTAATCATCAGGGAAATGCAAATCAAAAC
AACCTTGAGATTCCACCTCACACCAGTCAGAATGGCTAAGATCAAAAATTCAGGTGACAGCAGATGCTGGCGAGGATGTG
GAGAAAGGG**GAACACTCCTCCACTGCTGG**TGGGATTGCAAGCTTGTACAATCACTCTGGAAGTCAGTCTGGAGGTTCCTC **mKLi3R14**
AGAAAATTGGACATAATACTACTGGAAGATCCAGCATACCTCTCCTGGGCATATACCCAAAAGAAGTTCCAACTGGTAAT
AAGAACACATGCTCCACTATGTTCATAGCAGCCTTATTTATAACAGCCAGAAGCTGGAAAGAACCCAGATGTCCCTCAAC
AGAAGAATGGATACAGAAAATGTGGTACATTTGCACAATAGAGTACTACTCAGCTATTAAAAACAACTGATTTGTGAAAT
TCTTGGACAAATGA

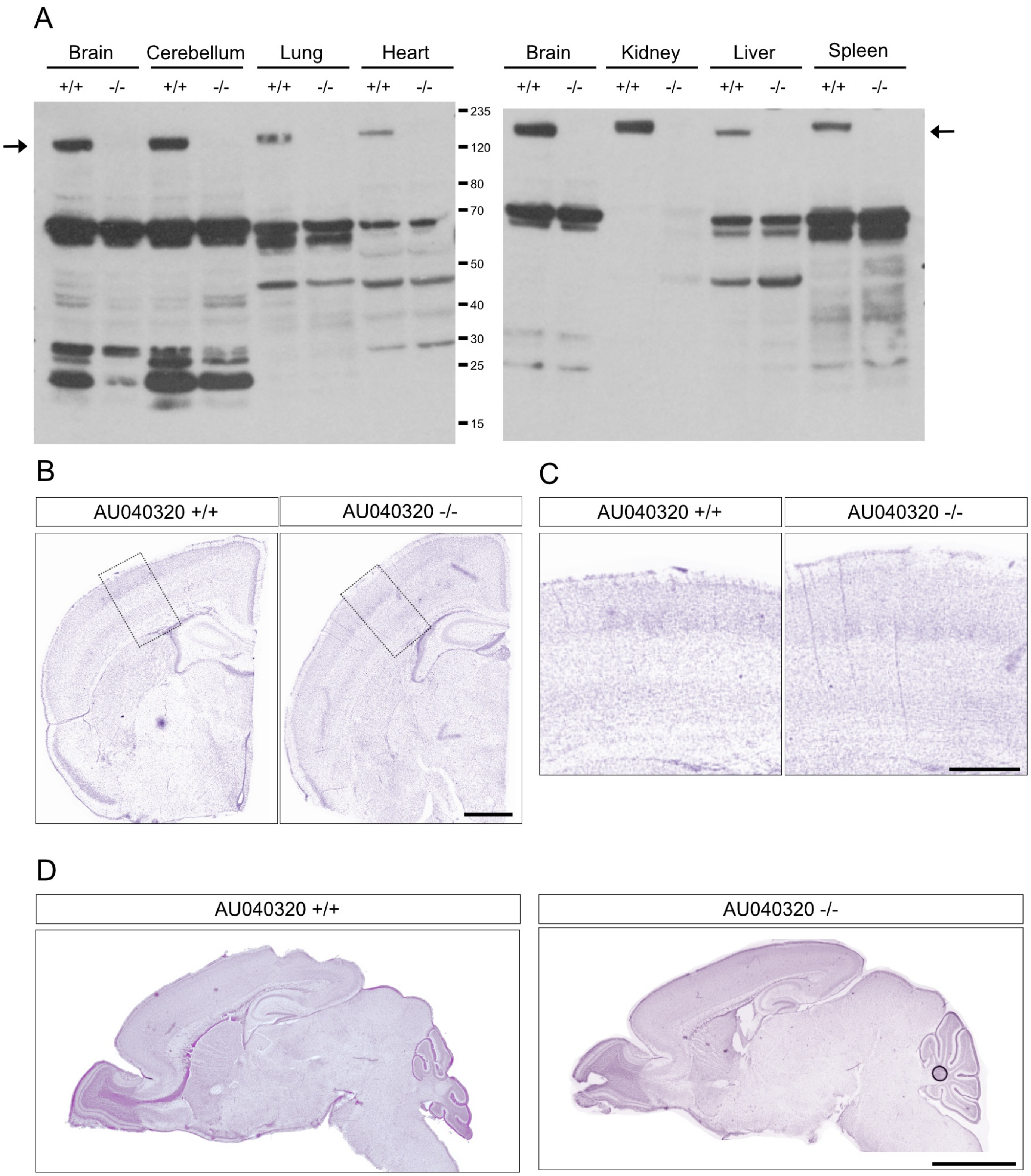
A
Brain
Cerebellum
Lung
Heart
Brain
Kidney
Liver
Spleen
+/+
-/-
235
120
80
70
50
40
30
25
15
B
AU040320 +/+
AU040320 -/-
C
AU040320 +/+
AU040320 -/-
D
AU040320 +/+
AU040320 -/-

Figure S3

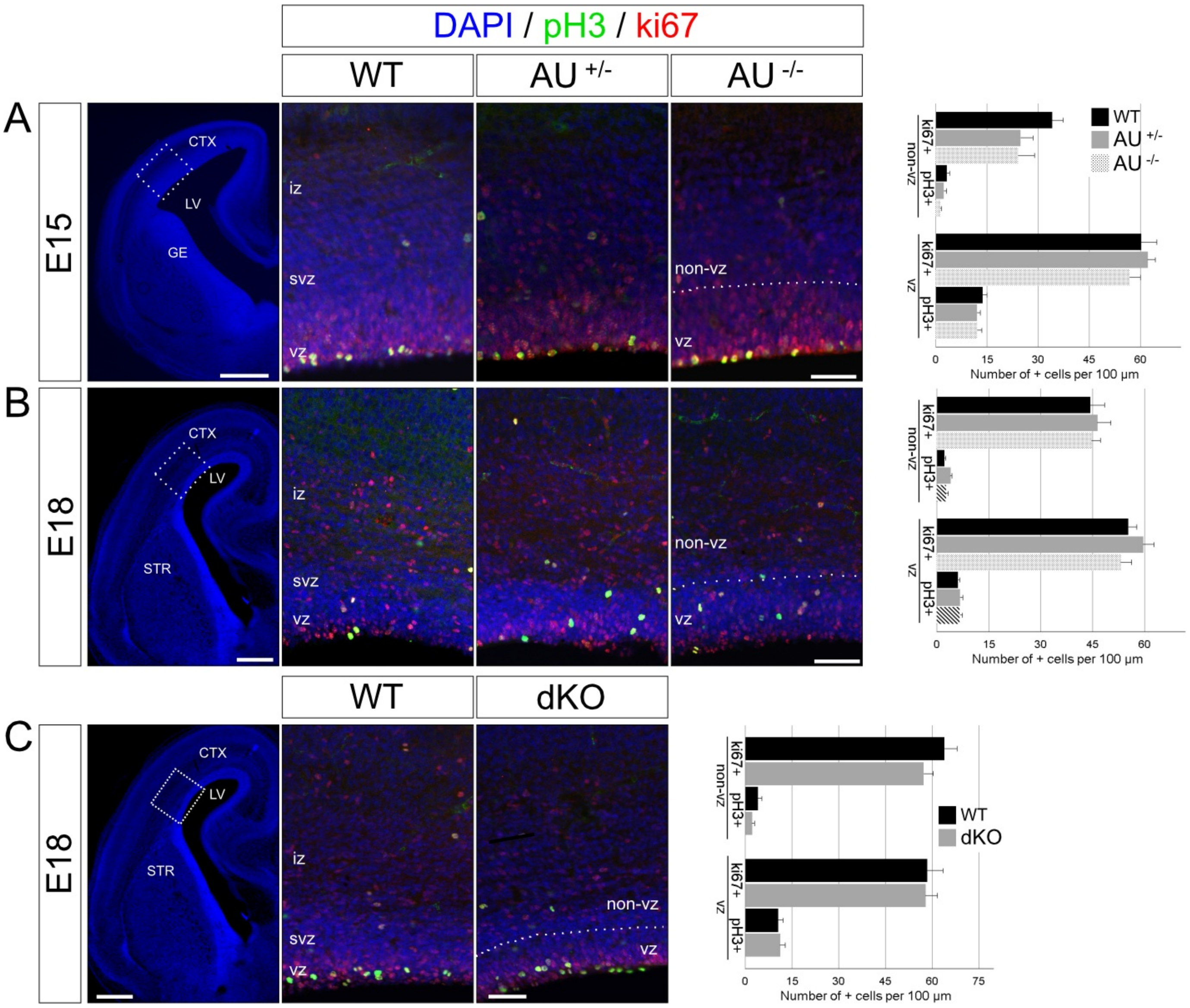

DAPI / pH3 / ki67
WT
AU +/-
AU -/-
A
E15
CTX
LV
GE
iz
svz
vz
non-vz
WT
AU +/-
AU -/-
ki67+
pH3+
non-vz
vz
Number of + cells per 100 µm
B
E18
CTX
LV
STR
C
dKO

Figure S4

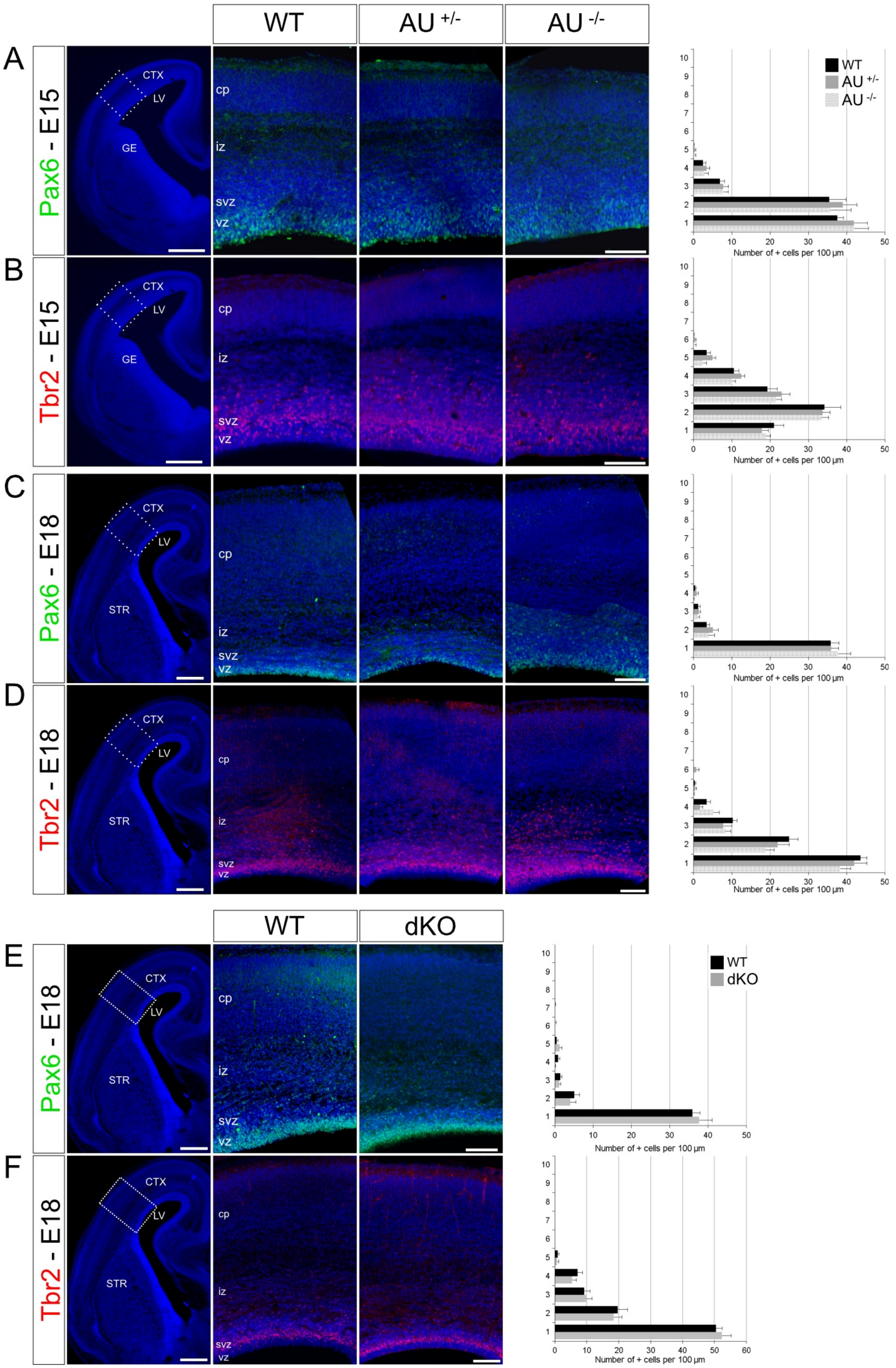

WT
AU +/-
AU -/-
A
Pax6 - E15
CTX
LV
GE
cp
iz
svz
vz
WT
AU +/-
AU -/-
Number of + cells per 100 µm
B
Tbr2 - E15
C
Pax6 - E18
STR
D
Tbr2 - E18
E
Pax6 - E18
WT
dKO
F
Tbr2 - E18

Figure S5

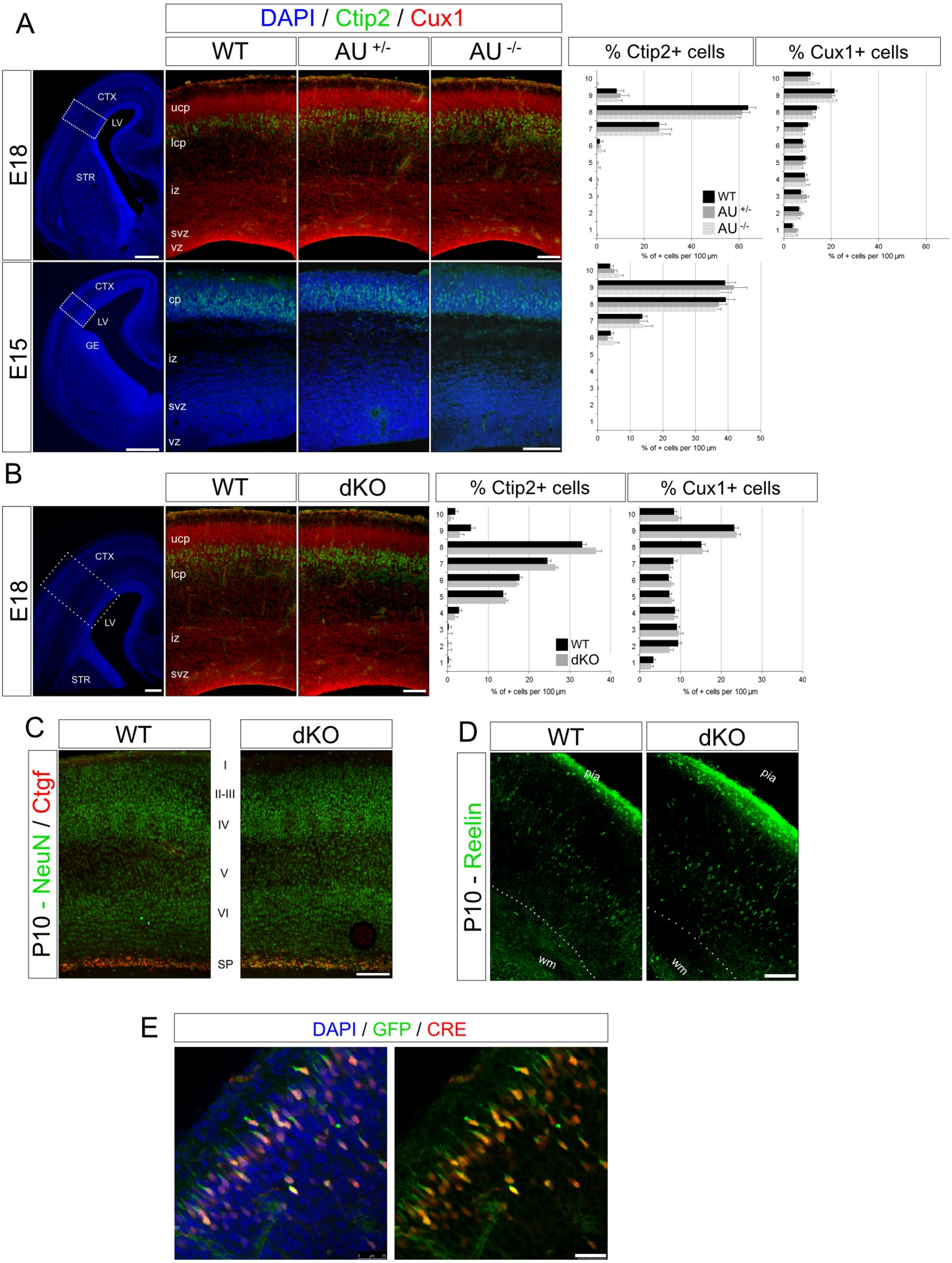
A
DAPI / Ctip2 / Cux1
WT
AU +/-
AU -/-
% Ctip2+ cells
% Cux1+ cells
E18
E15
CTX
LV
STR
GE
ucp
lcp
iz
svz
vz
cp
WT
AU +/-
AU -/-
% of + cells per 100 µm
B
WT
dKO
% Ctip2+ cells
% Cux1+ cells
E18
CTX
LV
STR
ucp
lcp
iz
svz
% of + cells per 100 µm
C
WT
dKO
P10 - NeuN / Ctgf
I
II-III
IV
V
VI
SP
D
WT
dKO
P10 - Reelin
pia
wm
E
DAPI / GFP / CRE

Figure S6

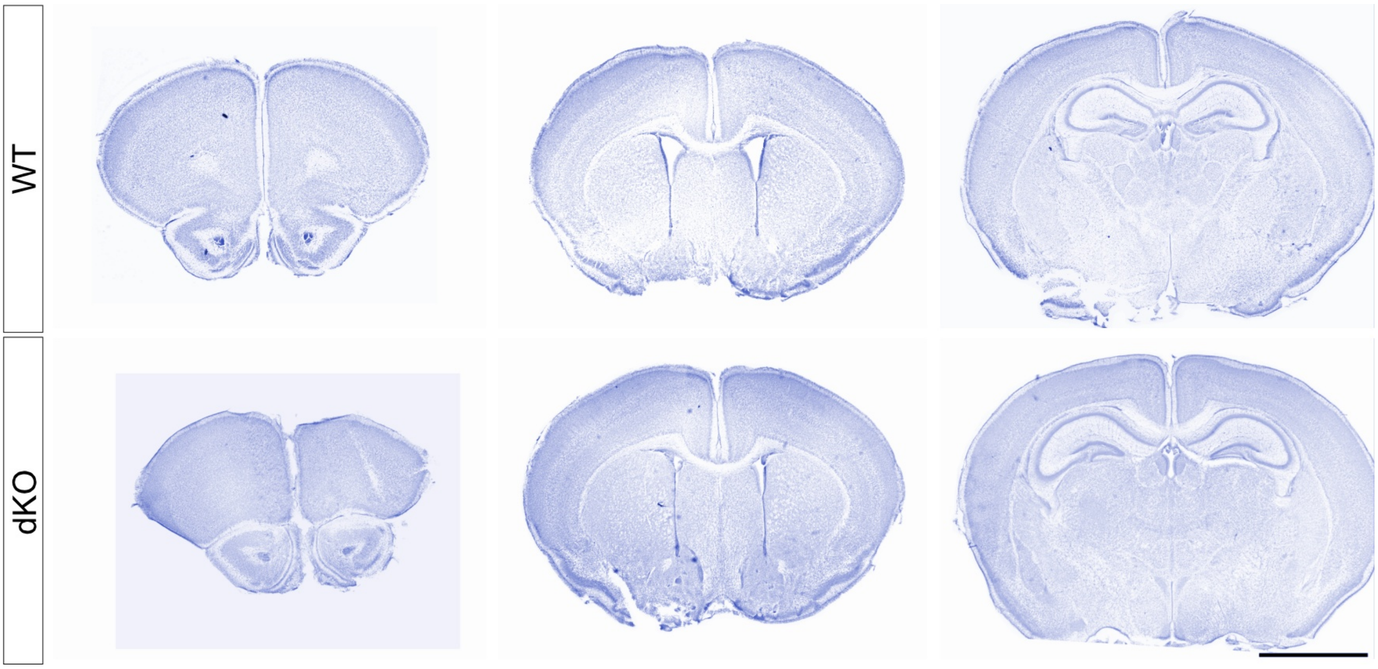

Figure S7

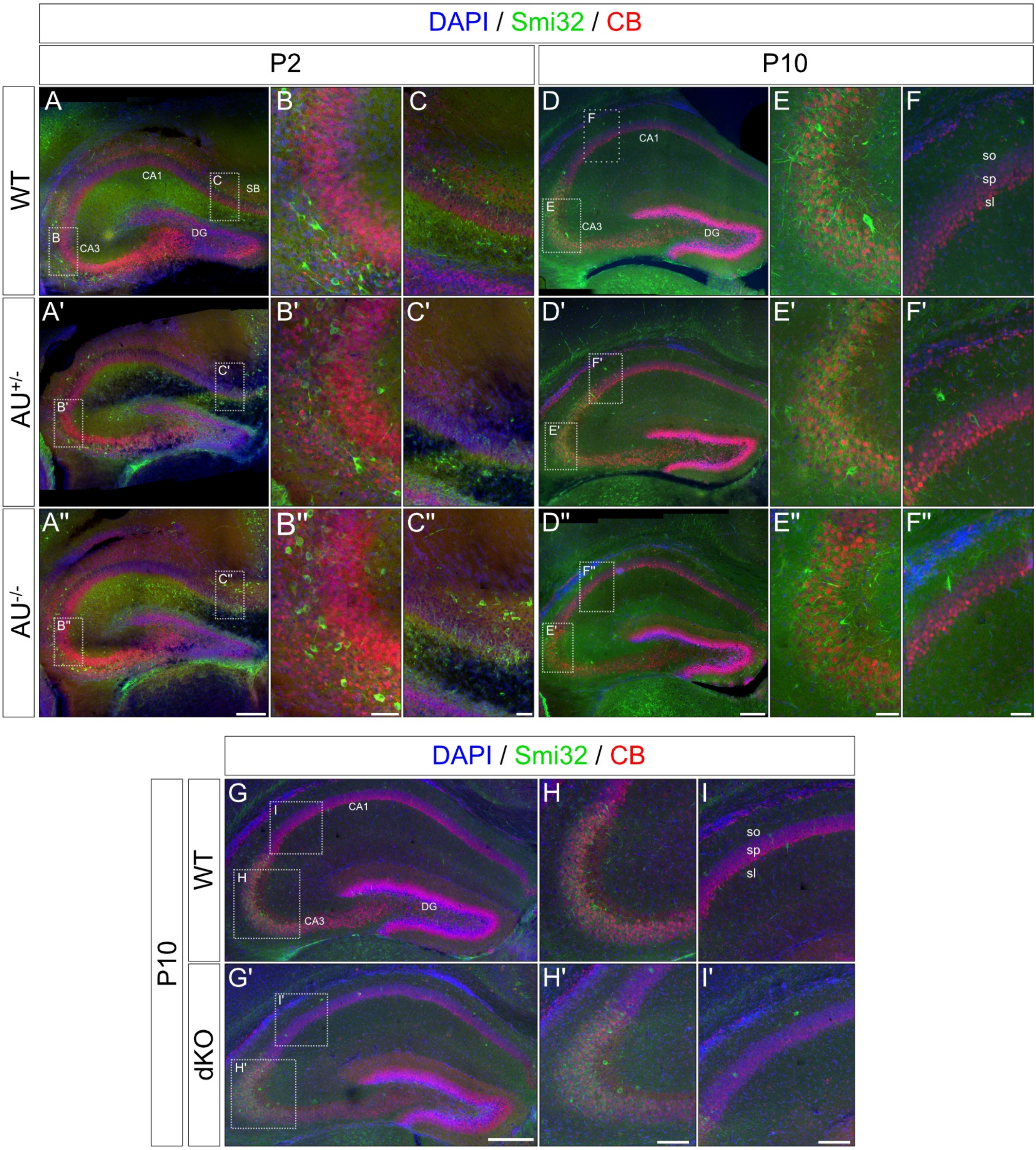
DAPI / Smi32 / CB
P2
P10
WT
AU+/-
AU-/-
A
B
C
D
E
F
A'
B'
C'
D'
E'
F'
A''
B''
C''
D''
E''
F''
CA1
CA3
DG
SB
so
sp
sl
DAPI / Smi32 / CB
P10
WT
dKO
G
H
I
G'
H'
I'

Figure S8

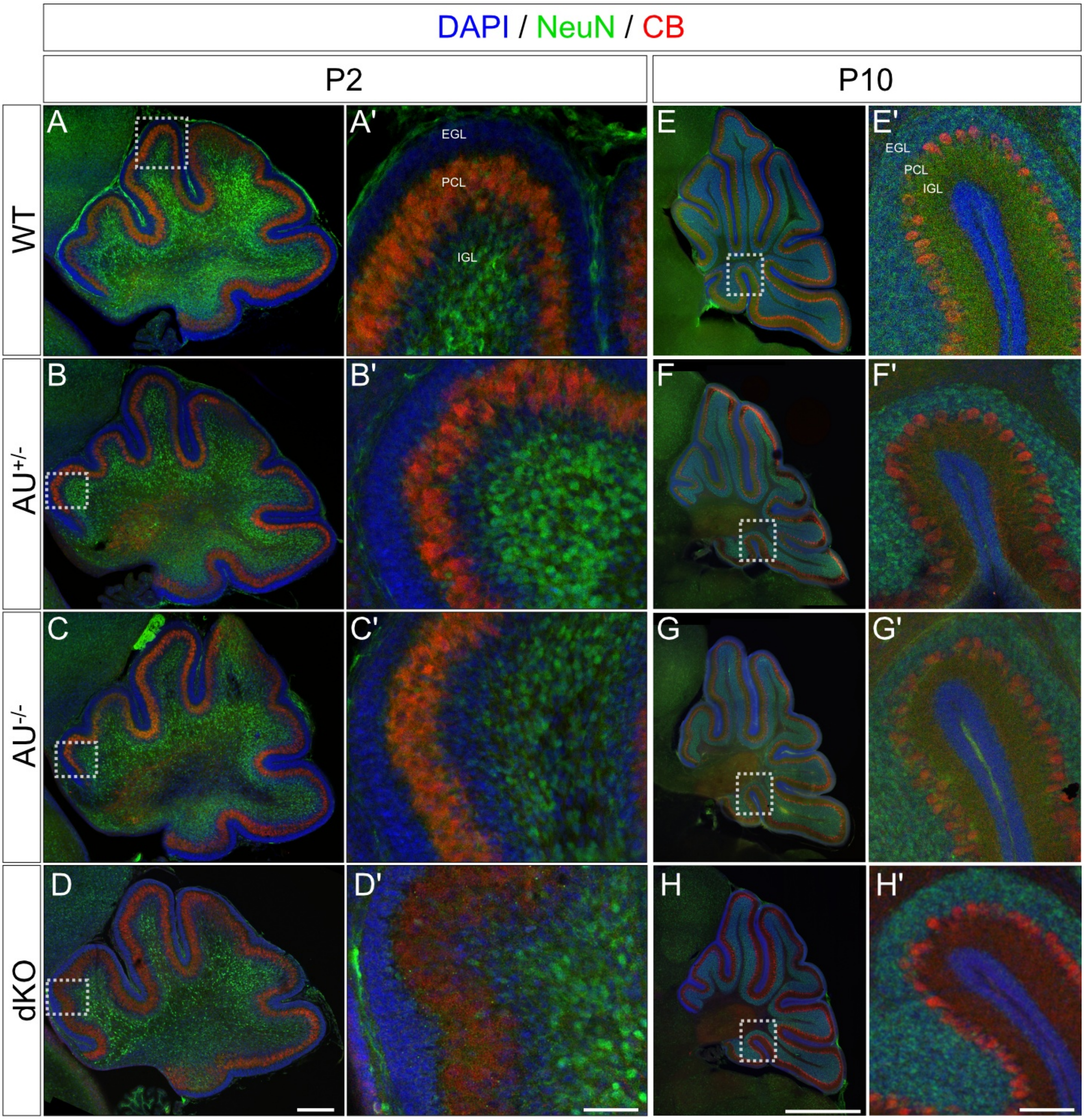
DAPI / NeuN / CB
P2
P10
WT
AU+/-
AU-/-
dKO
A
A'
E
E'
B
B'
F
F'
C
C'
G
G'
D
D'
H
H'
EGL
PCL
IGL
EGL
PCL
IGL

Figure S9

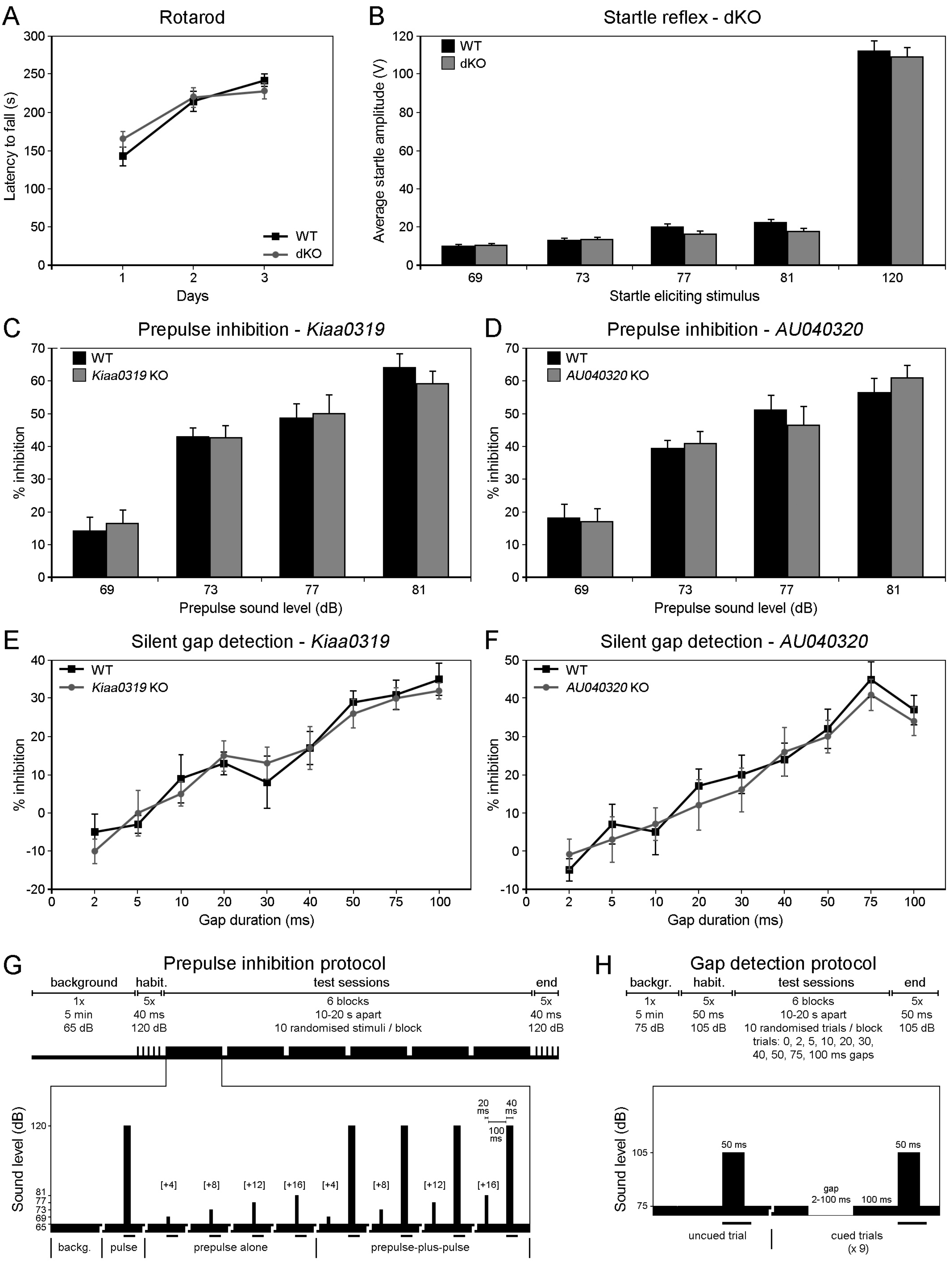
A
Rotarod
Latency to fall (s)
Days
WT
dKO
B
Startle reflex - dKO
Average startle amplitude (V)
Startle eliciting stimulus
WT
dKO
C
Prepulse inhibition - Kiaa0319
% inhibition
Prepulse sound level (dB)
WT
Kiaa0319 KO
D
Prepulse inhibition - AU040320
% inhibition
Prepulse sound level (dB)
WT
AU040320 KO
E
Silent gap detection - Kiaa0319
% inhibition
Gap duration (ms)
WT
Kiaa0319 KO
F
Silent gap detection - AU040320
% inhibition
Gap duration (ms)
WT
AU040320 KO
G
Prepulse inhibition protocol
background
habit.
test sessions
end
1x
5 min
65 dB
5x
40 ms
120 dB
6 blocks
10-20 s apart
10 randomised stimuli / block
5x
40 ms
120 dB
Sound level (dB)
20 ms
40 ms
100 ms
[+4]
[+8]
[+12]
[+16]
backg.
pulse
prepulse alone
prepulse-plus-pulse
H
Gap detection protocol
backgr.
habit.
test sessions
end
1x
5 min
75 dB
5x
50 ms
105 dB
6 blocks
10-20 s apart
10 randomised trials / block
trials: 0, 2, 5, 10, 20, 30,
40, 50, 75, 100 ms gaps
5x
50 ms
105 dB
Sound level (dB)
50 ms
gap
2-100 ms
100 ms
uncued trial
cued trials
(x 9)

Figure S10

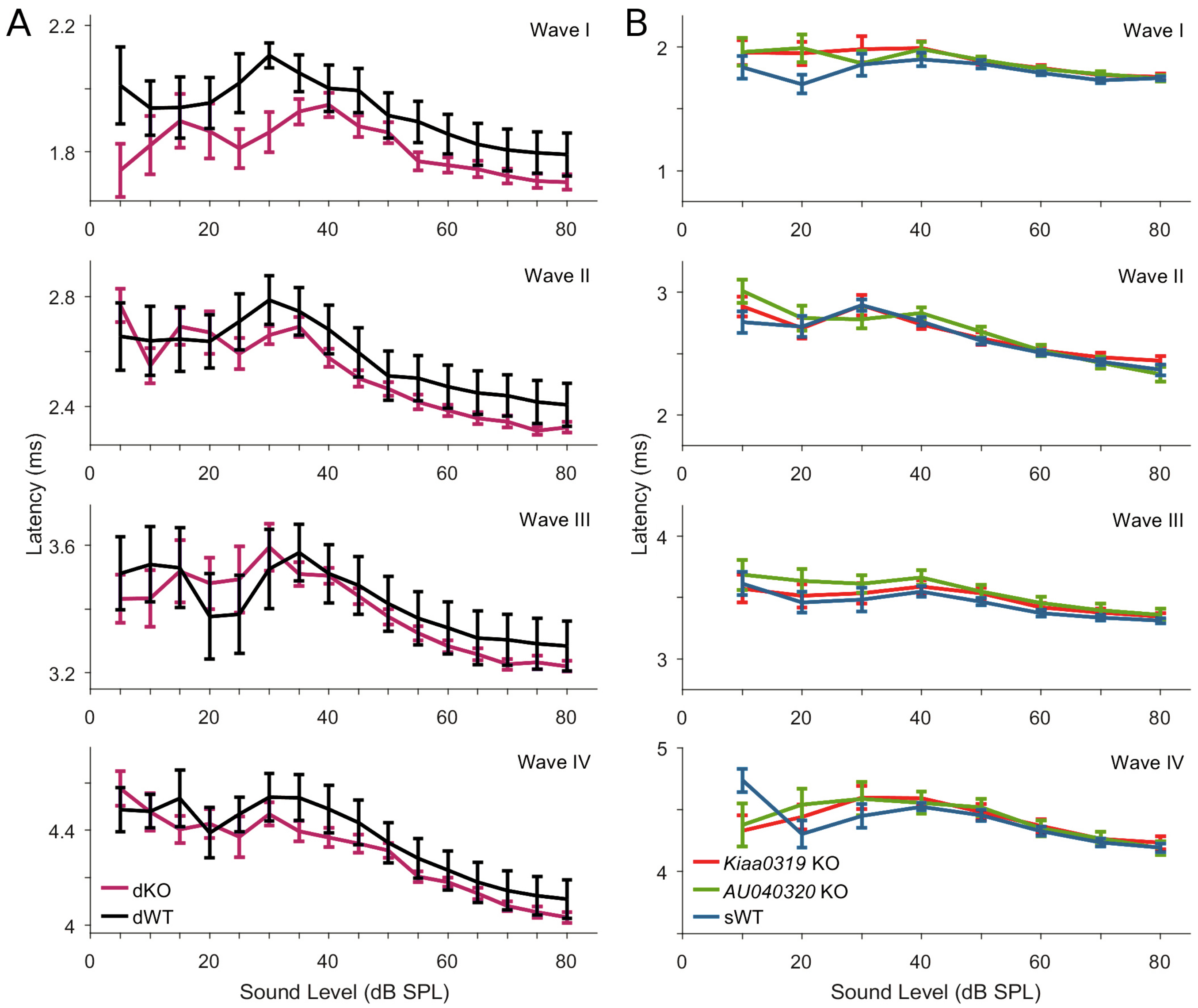
A
B
Wave I
Wave II
Wave III
Wave IV
Latency (ms)
Sound Level (dB SPL)
dKO
dWT
Kiaa0319 KO
AU040320 KO
sWT

Figure S11

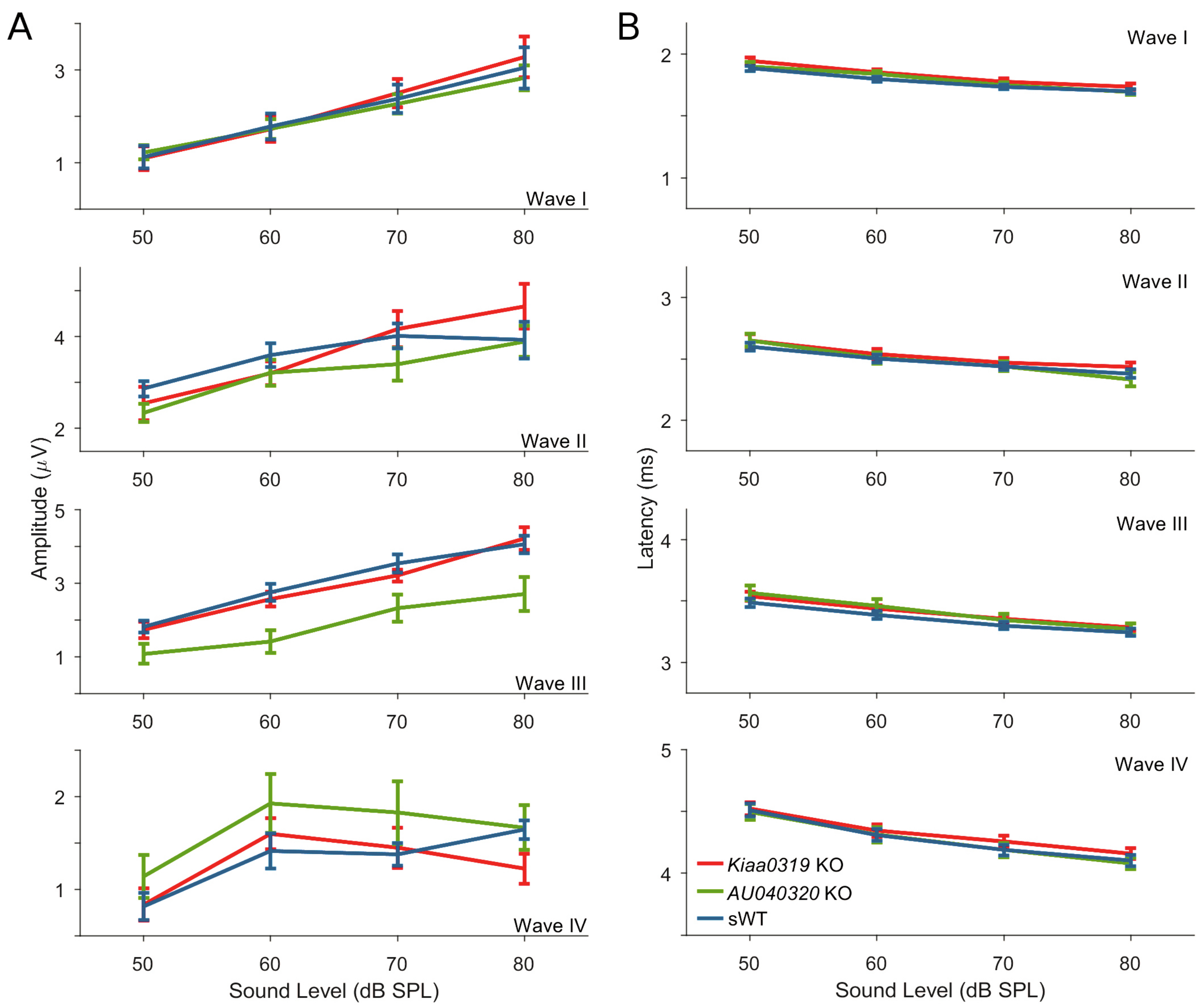
A
B
Amplitude (μV)
Latency (ms)
Wave I
Wave II
Wave III
Wave IV
Sound Level (dB SPL)
Kiaa0319 KO
AU040320 KO
sWT

Figure S12

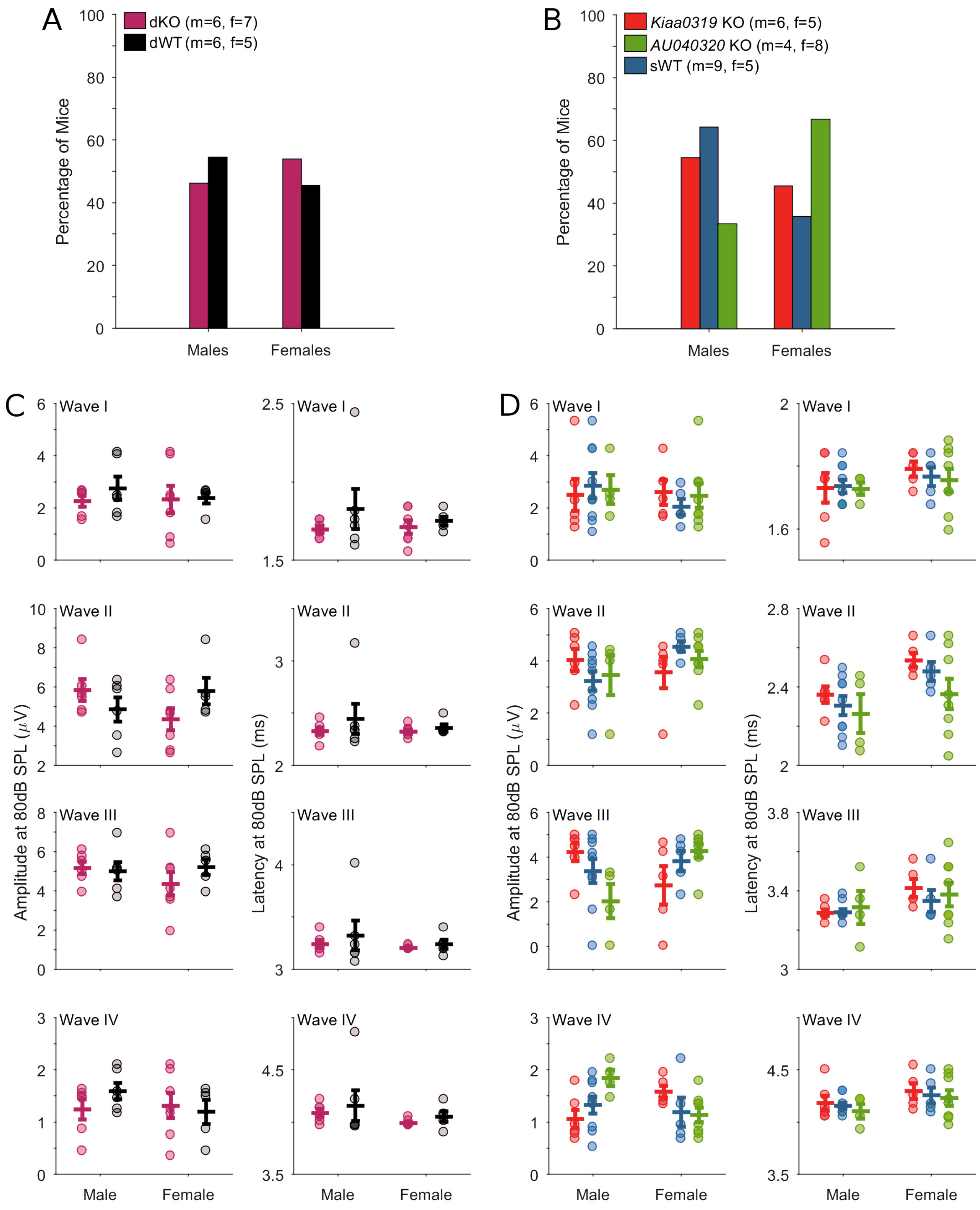
A
dKO (m=6, f=7)
dWT (m=6, f=5)
Percentage of Mice
Males
Females
B
Kiaa0319 KO (m=6, f=5)
AU040320 KO (m=4, f=8)
sWT (m=9, f=5)
C
D
Wave I
Wave II
Wave III
Wave IV
Amplitude at 80dB SPL (μV)
Latency at 80dB SPL (ms)
Male
Female

Figure S13

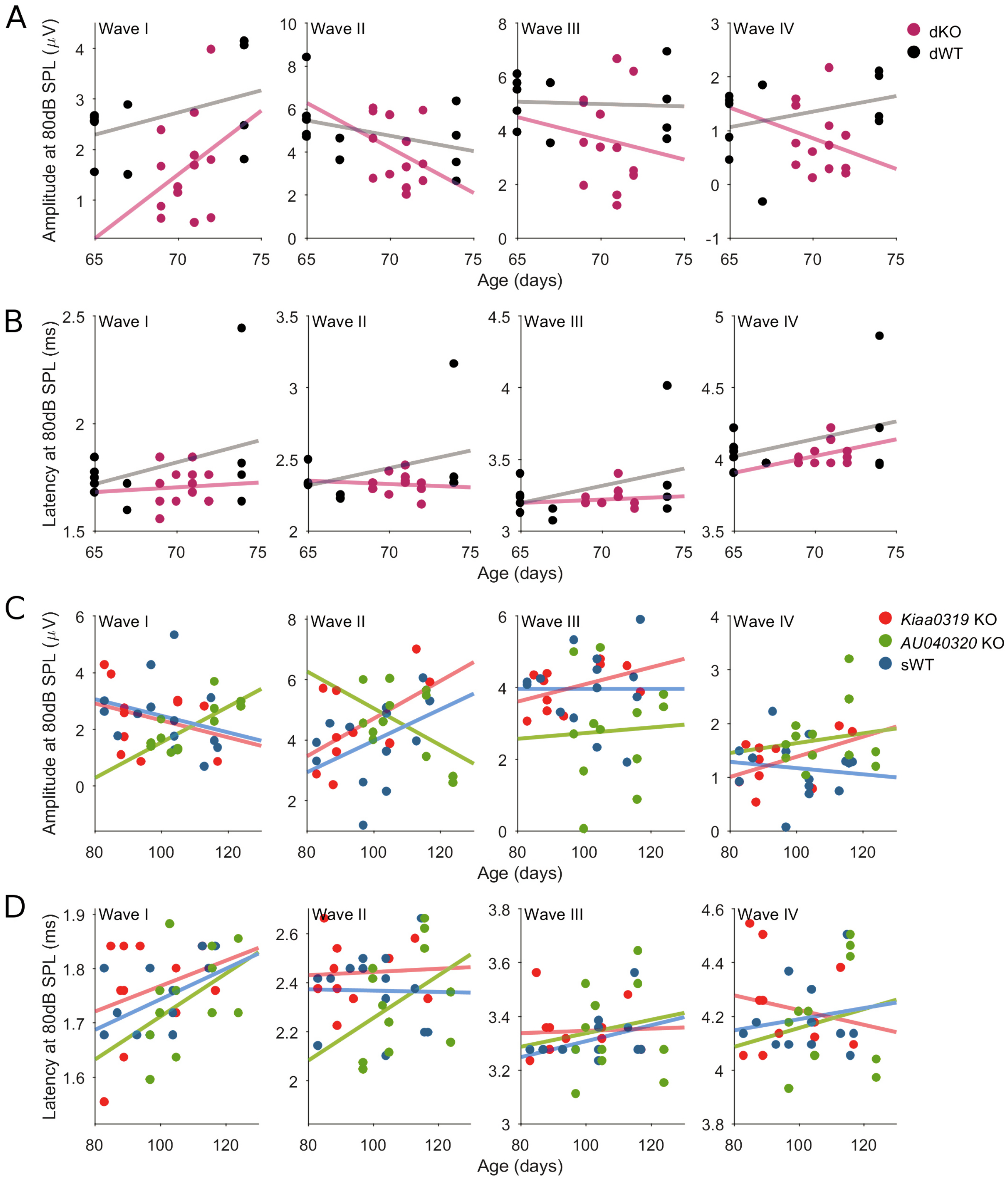
A
B
C
D
Wave I
Wave II
Wave III
Wave IV
dKO
dWT
Kiaa0319 KO
AU040320 KO
sWT
Amplitude at 80dB SPL (μV)
Latency at 80dB SPL (ms)
Age (days)

Figure S14

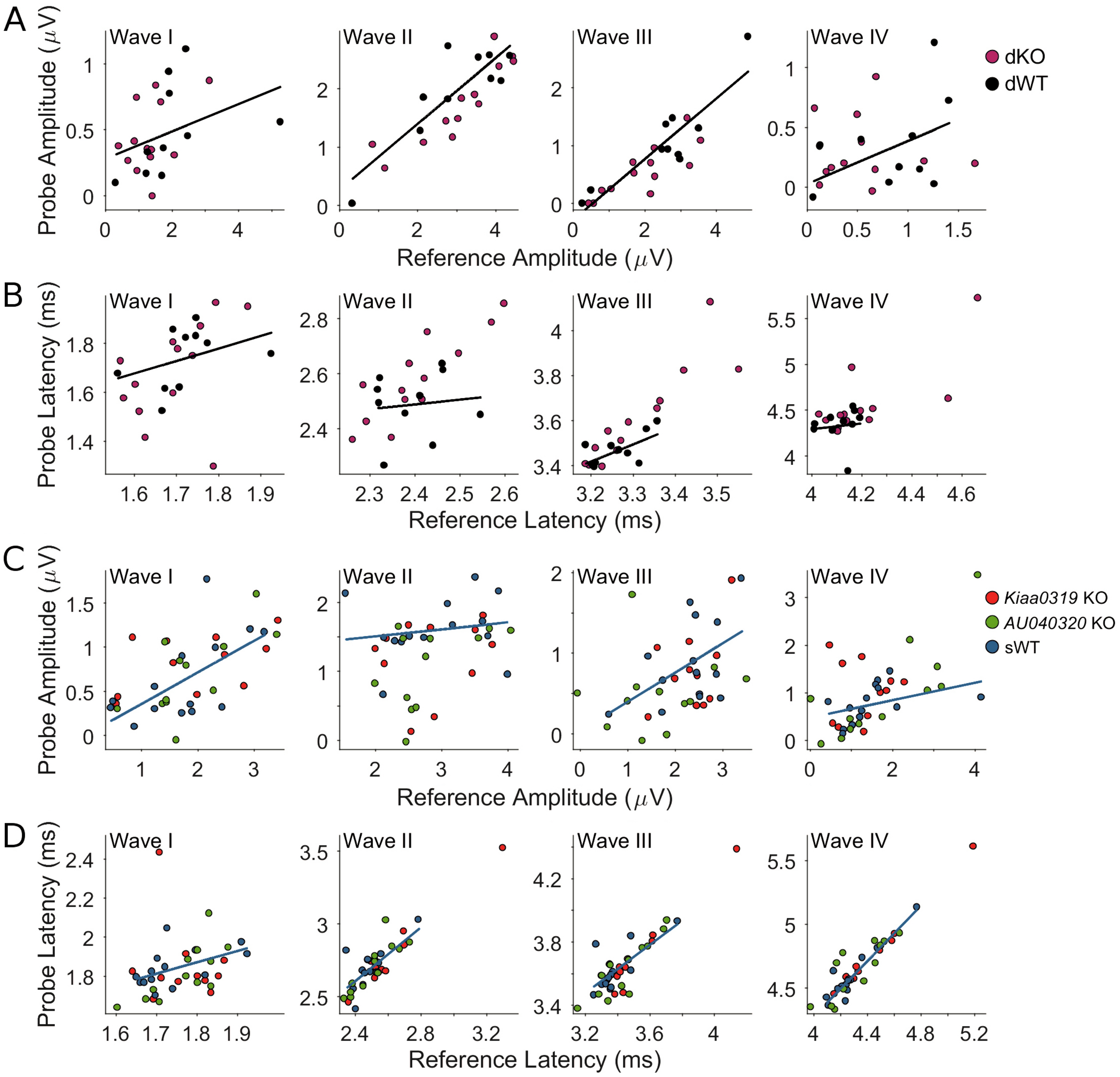

A
B
C
D
Wave I
Wave II
Wave III
Wave IV
dKO
dWT
Kiaa0319 KO
AU040320 KO
sWT
Probe Amplitude (μV)
Probe Latency (ms)
Reference Amplitude (μV)
Reference Latency (ms)

Figure S15
